# Biomodulators and Skin Boosters in Skin Rejuvenation: Mechanisms, Applications, and Future Perspectives

**DOI:** 10.3390/ijms27219647

**Published:** 2026-10-28

**Authors:** Zhiyuan Ding, Caijun Jin, Huizhen Ming, Jung Hee Shim, Vo Tien Huy, Pham Ngoc Chien, Chan Yeong Heo

**Affiliations:** 1 Department of Plastic and Reconstructive Surgery, College of Medicine, Seoul National University, Seoul 03080, Republic of Korea; jiwon4@snu.ac.kr (Z.D.); jcjking96@snu.ac.kr (C.J.); ming921@snu.ac.kr (H.M.); 2 Department of Plastic and Reconstructive Surgery, Seoul National University Bundang Hospital, Seongnam 13620, Republic of Korea; xmylife@empas.com; 3 Department of Anatomy, VNU University of Medicine and Pharmacy, Vietnam National University, Hanoi 100000, Vietnam; dr.richardhuy@gmail.com

**Keywords:** biomodulators, skin boosters, hyaluronic acid, polynucleotides, collagen remodeling, skin rejuvenation

## Abstract

Biomodulators and skin boosters comprise heterogeneous preparations whose biological activity and clinical effects require separate evaluation. This structured narrative review distinguishes mechanistic plausibility from human comparative findings for skin aging, skin quality, and atrophic acne scars. A reproducible PubMed/MEDLINE search completed on 16 October 2026, followed by predefined screening, source retrieval, and eligibility adjudication, identified 159 comparative reports, each contributing one specified result. Risk-of-bias assessments classified 123 results as High and 36 as Some concerns; none was Low overall. Selected formulations and procedure-assisted regimens showed benefits for particular outcomes, whereas other comparisons were null or discordant. Hydration, tissue remodeling, clinical appearance, and patient satisfaction were treated as distinct outcomes. Retrospective result selection, heterogeneous interventions and outcomes, four access-unresolved reports, and the absence of a prospective protocol constrain confidence. Clinical claims should remain specific to the product, protocol, indication, comparator, and outcome investigated.

## 1. Introduction

Skin aging is a multifactorial biological process marked by gradual changes in the structure and function of the skin. It reflects the combined influence of intrinsic factors, including chronological aging and genetic background, and extrinsic factors, such as ultraviolet radiation, environmental pollution, smoking, poor nutrition, and lifestyle-related oxidative stress [1,2]. These influences lead to epidermal thinning, degradation of the dermal matrix, reduced collagen and elastin content, impaired fibroblast activity, decreased skin hydration, and loss of mechanical elasticity [3,4]. Clinically, aged skin is characterized by wrinkles, laxity, dryness, uneven texture, and reduced regenerative capacity [5].

The dermal extracellular matrix is essential for preserving skin structure, integrity, and biomechanical function [3]. Among its major components, type I and type III collagen provide tensile strength and structural support, whereas elastin fibers are responsible for elasticity and recoil [6,7]. With aging, fibroblast function gradually declines, resulting in reduced collagen production and impaired matrix maintenance [8]. At the same time, increased activity of matrix metalloproteinases accelerates collagen breakdown and further contributes to dermal matrix deterioration [9]. The age-related decrease in hyaluronic acid also compromises the water-binding capacity of the dermis, leading to dryness, fine lines, and an overall decline in skin quality [10]. Based on these mechanisms, therapeutic approaches that improve dermal hydration, stimulate fibroblast activity, and enhance extracellular matrix remodeling have become increasingly relevant in aesthetic and regenerative dermatology [2].

Traditional aesthetic treatments have mainly focused on volume restoration, wrinkle correction, or surface resurfacing. However, recent approaches have shifted toward improving overall skin quality through regenerative mechanisms [11,12]. In this context, biomodulators and skin boosters have gained increasing attention. Biomodulators, also referred to as biostimulators, are injectable or bioactive agents designed to stimulate biological responses such as fibroblast activation, collagen synthesis, angiogenesis, and tissue remodeling [2,13]. Common biomodulators include poly-L-lactic acid, calcium hydroxylapatite, polycaprolactone, polynucleotides, and platelet-derived products [14,15,16,17].

Skin boosters are another important category of minimally invasive treatments used to improve dermal hydration, elasticity, smoothness, and texture [18]. Unlike conventional fillers, which primarily provide immediate volume augmentation, skin boosters are usually injected into the superficial or mid-dermis to enhance skin quality through hydration and microenvironmental improvement [19,20]. Hyaluronic acid-based skin boosters are the most widely used, while formulations containing amino acids, peptides, vitamins, antioxidants, polynucleotides, or other bioactive molecules have also been developed [19,21].

Although biomodulators and skin boosters are often considered within the same field of regenerative aesthetics, they serve different therapeutic roles [11]. Biomodulators are generally intended to produce gradual and long-lasting dermal improvement by stimulating neocollagenesis and extracellular matrix remodeling [22]. Skin boosters, in contrast, are mainly used to enhance dermal hydration, elasticity, and superficial skin texture [23]. Despite these differences, the two approaches share the common goal of improving skin quality through biological or microenvironmental modulation rather than simple volume replacement [2]. In clinical practice, they may also be used in a complementary manner. Combination treatments with energy-based devices, microneedling, lasers, or conventional fillers have further extended their use in facial rejuvenation, acne scar treatment, skin laxity improvement, and overall dermal revitalization [24].

Despite the expanding clinical use of biomodulators and skin boosters, interpretation of the literature remains difficult because terminology, product composition, administration protocols, clinical indications, and levels of evidence vary substantially across studies [19,23,25]. The present review therefore uses a structured framework to examine these interventions without assuming therapeutic equivalence across product categories. Human clinical evidence was identified through a reproducible product-focused PubMed/MEDLINE search completed on 16 October 2026 and was interpreted according to the investigated formulation, administration route, indication, comparator, follow-up period, and outcome. Mechanistic and preclinical studies are discussed separately to provide biological context rather than direct evidence of clinical efficacy. This approach allows areas of mechanistic overlap to be described while preserving the distinction between biological plausibility and demonstrated human clinical outcomes [26,27,28,29,30,31].

## 2. Review Methodology

### 2.1. Review Design and Scope

This review was conducted as a structured critical narrative review. The updated search used the report or publication as the document unit. Comparative human reports formed the primary clinical evidence stream, and each retained report contributed one specified result defined by the comparison and selected outcome; the assessment time was recorded when ascertainable from the accessible source. Reports were not assumed to represent independent trials; companion publications were linked to the same underlying trial when identifiable.

Contextual human reports, noncomparative primary estimates, and reports with unresolved design or eligibility questions were recorded separately. Mechanistic, in vitro, animal, review, and regulatory literature was used to provide biological and translational context and was not treated as direct evidence of clinical efficacy. Findings from these evidence streams were not considered interchangeable.

### 2.2. Literature Search

A reproducible PubMed/MEDLINE search was completed on 16 October 2026. Nine product-focused strategies covered HA-based skin boosters, PN/PDRN, PLLA, CaHA, PCL, PRP/PRF, EV/exosome-containing preparations, secretome or conditioned-medium preparations, and generic skin-booster or biostimulation concepts. The publication-date ceiling was 16 October 2026; no language, human-study, or trial-design filter was applied at retrieval.

Intervention terms were combined with concepts for skin aging, rejuvenation, wrinkles, skin quality, photoaging, dermal remodeling, and atrophic acne scars. The nine searches returned 5963 record occurrences. Exact PMID deduplication removed 1015 repeat appearances and yielded 4948 unique records. The detailed search strategies and selection methods are provided in Supplementary Table S1.

Complete sources were sought through PubMed-linked repositories, DOI and publisher pages, Seoul National University Library-linked access routes, and lawful local files. Backward citation tracking was used to clarify report relationships or locate source records and was not counted as a separate systematic database search.

This was a structured critical narrative review rather than a prospectively registered systematic review. Screening and source adjudication followed predefined eligibility criteria. Mechanistic and preclinical literature was selected narratively and analyzed separately from human comparative evidence. Further supporting information is provided in Supplementary Tables S1–S4.

### 2.3. Eligibility Criteria

For the comparative human evidence set, reports were eligible when they met all of the following criteria: (1) adult participants undergoing treatment for facial or non-facial skin aging, photoaging, wrinkles, skin laxity, impaired skin quality, or atrophic acne scars; (2) evaluation of HA-based or multicomponent skin boosters, PN/PDRN preparations, PLLA, CaHA, PCL, autologous platelet-derived preparations, or EV/exosome-containing or secretome-derived preparations; (3) injection, intradermal or subdermal delivery, or procedure-assisted delivery when the procedure formed an integral part of the investigated protocol; (4) at least one clinical, instrumental, histological, patient-reported, or safety outcome; and (5) a randomized human comparative design with an interpretable between-group, within-person, split-face or split-body, or protocol comparison. One comparison and selected outcome were specified for each retained report, and the assessment time was recorded when ascertainable from the accessible source.

Contextual human reports and noncomparative primary estimates meeting the population, intervention, and outcome criteria were recorded separately from the comparative evidence set. Individual case reports, reviews, editorials, expert opinions, conference abstracts without sufficient methodological information, purely volumetric filler studies without a prespecified skin-quality outcome, and non-human studies were excluded from the clinical evidence synthesis. Reports that could not be resolved from the available source information were retained as unresolved rather than forced into an eligible or excluded category.

### 2.4. Study Selection and Evidence Classification

Records were screened against the eligibility criteria at title and abstract level and, when potentially eligible, by complete-source or source-level adjudication. Selection used the report as the document unit. Possible companion publications were linked to the underlying trial where identifiable, and report counts were not interpreted as independent-trial counts. Computer-assisted tools supported record organization, rule-based screening, and sensitivity checks.

**Figure 1:**
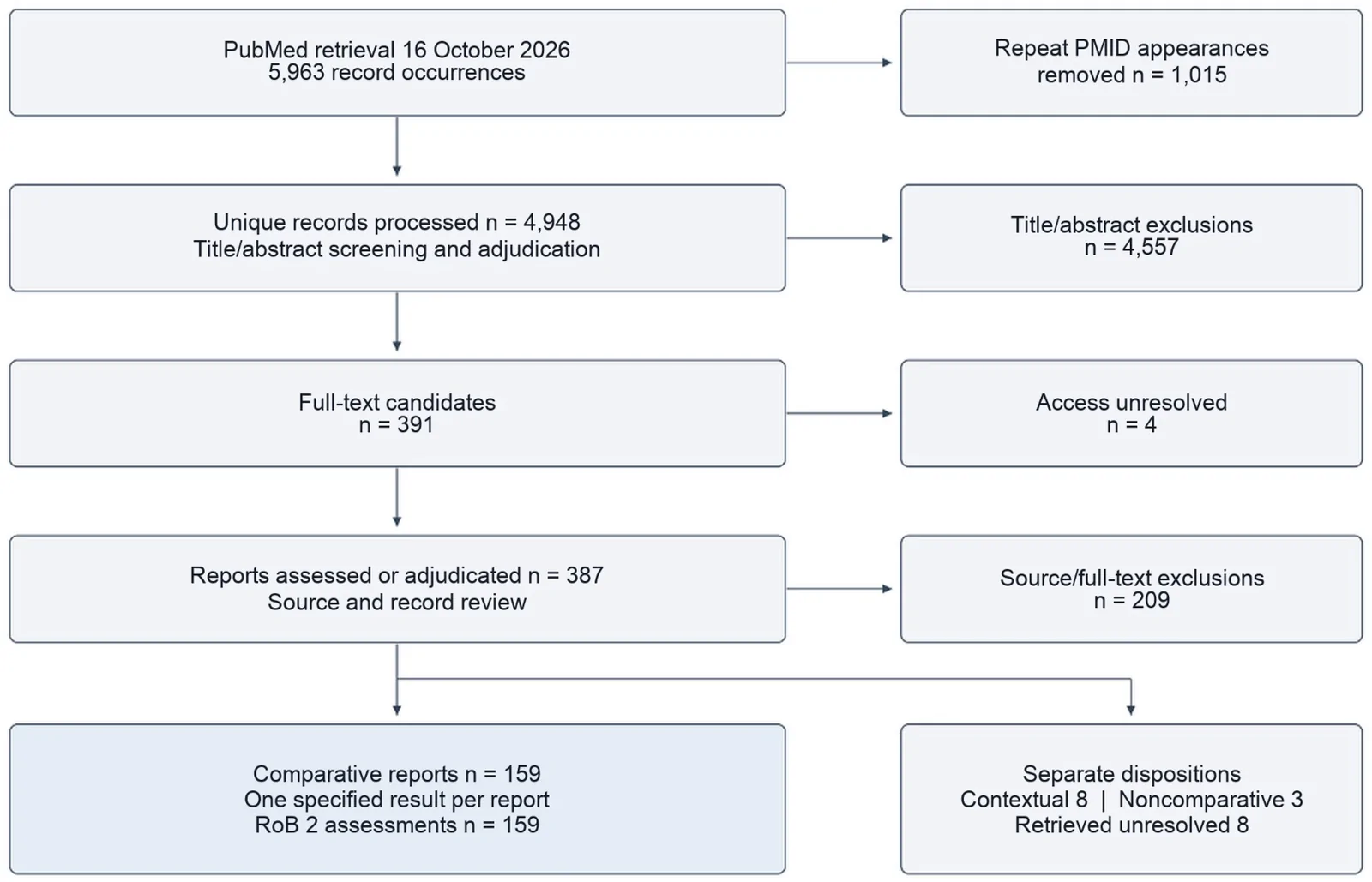
Selection after the 16 October 2026 search and completed access and eligibility adjudication. Of 4948 unique records, 4557 were excluded at title and abstract level and 391 remained full-text candidates. Three hundred eighty-seven reports were assessed or source-adjudicated, and four remained access-unresolved. The assessed or adjudicated set comprised 209 exclusions, 159 comparative reports, eight contextual reports, three noncomparative primary estimates, and eight retrieved-unresolved reports. Each comparative report contributed one specified result.

Of 4948 unique records, 4557 were excluded at title and abstract level and 391 remained full-text candidates. Three hundred eighty-seven reports were assessed or source-adjudicated, and four remained access-unresolved. The assessed or adjudicated set comprised 209 exclusions, 159 comparative reports, eight contextual reports, three noncomparative primary estimates, and eight retrieved reports with unresolved design or eligibility questions. The study selection process is illustrated in Figure 1. Detailed records of source and full-text exclusions are provided in Supplementary Table S4.

Among 195 candidates previously recorded as unavailable, a subsequent access and eligibility recheck of candidates identified by the 16 October 2026 search verified 145 complete texts, identified authoritative full-text links for 42, excluded four records on explicit title or abstract evidence, and left four access-unresolved. Ninety-four met the comparative eligibility criteria and 93 were excluded after source or record review. Records carried forward from the earlier evidence inventory were retained as previously classified unless the updated source-access and eligibility adjudication provided grounds for reclassification; they were not all rescreened de novo. Consequently, the updated screening counts describe the documented update and adjudication rather than a fully repeated assessment of every historical record, and potentially eligible reports may have been overlooked.

### 2.5. Data Extraction

For each of the 159 comparative reports, extraction specified the report identity, underlying trial identity when identifiable, intervention and formulation, complete treatment protocol, comparator, route or procedure, indication, sample size, selected outcome, principal finding, and interpretation limits. Assessment time was recorded when ascertainable from the accessible source. Contextual, noncomparative, and unresolved reports were recorded separately. The detailed clinical comparisons and selected outcomes are provided in Supplementary Table S3.

For combination treatments, intervention categories were distinguished from the complete treatment protocols. Studies involving microneedling, laser treatment, radiofrequency, or additional products were categorized according to the investigated preparation for descriptive purposes. However, their findings were interpreted according to the actual between-group comparison, without automatically attributing the observed clinical effects to an individual biological component.

### 2.6. Risk-of-Bias Assessment

Risk of bias was assessed for the 159 specified comparative results using the Cochrane Risk of Bias 2 (RoB 2) framework for the effect of assignment to intervention. Each assessment identified one report and comparison and recorded the selected outcome and, when ascertainable from the accessible source, the assessment time. The five standard domains were bias arising from the randomization process, bias due to deviations from intended interventions, bias due to missing outcome data, bias in measurement of the outcome, and bias in selection of the reported result. Domain and overall judgments were Low, Some concerns, or High. When available sources did not resolve a signaling question, the judgment followed the RoB 2 no-information pathway. Contextual and noncomparative reports were not incorporated into these result-level counts. Study selection, source adjudication, and risk-of-bias assessment were not independently duplicated. Conditional signaling questions that were not required under the preceding response pathway were coded as “NA” (not applicable), rather than treated as missing assessments. Applicable questions for which insufficient information was available were coded as “NI” (no information). Domain-level and overall judgments were derived using the published RoB 2 decision algorithms, with NI responses incorporated where applicable. Detailed result-level risk-of-bias assessments are provided in Supplementary Table S2.

### 2.7. Evidence Synthesis

Comparative human reports formed the primary clinical evidence stream and were interpreted according to the specific formulation, complete treatment protocol, route, comparator, indication, assessment time, and outcome. Contextual and noncomparative reports were analyzed separately. Mechanistic and preclinical evidence was used only to support biological plausibility. Report counts were not treated as independent-trial counts or certainty grades.

Because substantial heterogeneity remained in product composition, anatomical site, comparator, treatment protocol, follow-up duration, and outcome assessment, quantitative meta-analysis was not considered appropriate. No formal publication-bias test or GRADE assessment was performed. Clinical conclusions were therefore restricted to the product, protocol, indication, comparator, and outcome directly investigated.

## 3. Skin Aging and Dermal Repair and Remodeling

Skin aging is a progressive biological process involving structural, cellular, and molecular changes in the skin [2]. These changes reduce skin thickness, elasticity, hydration, and regenerative capacity [3]. The aging process affects both the epidermis and dermis, but dermal degeneration is particularly important because it directly influences wrinkles, laxity, and loss of skin firmness [32]. Dermal repair and remodeling depend on the coordinated activity of fibroblasts, extracellular matrix components, inflammatory mediators, and vascular support [33]. Therefore, understanding skin structure and the biological mechanism of aging is essential for evaluating biomodulators and skin boosters [34].

In this review, the term ‘regeneration’ is used conservatively. Increased hydration, dermal thickness, collagen expression, neocollagenesis, tissue repair, and extracellular-matrix remodeling are reported as distinct outcomes and are not considered synonymous with true tissue regeneration [35]. Unless restoration toward native tissue architecture and function has been demonstrated, the more specific terms ‘hydration,’ ‘biostimulation,’ ‘repair,’ ‘neocollagenesis,’ or ‘matrix remodeling’ are used [26,36].

### 3.1. Structure of the Skin

The skin comprises the epidermis, dermis, and subcutaneous tissue; among these layers, the dermis is the principal focus of this review because its fibroblasts and extracellular matrix largely determine skin hydration, elasticity, thickness, and mechanical properties [37]. Age-related changes in the subcutaneous compartment contribute to facial volume loss, but these structural changes should be distinguished from changes in dermal skin quality [38].

### 3.2. Intrinsic and Extrinsic Skin Aging

Skin aging is commonly described as the result of two overlapping processes: intrinsic aging and extrinsic aging [2]. Intrinsic aging reflects the gradual biological decline that occurs with chronological aging and genetic programming, and is closely associated with hormonal changes, telomere attrition, mitochondrial dysfunction, cellular senescence, and reduced tissue repair capacity [5,34]. Clinically, intrinsically aged skin tends to appear thin, dry, and less elastic, with fine wrinkles and a gradual reduction in dermal density [3].

Extrinsic aging, in contrast, is driven by environmental and lifestyle-related exposures, including ultraviolet radiation, air pollution, smoking, alcohol consumption, poor nutrition, sleep disturbance, and chronic psychological stress [2,39]. Among these factors, ultraviolet radiation remains the major contributor to premature skin aging and is the principal driver of photoaging [40]. Repeated ultraviolet exposure promotes reactive oxygen species generation, DNA damage, inflammatory signaling, and matrix metalloproteinase activation, which together accelerate the degradation of collagen and elastin fibers in the dermis [9,41].

Photoaged skin is therefore distinct from intrinsically aged skin in both clinical appearance and histological structure. Clinically, it is more often characterized by coarse wrinkles, rough surface texture, irregular pigmentation, telangiectasia, and reduced firmness [40]. Histologically, photoaging is associated with collagen fragmentation, disorganized or abnormally accumulated elastic fibers, epidermal irregularity, and persistent low-grade inflammatory changes [9,41]. Although intrinsic and extrinsic aging arise from different initiating factors, both ultimately converge on extracellular matrix degradation, fibroblast dysfunction, and a reduced capacity for dermal repair and regeneration [33,34].

### 3.3. Role of Fibroblasts, Collagen, Elastin, and Extracellular Matrix

Fibroblasts are central to the maintenance of dermal homeostasis and extracellular matrix integrity. These cells synthesize major matrix components, including collagen, elastin, hyaluronic acid, and proteoglycans, while also participating in matrix turnover through the production of matrix metalloproteinases and their endogenous inhibitors [42,43]. Through these functions, fibroblasts contribute to the preservation of dermal thickness, elasticity, hydration, and mechanical strength.

Collagen is the predominant structural protein in the dermis, with type I collagen providing tensile strength and type III collagen contributing to tissue flexibility and early matrix repair [44]. During aging, collagen production gradually declines, whereas matrix degradation increases. The resulting imbalance promotes collagen fragmentation, dermal thinning, loss of structural support, and the development of wrinkles [45].

Elastin is another key component of the dermal extracellular matrix and is responsible for the elastic recoil of the skin. Aging and chronic ultraviolet exposure lead to progressive disruption of elastic fibers, including fragmentation, disorganization, and loss of normal function [46]. In photoaged skin, abnormal accumulation of elastotic material may occur within the dermis, a characteristic change known as solar elastosis. These alterations compromise skin resilience and contribute to the development of laxity [47].

Beyond its structural role, the extracellular matrix also functions as a dynamic microenvironment that influences cellular behavior. Mechanical and biochemical signals from the surrounding matrix regulate fibroblast activity, growth factor availability, cell migration, and tissue repair. As the extracellular matrix becomes increasingly fragmented and disorganized with aging, the mechanical support required for normal fibroblast function is reduced. This, in turn, may further suppress collagen synthesis and contribute to a self-perpetuating cycle of dermal degeneration and impaired regenerative activity [33].

### 3.4. Inflammation, Oxidative Stress, and Reduced Tissue Repair

Inflammation and oxidative stress are closely involved in the biological processes that drive skin aging. Reactive oxygen species are generated in response to ultraviolet radiation, environmental pollution, mitochondrial dysfunction, and chronic inflammatory activity [48,49,50]. When present in excess, these reactive species can damage cellular proteins, membrane lipids, DNA, and components of the extracellular matrix. Oxidative stress also promotes the activation of signaling pathways associated with increased matrix metalloproteinase expression and reduced collagen synthesis, thereby contributing to progressive dermal matrix degradation [51].

Chronic low-grade inflammation, commonly described as inflammaging, is another important feature of aging skin. Persistent production of pro-inflammatory mediators can impair fibroblast function, accelerate collagen breakdown, and interfere with normal extracellular matrix remodeling. Prolonged inflammatory signaling may also compromise the ability of skin cells to respond appropriately to tissue injury and regenerative stimuli, further limiting the maintenance and repair of the dermal environment [52].

The capacity for tissue repair also declines with age. Aged skin is characterized by delayed re-epithelialization, reduced angiogenic activity, impaired fibroblast migration, diminished responsiveness to growth factors, and decreased collagen deposition [53,54]. Together, these alterations contribute to slower wound healing and incomplete restoration of normal dermal architecture following injury or therapeutic intervention.

These age-related changes provide part of the biological rationale for the use of biomodulators and skin boosters in regenerative aesthetics [20,55,56]. Their clinical effects, however, are likely to vary according to baseline skin condition, product composition, administration technique, and the capacity of the treated tissue to respond to intervention.

## 4. Definition and Classification

Biomodulators and skin boosters are used as minimally invasive approaches to improve skin quality through hydration, biostimulation, tissue repair, and extracellular-matrix remodeling [20,28]. Because the terms ‘skin booster,’ ‘biomodulator,’ ‘biostimulator,’ and ‘regenerative injectable’ are not standardized and are not mutually exclusive, this review uses them as pragmatic functional descriptors rather than formal regulatory or mechanistically exclusive categories [57]. The dominant clinical objective is therefore emphasized rather than an absolute product label. Certain HA formulations may combine hydration with viscoelastic or tissue-support effects, whereas particulate agents such as PLLA, CaHA, and PCL may combine material-related effects with inflammatory signaling, neocollagenesis, and subsequent extracellular-matrix remodeling [18,58].

### 4.1. Biomodulators/Biostimulators

Biomodulators, often referred to as biostimulators, are injectable or bioactive agents designed to promote endogenous regenerative activity within the skin [59]. Their effects are primarily associated with fibroblast stimulation, collagen synthesis, and extracellular matrix remodeling, leading to gradual improvements in dermal structure and tissue quality [58]. In contrast to conventional fillers, which mainly provide immediate volumetric correction, biomodulators exert their effects through progressive biological responses that may enhance skin firmness, elasticity, thickness, and overall dermal integrity [60].

Commonly used biomodulators include poly-L-lactic acid, calcium hydroxylapatite, polycaprolactone, polynucleotides, platelet-derived products, and other regenerative agents [58,61,62]. Depending on their composition and biological properties, these materials may promote neocollagenesis, angiogenesis, modulation of inflammatory signaling, and cellular repair [63,64]. Their clinical effects generally develop over time and may be influenced by factors such as product formulation, injection depth, treatment interval, and the regenerative capacity of the treated tissue [65,66].

### 4.2. Skin Boosters

Skin boosters are injectable or bioactive treatments primarily intended to improve overall skin quality rather than produce substantial volumetric correction. They are typically administered into the superficial or mid-dermis, where they are used to enhance hydration, elasticity, smoothness, and skin texture [20]. Hyaluronic acid-based formulations are among the most commonly used skin boosters because of the strong water-binding capacity of hyaluronic acid and its important role in maintaining dermal hydration and viscoelastic properties [67].

Beyond hyaluronic acid, skin booster formulations may also contain amino acids, peptides, vitamins, antioxidants, polynucleotides, and other bioactive compounds [68]. Depending on their composition, these ingredients may contribute to fibroblast activity, extracellular matrix homeostasis, and tissue repair. Clinically, skin booster treatments have been associated with improvements in skin hydration, fine lines, radiance, surface smoothness, and dermal elasticity [69].

### 4.3. Difference Between Fillers, Skin Boosters, and Biomodulators

Conventional dermal fillers are primarily used to restore soft-tissue volume, soften folds, and modify facial contour through space-occupying and structural volumetric effects [70]. Skin boosters, by contrast, are intended mainly to enhance dermal hydration and overall surface quality without producing substantial volumetric correction [23]. Biomodulators differ from both approaches in that their principal effect is based on biological stimulation, particularly the induction of collagen synthesis and extracellular matrix remodeling [28].

Accordingly, Table 1 summarizes these interventions according to their predominant clinical function; the categories should be interpreted as overlapping functional descriptors rather than as mutually exclusive or regulatory classes.

## 5. Major Types of Biomodulators and Skin Boosters

Biomodulators and skin boosters comprise a heterogeneous group of injectable and bioactive preparations with distinct compositions, physical properties, degradation profiles, and intended clinical uses [22,23,28,71]. This section describes the principal product categories and their formulation characteristics, while the biological mechanisms associated with these interventions are discussed separately in Section 6.

### 5.1. Hyaluronic Acid-Based Skin Boosters

Hyaluronic acid-based skin boosters are widely used to improve skin hydration, elasticity, and surface smoothness [20]. Hyaluronic acid is a naturally occurring glycosaminoglycan with a high capacity for water binding and retention, properties that are important for maintaining dermal hydration and viscoelasticity. When administered into the superficial or mid-dermis, hyaluronic acid-based formulations can increase tissue hydration and contribute to improvements in skin mechanical properties [67].

In contrast to conventional hyaluronic acid fillers, which are primarily used for volumetric correction and contour restoration, skin boosters generally contain non-crosslinked or lightly crosslinked hyaluronic acid and are intended mainly to improve skin quality [72]. Their ability to retain water within the dermal matrix may help reduce fine lines, improve skin texture, and enhance skin radiance [69]. In addition to their hydrating effects, these formulations may also support a local dermal environment that favors fibroblast activity and extracellular matrix homeostasis [73].

### 5.2. Polynucleotides and Polydeoxyribonucleotide: Related but Distinct DNA-Derived Preparations

PN and PDRN are related DNA-derived preparations but should not be treated as interchangeable terms [61,74]. They may differ in molecular-size distribution, manufacturing characteristics, formulation, and the mechanistic emphasis attributed to their clinical use; accordingly, evidence is discussed using the terminology reported for each investigated product [74,75].

### 5.3. Poly-L-Lactic Acid

Poly-L-lactic acid is a biodegradable synthetic polymer widely used as a collagen biostimulator in aesthetic medicine. Following injection, poly-L-lactic acid microparticles induce a gradual tissue response that promotes fibroblast activity and neocollagenesis [76]. Unlike conventional volumizing fillers, its clinical effects develop progressively as new collagen is deposited and dermal structure is remodeled over time.

Clinically, poly-L-lactic acid is used for age-related changes that include laxity, dermal thinning, and soft-tissue volume loss [77]. Its use differs substantially according to reconstitution, treatment volume, injection depth, anatomical site, and number of treatment sessions. These technical variables are clinically relevant because inappropriate preparation or uneven placement may contribute to nodules and contour irregularities [78].

### 5.4. Calcium Hydroxylapatite

Calcium hydroxylapatite is a biostimulatory injectable composed of calcium hydroxylapatite microspheres suspended in a gel carrier. The carrier provides an initial volumizing and corrective effect, while the microspheres contribute to a more gradual biological response by supporting fibroblast activity and collagen formation [79]. As the gel carrier is resorbed, newly deposited extracellular matrix and collagen may provide longer-lasting dermal support [80].

Clinically, calcium hydroxylapatite is used both for soft-tissue augmentation and for treatment protocols directed at skin laxity and dermal quality [81]. Its clinical behavior depends partly on dilution and injection technique. Diluted or hyperdiluted preparations are generally used with less emphasis on volumetric correction than undiluted formulations [82]. Direct interaction between CaHA microspheres and fibroblasts has been demonstrated experimentally [83]. The cellular and matrix responses associated with CaHA are discussed in Section 6.

### 5.5. Polycaprolactone-Based Products

Polycaprolactone-based products are biodegradable collagen-stimulating materials used in aesthetic medicine for progressive tissue remodeling [84]. Following injection, polycaprolactone microspheres undergo gradual biodegradation while promoting fibroblast activity and new collagen formation [85]. These biological responses may contribute to sustained improvements in skin firmness, elasticity, and overall tissue structure [86].

Compared with more rapidly resorbed injectable materials, polycaprolactone has a relatively prolonged degradation profile, which may contribute to the durability of its clinical effects [87]. Polycaprolactone-based products are therefore used in facial rejuvenation and soft-tissue support, particularly for the correction of age-related changes in dermal quality and facial structure [88].

### 5.6. Amino Acids, Peptides, Vitamins, and Antioxidants Containing Preparations

Amino acids, peptides, vitamins, and antioxidants are commonly incorporated into skin booster formulations to support dermal metabolism and extracellular matrix homeostasis [19]. Amino acids provide essential substrates for the synthesis of structural proteins such as collagen and elastin, whereas bioactive peptides may modulate cellular signaling pathways involved in fibroblast activity and matrix remodeling [89,90].

Vitamins and antioxidants may further support skin repair and dermal homeostasis by limiting oxidative stress, a key mechanism involved in cutaneous aging [91]. Through the reduction of reactive oxygen species-mediated damage, these components may help preserve cellular function and maintain a more favorable dermal microenvironment [92]. Although they do not primarily provide volumetric correction or direct structural support, they may complement other regenerative treatments by supporting cellular metabolism, hydration, and tissue repair [67].

### 5.7. Autologous Platelet-Derived Preparations: PRP and PRF

PRP and PRF differ conceptually from synthetic injectable materials because they are autologous, protocol-dependent blood-derived preparations whose composition is influenced by platelet concentration, leukocyte content, fibrin architecture, preparation technique, and administration protocol [93]. Accordingly, evidence is evaluated according to the specific preparation and clinical protocol rather than assuming class-wide equivalence.

Platelet-derived preparations provide a heterogeneous mixture of growth factors, cytokines, and other bioactive mediators that may influence fibroblast activity, angiogenic signaling, and tissue repair [94]. However, the clinical evidence should be interpreted cautiously because preparation systems, platelet and leukocyte content, activation methods, injection protocols, and concomitant procedures vary substantially [95]. Findings from a specific PRP or PRF protocol should therefore not be generalized to the entire class. Eligible controlled human studies are summarized separately in the clinical-evidence table.

### 5.8. Extracellular-Vesicle/Exosome-Containing and Secretome-Derived Emerging Preparations

Extracellular-vesicle/exosome-containing and secretome-derived preparations are emerging biologic approaches rather than a standardized clinical product class. Their composition and biological activity may vary according to source material, isolation and purification methods, concentration, storage conditions, and quality-control procedures [96,97].

Preclinical and early clinical studies have reported effects related to fibroblast activity, inflammatory signaling, tissue repair, and post-procedural recovery, particularly in conjunction with laser treatment or microneedling; however, these observations should not be generalized across different preparations or administration routes [98,99]. Regulatory status also varies according to jurisdiction, manufacturing process, intended use, and route of administration [100]. Accordingly, these preparations are discussed as emerging approaches rather than established skin boosters. Regulatory authorization should therefore be assessed at the level of the specific product, jurisdiction, intended use, and administration route; inclusion of a preparation in this review should not be interpreted as evidence of regulatory authorization or endorsement for routine clinical use.

### 5.9. Administration Routes and Exposure Context

Administration routes are considered separately throughout this review. Topical application to intact skin, post-procedural delivery following microneedling or fractional laser treatment, intradermal mesotherapy, and direct injection do not represent equivalent tissue exposures [101,102]. Evidence obtained with barrier-disrupting procedures should therefore not be generalized to direct injection, particularly for extracellular-vesicle-, secretome-, peptide-, and nucleotide-containing preparations [103].

## 6. Mechanisms of Action

The biological responses associated with biomodulators and skin boosters depend on the material, formulation, tissue exposure, and mode of administration. The available mechanistic evidence is derived mainly from cell-based experiments, animal studies, and human histological observations rather than from direct clinical measurements of molecular activity. Reported responses involve fibroblast behavior, collagen and elastin turnover, tissue hydration, vascular signaling, inflammatory pathways, and extracellular-matrix organization [104,105]. These processes are closely related, but they should not be regarded as a single mechanism shared by all products. The mechanical environment itself can alter signaling in aged dermal fibroblasts, including ERK-associated responses, indicating that tissue context may influence how a material-induced stimulus is translated at the cellular level [106]. Hydration-oriented HA preparations, particulate collagen biostimulators, autologous platelet preparations, and EV-containing products are therefore considered separately below.

### 6.1. Fibroblast Activation

Fibroblasts are a major cellular target in dermal remodeling because they regulate the synthesis, organization, and turnover of collagen and other extracellular-matrix components. Several injectable biomaterials have been associated with changes in fibroblast activity, but the stimulus differs according to the product.

Particulate biostimulators interact with surrounding tissue after implantation and can induce a local cellular response that is followed by fibroblast recruitment and matrix deposition. PCL-based collagen stimulators, for example, are designed around biodegradable microspheres that persist temporarily within the tissue while a gradual host response develops [107]. Particle size, surface properties, degradation behavior, tissue distribution, and the accompanying inflammatory reaction can influence the subsequent cellular response.

Polynucleotide-derived preparations involve a different biological input. Experimental studies of PDRN have reported effects on inflammatory signaling and repair-associated cellular responses [108]. Platelet-derived products act through a heterogeneous mixture of growth factors, cytokines, and other mediators rather than through persistent implanted particles [62,94]. The same distinction is relevant to multicomponent skin boosters, in which several constituents may act simultaneously and make it difficult to assign a cellular response to a single ingredient.

### 6.2. Collagen Type I and III Synthesis

Changes in collagen synthesis are among the most frequently cited biological effects of collagen biostimulators. Increased fibroblast activity can alter collagen production and gradually affect dermal matrix density. Experimental work with PCL microspheres has identified increased collagen synthesis together with involvement of the SAMD11–PLOD1 axis [109]. Material-specific tissue responses are also important: comparative preclinical work with CaHA and PLLA has shown differences in macrophage and inflammatory pathway activation, which may influence the subsequent remodeling response [110].

Collagen-related outcomes also need to be interpreted according to the method used to measure them. Increased collagen-gene expression, increased collagen staining, and a measurable increase in dermal thickness represent different levels of observation and should not be treated as interchangeable findings. Histological evidence of new collagen deposition provides stronger structural information than changes in gene expression alone, but neither finding necessarily predicts the magnitude of a visible clinical response.

Type I collagen contributes predominantly to tensile strength, whereas type III collagen is more closely associated with a more flexible matrix and early stages of tissue repair. Changes in their relative abundance may therefore influence dermal organization as well as total collagen content. The gradual nature of this process is consistent with the delayed clinical response observed with particulate biostimulators, in contrast to the immediate physical effects produced by conventional volumizing fillers [55].

### 6.3. Elastin Remodeling

Elastin has received less attention than collagen in studies of injectable rejuvenation treatments. Available reports suggest that some interventions may influence elastin-related pathways or elastic-fiber organization, but the evidence is less consistent than that for collagen [111]. Fibroblast activity and changes in the surrounding extracellular matrix may indirectly affect the maintenance and organization of elastic fibers.

This distinction is important because increased expression of elastin-related genes does not necessarily demonstrate reconstruction of a mature elastic-fiber network. Functional elastic fibers depend on appropriate assembly and organization within the dermal matrix. Clinical changes in elasticity or cutometric measurements therefore cannot be attributed to elastin regeneration alone. Hydration, collagen organization, glycosaminoglycan content, and dermal thickness can also alter measured skin elasticity and viscoelasticity [67].

### 6.4. Angiogenesis and Tissue Repair

Dermal repair requires an adequate microvascular supply. Cutaneous vessels provide oxygen and nutrients and participate in the delivery of signals involved in fibroblast activity, matrix deposition, and wound repair [53]. Age-related impairment of vascular function may therefore reduce the capacity of the dermis to respond to injury or regenerative stimulation.

Polynucleotides, platelet-derived preparations, and secretome-based products have been associated with angiogenic signaling in experimental systems [112,113,114]. Platelet-derived preparations contain multiple mediators released from activated platelets, whereas secretome-based preparations contain a more heterogeneous mixture of soluble factors and, depending on the preparation, extracellular vesicles. These products should therefore not be assumed to produce vascular effects through an identical pathway.

Increased vascular signaling may assist tissue repair by improving the local environment in which fibroblasts, immune cells, and other repair-associated cells function [115]. At the same time, evidence of increased angiogenic markers or vessel density in experimental models should be distinguished from a demonstrated improvement in clinical skin rejuvenation.

### 6.5. Hydration and Dermal Viscoelasticity

Hydration is a central mechanism for HA-based skin boosters and differs conceptually from the delayed matrix response associated with particulate biostimulators. HA has a strong capacity to bind and retain water within the extracellular matrix. Increasing local water content can alter tissue hydration, surface smoothness, and dermal viscoelastic behavior [18,67].

The magnitude and persistence of these effects depend on the characteristics of the HA formulation. Concentration, molecular properties, degree of crosslinking, tissue distribution, and degradation rate can influence how long the material remains in the treated area and how it interacts with water and surrounding tissue. Consequently, improvement in hydration observed with one HA preparation should not be interpreted as a class-wide effect of all HA-based skin boosters.

Hydration may also affect the appearance of fine lines without requiring substantial new collagen formation. This provides one explanation for why early improvements in surface quality can occur on a different time scale from the delayed changes reported after collagen-biostimulatory treatment.

### 6.6. Anti-Inflammatory and Antioxidant Effects

Inflammatory signaling has a complex role in dermal remodeling. Chronic inflammation and oxidative stress contribute to fibroblast dysfunction and matrix degradation in aged skin, whereas a limited local inflammatory response can form part of the tissue reaction to injected biomaterials. The biological meaning of “anti-inflammatory” activity therefore depends on the product and experimental setting.

Polynucleotide-derived preparations, vitamins, peptides, and exosome-related approaches have been reported to influence inflammatory or oxidative-stress pathways [90,92,116,117]. Such effects may reduce cellular stress or support recovery after tissue injury. Particulate biostimulators differ in this respect: comparative preclinical work with CaHA and PLLA has shown material-specific macrophage and inflammatory responses [110]. A controlled local tissue response can therefore form part of biostimulation rather than representing an effect that must always be suppressed.

These differences are particularly relevant when interpreting combination treatments with laser, radiofrequency, or microneedling, because the procedure itself induces a wound-healing response. In these settings, changes in inflammatory markers cannot necessarily be attributed to the accompanying injectable or topical preparation alone [118].

### 6.7. Extracellular Matrix Remodeling

Extracellular-matrix remodeling is better viewed as the result of several interacting processes than as an isolated mechanism. Changes in fibroblast activity, collagen synthesis, elastic-fiber organization, hydration, inflammatory signaling, and tissue repair can all alter the structure and mechanical behavior of the dermis [119].

The pattern of remodeling differs according to the intervention. HA-dominant preparations may change hydration and matrix viscoelasticity relatively early, whereas particulate biostimulators are associated with a slower sequence of tissue response, fibroblast activity, and collagen deposition. PN/PDRN, platelet-derived preparations, and emerging biologic products involve still different combinations of repair-associated signals.

**Figure 2:**
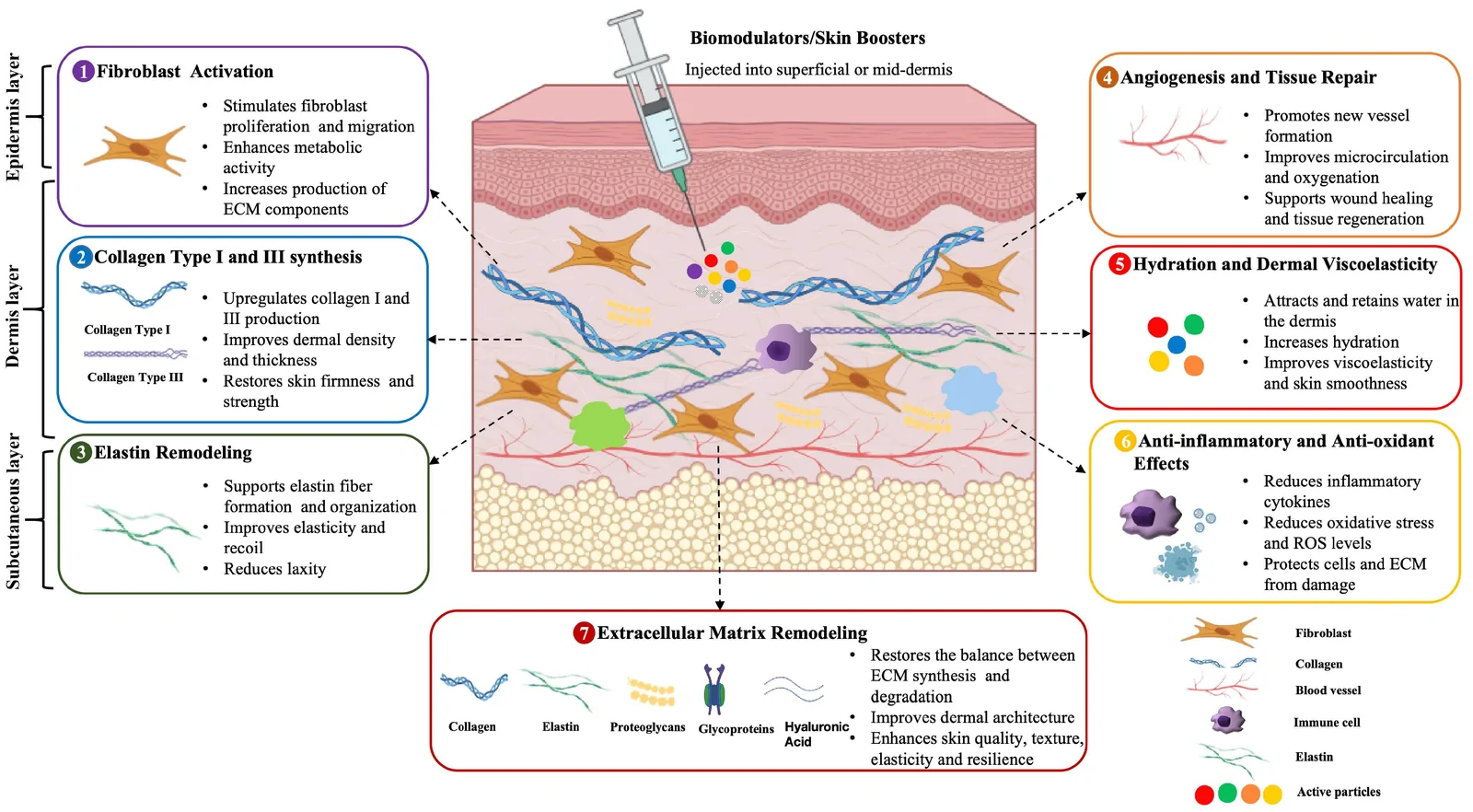
Proposed Mechanisms of Biomodulators and Skin Boosters in Dermal Repair and Remodeling. Created in BioRender. Pham, N. C. (2026) https://BioRender.com/kjaq99m (accessed on 3 October 2026).

For this reason, an increase in dermal thickness, collagen staining, elasticity, or skin density should not automatically be described as equivalent evidence of “regeneration.” These endpoints describe different aspects of tissue response. Their clinical relevance also depends on anatomical site, treatment protocol, follow-up period, baseline skin condition, and the specific formulation used [78,120]. The proposed mechanisms of dermal repair and remodeling are summarized in Figure 2.

## 7. Evidence Synthesis Across Experimental and Clinical Levels

For clarity, mechanistic in vitro evidence, in vivo animal evidence, and human clinical evidence are considered separately because they address different questions and should not be assigned equivalent evidentiary weight [121,122].

### 7.1. In Vitro Mechanistic Evidence

In vitro studies provide a controlled setting in which cellular responses to individual materials or formulations can be examined. Frequently measured endpoints include fibroblast viability, proliferation, migration, collagen-related gene expression, cytokine production, and responses to oxidative stress [123,124,125]. These experiments are particularly useful for identifying candidate pathways, but the exposure conditions used in culture do not reproduce the anatomical, mechanical, vascular, or immune environment of human skin.

The available studies also differ substantially in cell source, passage number, product concentration, exposure duration, and experimental endpoint. HA-based preparations have been examined for effects on fibroblast viability and matrix-related responses, while PN/PDRN studies have reported changes in fibroblast proliferation, collagen-associated genes, and repair-related signaling [126,127,128,129,130,131]. Platelet-derived and secretome-based preparations expose cells to complex mixtures of soluble mediators rather than to a single defined compound [132]. Particulate biostimulators have also produced measurable fibroblast and matrix responses under experimental conditions [83,133,134].

These findings support the biological plausibility of several proposed mechanisms described in Section 6, but they do not establish the magnitude or durability of a treatment effect in patients. Concentrations that produce a measurable cellular response in vitro may not correspond to tissue exposure after clinical administration, and differences between commercial formulations may also alter the response. In vitro evidence is therefore used here to identify mechanistic signals rather than as direct evidence of clinical efficacy.

### 7.2. In Vivo Preclinical Evidence

Animal models extend mechanistic observations from isolated cells to intact tissue, allowing evaluation of inflammatory responses, collagen deposition, dermal thickness, vascular changes, biodegradation, and tissue integration [135,136,137]. These models are particularly useful for materials whose activity depends on interactions among fibroblasts, inflammatory cells, vasculature, and the extracellular matrix.

Preclinical studies have reported changes in dermal hydration or matrix organization after HA-based and other biodegradable filler interventions, repair-associated responses after PN/PDRN administration, and progressive collagen deposition following treatment with particulate biostimulators [86,136,138,139,140,141,142,143,144,145,146].

The translational value of these findings nevertheless depends on the model. Rodent skin differs from human skin in thickness, hair density, wound contraction, immune response, and tissue mechanics. Dose, injection volume relative to tissue size, and follow-up duration may also differ markedly from clinical practice [147,148]. Animal findings therefore provide evidence of tissue-level biological activity, but they should not be used alone to infer the magnitude of cosmetic benefit in human skin.

### 7.3. Human Clinical Evidence

Human studies provide the most direct basis for determining whether the biological responses observed in experimental models translate into measurable changes in human skin. Clinical outcomes reported in this field include skin hydration, elasticity, wrinkle severity, dermal thickness or density, surface texture, patient satisfaction, and treatment-related adverse events [149,150]. Objective assessment methods include high-frequency ultrasound, cutometry, corneometry, profilometry, standardized photography, and histological analysis [151,152,153].

These outcomes reflect different aspects of treatment response and should not be treated as interchangeable. An increase in hydration does not necessarily indicate neocollagenesis, while an increase in ultrasound-measured skin density is not equivalent to a reduction in wrinkle severity or an improvement in patient-reported skin quality. Clinical findings therefore need to be interpreted according to the specific product, treatment protocol, anatomical site, comparator, follow-up period, and outcome measured.

**Figure 3:**
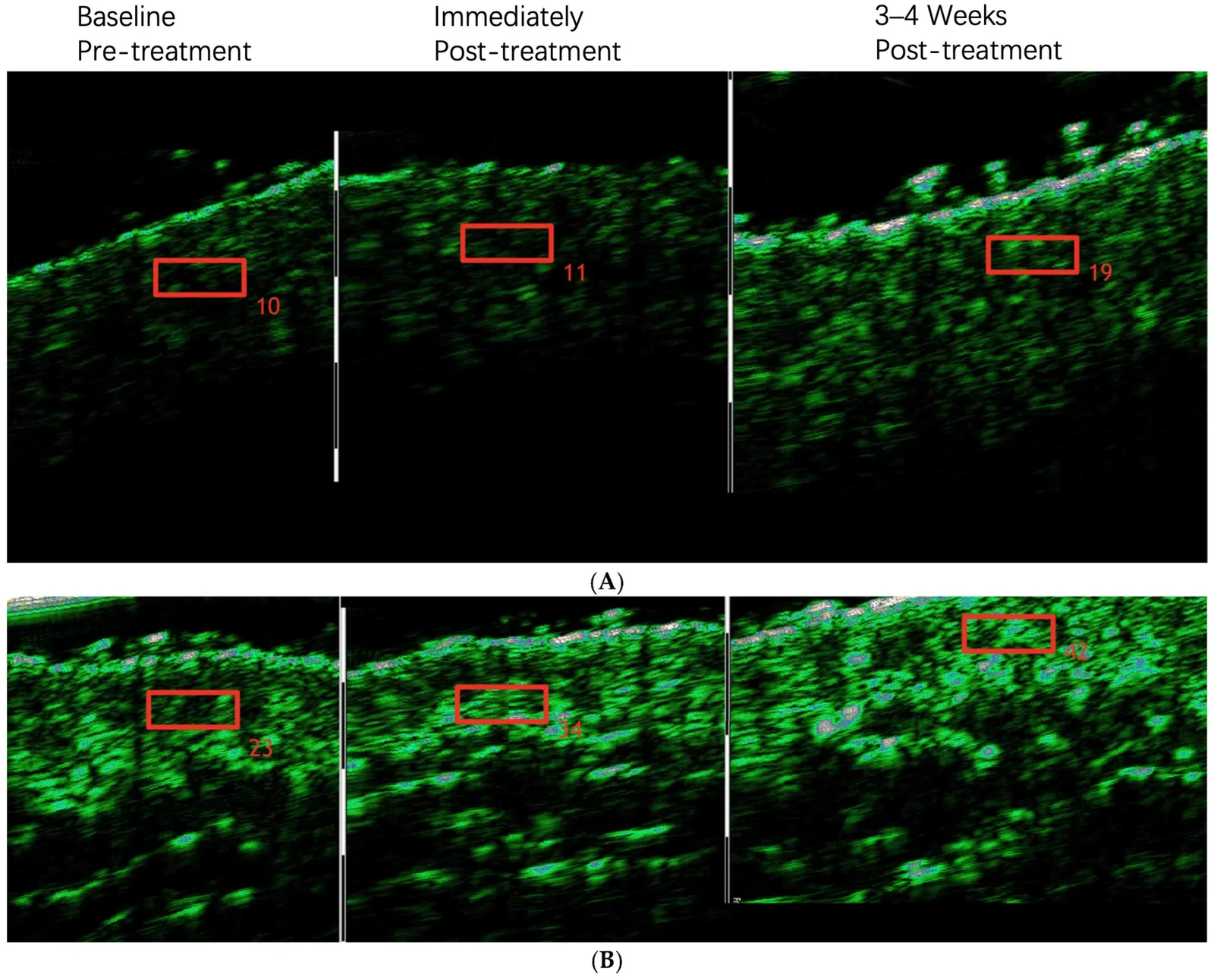
Representative high-frequency ultrasound images showing cheek skin-density measurements following a single cannula-delivered midface treatment with a non-stabilized hyaluronic-acid-based bioactive hydrogel (TEOSYAL^®^ Redensity [I]). For each patient, images are shown at baseline (pre-treatment), immediately after treatment, and 3–4 weeks after treatment. (**A**) Patient 1; (**B**) Patient 2. The red rectangles indicate the measurement regions, and the adjacent numbers represent the corresponding ultrasound-derived skin density values. Reprinted under the terms of the Creative Commons Attribution (CC BY 4.0) license from Ref. [154].

Within the contextual non-randomized evidence stream, a retrospective controlled study of a multicomponent HA-based hydrogel reported an increase in ultrasound-measured facial skin density 3–4 weeks after treatment compared with an untreated control region [154]. This study provides imaging evidence of a measurable tissue change, although its retrospective design and short follow-up limit causal interpretation. Representative ultrasound images are shown in Figure 3.

The maturity of human clinical evidence differs across intervention groups. HA-based injectable treatments and particulate collagen biostimulators have been evaluated in a number of controlled human studies, whereas evidence for PN/PDRN and platelet-derived preparations remains more heterogeneous in formulation, treatment protocol, and clinical indication [155]. EV/exosome-containing and secretome-derived preparations are supported mainly by mechanistic, preclinical, and early or procedure-assisted clinical studies. Differences in source material, manufacturing, characterization, and route of administration further limit comparison across these preparations [156,157].

Human studies also differ substantially in formulation, comparator, anatomical site, treatment schedule, follow-up duration, and outcome assessment [158,159]. Controlled comparative studies provide more direct evidence of treatment effect than uncontrolled observational studies, whereas in vitro and animal studies are primarily informative about biological plausibility [160,161]. For this reason, the clinical evidence below is discussed at the level of the investigated product and treatment protocol rather than as evidence for uniform class effects.

#### 7.3.1. HA-Based and Multicomponent HA Preparations

HA-based preparations have the broadest randomized clinical evidence among the skin-booster interventions identified in this review. The included randomized studies examined a sodium-hyaluronate composite solution, cohesive polydensified matrix HA, HA treatment for neck wrinkles, VYC-12L for cheek skin quality, and fractional-laser-assisted HA delivery [162,163,164,165,166]. Other randomized HA studies already cited earlier in the review evaluated an HA micro-filler and non-crosslinked HA in controlled or split-face protocols [69,72].

The studies differed in formulation, treatment volume, injection depth, number of sessions, anatomical site, comparator, and follow-up period. Several reported improvements in selected outcomes such as hydration, elasticity, superficial wrinkles, skin roughness, or patient-reported skin quality. However, these trials did not evaluate a single standardized HA intervention. The available evidence therefore supports the specific HA formulations and protocols that were tested rather than a uniform clinical effect of all HA-based skin boosters.

The distinction between hydration-related effects and structural remodeling is also important when interpreting these studies. Some HA formulations may produce relatively early changes in hydration, smoothness, or viscoelasticity, whereas evidence of changes in dermal structure depends on the formulation, measurement method, and duration of follow-up. A clinical improvement in fine lines or surface texture should therefore not automatically be attributed to new collagen formation.

#### 7.3.2. Particulate Collagen Biostimulators

PLLA studies included protocols involving microneedling-radiofrequency-assisted delivery, midface rejuvenation, different reconstitution approaches, and arm contouring [167,168,169,170]. Additional PLLA studies cited earlier in the review evaluated patient satisfaction and histological outcomes [55,77].

The clinical endpoints varied across these studies and included laxity, wrinkles, three-dimensional changes, patient satisfaction, dermal remodeling, and treatment durability. The findings support activity of the investigated PLLA protocols, but the studies differed substantially in anatomical site, preparation, injection technique, treatment schedule, and follow-up. These differences make direct comparison between studies difficult and limit broader conclusions about PLLA as a single uniform intervention.

Randomized studies of CaHA evaluated diluted CaHA for décolletage wrinkles, CaHA combined with a polymicronutrient solution for hand rejuvenation, hybrid CaHA-HA formulations, and diluted CaHA-CMC treatment [66,171,172,173]. Reported outcomes included wrinkles, hydration, skin quality, firmness, and other measures of tissue response. Interpretation of CaHA studies requires particular attention to dilution and formulation because these variables influence the balance between immediate structural effects and the more gradual tissue response associated with collagen stimulation.

Human evidence for PCL in the present dataset was more dependent on non-randomized clinical studies than the evidence available for PLLA or CaHA. Clinical observations support its use as a collagen-stimulating material, but the smaller controlled evidence base limits direct comparison with the more extensively studied particulate agents. PLLA, CaHA, and PCL should therefore remain separated at the level of clinical interpretation rather than being treated as a single “collagen biostimulator” intervention.

#### 7.3.3. Platelet-Derived and Nucleotide-Derived Preparations

Platelet-derived preparations were represented by several randomized studies evaluating hand rejuvenation, neck wrinkles, and atrophic acne scars [174,175,176,177]. The investigated protocols differed considerably in PRP preparation, concomitant procedures, anatomical indication, and outcome assessment. Some studies evaluated PRP alone, whereas others combined platelet-derived preparations with microneedling, radiofrequency, or other regenerative approaches.

The results were not uniformly positive. In the randomized split-hand study by Pincelli et al., PRP did not show a significant advantage over saline for several physician-assessed, photographic, and patient-reported outcomes [62]. This negative finding is important because it shows that biological plausibility and growth-factor content do not necessarily translate into measurable clinical superiority under every treatment protocol.

Variation in platelet concentration, leukocyte content, activation method, preparation system, injection protocol, and concomitant procedure further complicates interpretation. A clinical response observed with one PRP preparation cannot therefore be assumed to apply to all platelet-derived products.

Randomized evidence for nucleotide-derived preparations was more limited. One placebo-controlled study evaluated highly purified PN in patients with moderate-to-severe atrophic acne scars [178]. This provides controlled clinical evidence for the investigated PN protocol in that indication, but atrophic acne scarring is biologically and clinically distinct from chronological or photoinduced skin aging. The result should therefore not be generalized to facial rejuvenation more broadly or to other PN/PDRN formulations.

PN and PDRN should also remain separated when interpreting clinical evidence. Although both are DNA-derived preparations, they differ in molecular characteristics, formulation, and the mechanistic emphasis attributed to their use. Evidence obtained with one preparation should not be transferred automatically to the other.

#### 7.3.4. EV/Exosome-Containing and Secretome-Derived Preparations

Three randomized reports evaluated EV/exosome-containing or secretome-derived preparations within procedure-assisted treatment protocols [99,118,179]. These preparations should be considered separately because they differ in source material, composition, characterization, and delivery method.

Park et al. compared an adipose-derived stem-cell exosome-containing solution plus microneedling with saline plus microneedling [99]. This design can assess the incremental effect of the complete preparation within the microneedling protocol, but it cannot isolate the contribution of its exosome component. Yusharyahya et al. compared microneedling and fractional CO_2_ laser as delivery methods for the same secretome preparation [118]. This study therefore informs the relative performance of the investigated delivery protocols rather than the benefit of adding secretome to a procedure. Huang et al. evaluated needling radiofrequency with or without a plant-derived exosome-based formulation, providing preliminary comparative evidence within that specific procedural setting [179].

These findings do not establish the efficacy of the preparations as standalone treatments or support direct injectable exosome therapy. Nor should they be generalized across EV-containing, plant-derived, and secretome-derived products. Differences in manufacturing, purification, characterization, concentration, storage, and administration route further limit extrapolation between preparations [156,157]. The available evidence remains preliminary and protocol-specific, and larger controlled studies with clearly defined products and longer safety follow-up are needed.

#### 7.3.5. Overall Interpretation of the Human Evidence

The retained comparative reports do not represent a homogeneous treatment group. Product composition, comparator, anatomical site, treatment schedule, follow-up period, and outcome measurement differed substantially between reports [158,159]. These differences are the main reason why a pooled cross-category estimate was not considered appropriate.

Contextual and noncomparative reports add clinically useful information on durability, feasibility, histological changes, imaging findings, treatment patterns, and longer-term observations. Their designs remain more vulnerable to selection bias, confounding, lack of blinding, and absence of an adequate comparator. These findings were therefore used as context rather than as the primary basis for causal efficacy conclusions.

Examples of the contextual clinical evidence include longer-term follow-up of HA filler treatment for atrophic facial scars and a prospective study of HA filler for horizontal neck lines [180,181]. Other investigations examined crosslinked HA for facial volume restoration and sonographic tissue thickness, roughness, and hydration after dorsal-hand injectable treatment [182,183]. Product-specific reports also evaluated PLLA for facial rejuvenation, hyperdilute CaHA for perioral rhytids, and a premixed CaHA/CMC-HA hybrid filler for skin quality [184,185,186]. A split-face study compared fractional CO_2_ laser combined with autologous nanofat or PRP for atrophic acne scars, while observational clinical data addressed injectable PN-HPT for skin rejuvenation [187,188]. Adipose-derived signaling nanovesicle preparations were examined in an antiaging clinical report, and exosomes were compared with PRP within radiofrequency microneedling protocols for photoaged facial skin [189,190]. These reports broaden the clinical context but concern different formulations, indications, delivery routes, and outcome measures. Longer-term follow-up reports should be distinguished from the randomized comparisons that may precede them; volumetric outcomes do not by themselves demonstrate dermal regeneration, and combination-treatment findings do not establish the independent contribution of each component.

The three evidence levels considered in this review answer different questions. Mechanistic studies can identify cellular or molecular responses that may explain how a treatment acts. Animal and other preclinical models can show how these responses develop within intact tissue. Controlled human studies are required to determine whether the intervention produces a measurable clinical effect under a defined treatment protocol [160,161]. Evidence at one level cannot substitute for evidence at another.

This distinction is particularly important in a field in which similar clinical terms are applied to products with very different compositions and modes of administration. The current evidence does not support treating “skin boosters,” “biomodulators,” or “regenerative injectables” as clinically uniform categories. Claims of efficacy should remain linked to the specific formulation, route of administration, indication, and outcome that were actually investigated.

All identified comparative human reports meeting the stated eligibility criteria were retained irrespective of the direction or statistical significance of their findings. Contextual human reports, noncomparative primary estimates, and unresolved reports were retained in separate categories.

Several non-randomized reports evaluated sequential or combination treatment protocols, including CaHA followed by HA administration and platelet-derived or EV-containing preparations combined with energy-based procedures. These studies provide clinically relevant contextual observations; however, their findings cannot necessarily be attributed to individual treatment components. Similarly, studies primarily investigating volumetric correction should not be interpreted as direct evidence of skin-quality improvement unless appropriate skin-quality outcomes were assessed. Selected comparative human clinical evidence is summarized in Table 2.

### 7.4. Risk-of-Bias Assessment of Comparative Human Evidence

Risk-of-bias assessments were completed for 159 specified comparative results. Incomplete reporting was treated as no information rather than as evidence that the underlying study procedures were adequate.

Overall, 123 specified results were judged High and 36 raised Some concerns; none was Low across all five domains. Domain totals were as follows: randomization process, 21 Low and 138 Some concerns; deviations from assignment, 72 Low and 87 Some concerns; missing outcome data, 47 Low and 112 High; outcome measurement, 100 Low, 41 Some concerns, and 18 High; and selection of the reported result, one Low and 158 Some concerns. These counts describe result-specific methodological concerns and do not represent efficacy or certainty grades.

The implications depend on the outcome assessed. Uncertainty regarding randomization or allocation concealment weakens confidence that treatment comparisons were protected from selection bias. Lack of participant blinding may influence satisfaction and other patient-reported outcomes, whereas lack of assessor blinding may affect clinical ratings. Instrumental measurements can reduce some forms of subjectivity but do not eliminate bias arising from study conduct, missing data, or selective reporting. Insufficient information about prespecified outcomes also limits assessment of whether the reported results were selected from multiple measurements or analyses.

Accordingly, the presence or number of comparative reports should not be interpreted as establishing high certainty of benefit. Clinical conclusions must also consider risk of bias, sample size, consistency, comparator, follow-up, and relevance to the specific product and indication. Contextual and noncomparative reports did not resolve limitations in the comparative efficacy evidence.

**Figure 4:**
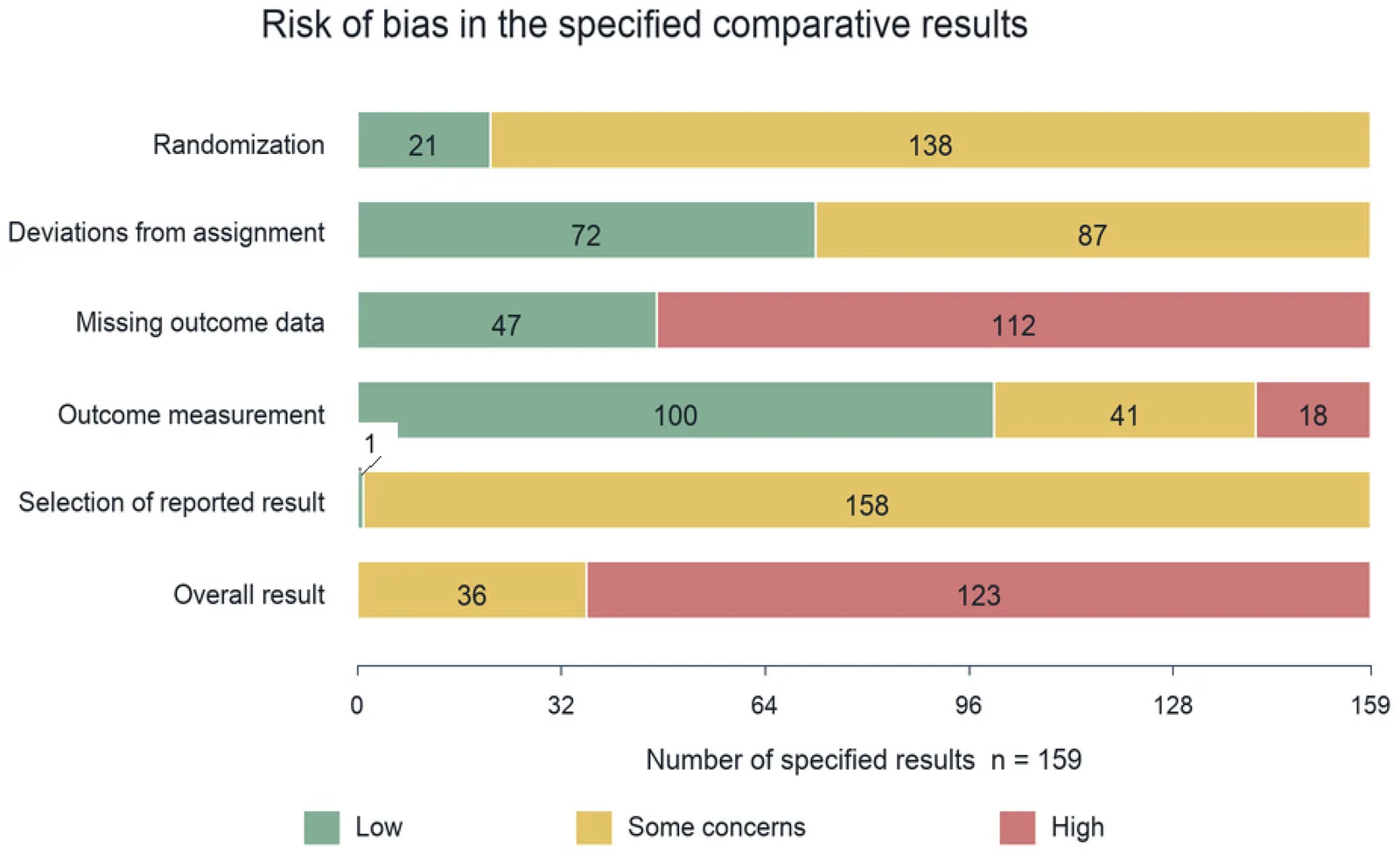
RoB 2 judgments for 159 specified comparative results. Each domain contains 159 judgments. Overall judgments were High for 123 results and Some concerns for 36; none was Low. Domain totals were: randomization process, 21 Low and 138 Some concerns; deviations from assignment, 72 Low and 87 Some concerns; missing outcome data, 47 Low and 112 High; outcome measurement, 100 Low, 41 Some concerns, and 18 High; and selection of the reported result, one Low and 158 Some concerns. Counts are result-specific methodological appraisals and are not certainty grades.

Figure 4 provides a visual summary of the domain-level and overall RoB 2 judgments for the 159 specified comparative results. The distribution describes methodological concerns in the assessed results and does not compare treatment efficacy or establish certainty of benefit.

## 8. Clinical Applications

### 8.1. Facial Rejuvenation

Facial skin rejuvenation is the anatomical setting with the largest body of clinical literature among the indications considered in this review [191,192]. However, the available studies evaluate different products and different endpoints and should not be interpreted as evidence for a uniform class effect [193]. Controlled studies have reported improvements in selected facial skin-quality outcomes with specific HA-based formulations, while the available PLLA evidence concerns formulation-specific treatment of the midface [194]. Evidence for PN/PDRN, PCL, platelet-derived products, and EV/secretome-containing preparations is less consistent in design, indication, or level of control [195]. Treatment effects should therefore be interpreted according to the investigated product, anatomical site, administration protocol, comparator, and follow-up period rather than extrapolated to biomodulators or skin boosters as broad categories [196].

Commonly treated areas include the cheeks, temples, perioral region, jawline, and lower face [197,198,199]. In these areas, biomodulators may help improve tissue support and facial contour, whereas skin boosters are generally used to address fine lines, dullness, and dehydration [200,201]. The choice of treatment should be individualized according to skin thickness, the degree of laxity, underlying volume deficiency, and patient expectations [202,203].

### 8.2. Neck and Décolletage Rejuvenation

The neck and décolletage are particularly vulnerable to photoaging and commonly develop dermal thinning, fine wrinkling, laxity, dryness, and uneven skin texture [173,204]. The relatively thin skin and lower density of sebaceous glands in these areas may contribute to reduced hydration and the early appearance of visible aging [205]. Skin boosters are primarily used to improve hydration, elasticity, and superficial crepiness, whereas diluted or hyperdiluted biostimulatory injectables may enhance dermal firmness and increase collagen density [206].

Because the skin in these regions is thin and anatomically sensitive, careful injection technique is essential to minimize visible product irregularities [207]. Small-volume injections distributed evenly across the treatment area are generally preferred to reduce the risk of nodularity, edema, and uneven surface texture [208]. Multiple treatment sessions may be required to achieve gradual and natural-looking improvement [164].

### 8.3. Hand Rejuvenation

Hand aging involves both cutaneous changes and loss of subcutaneous volume, and evidence from volumetric correction should therefore be distinguished from evidence for skin-quality improvement [209,210]. Human evidence for biomodulatory treatment of the dorsal hand remains limited and product-specific [211]. Importantly, the randomized split-hand study of autologous PRP included in the present synthesis did not identify a significant advantage over saline in physician assessment, blinded photographic evaluation, or patient-reported outcomes at up to 24 weeks [62]. Findings from other injectable materials should therefore not be extrapolated to platelet-derived preparations or interpreted as evidence of a general biomodulator effect [174]. The limited soft-tissue coverage of the dorsal hand and the superficial location of veins and tendons remain important technical considerations when injectable products are used [212].

### 8.4. Acne Scar Improvement

Atrophic acne scars represent a distinct clinical indication and should not be treated as interchangeable with chronological or photoinduced skin aging [104,213]. The retained randomized PN-HPT report evaluated this setting, while additional PN-HPT reports provide non-randomized contextual observations [178,214,215]. These findings should not be generalized to age-related facial rejuvenation, all PN products, or PDRN [75,216]. Evidence for other injectable or procedure-assisted approaches to acne scars likewise varies according to scar type, concomitant procedures, and treatment protocol [217].

### 8.5. Skin Laxity and Fine Wrinkles

Skin laxity and fine wrinkling are common indications for biomodulators and skin boosters [165]. Laxity is largely related to the progressive degradation of collagen and elastin, reduced dermal thickness, and disruption of extracellular matrix organization [218]. Fine wrinkles are additionally influenced by loss of hydration, reduced elasticity, and age-related changes in the superficial dermis [219].

Specific HA formulations have been investigated for fine lines, hydration, and surface quality, while selected PLLA and CaHA protocols have been evaluated for laxity or tissue-related outcomes [165,170,220]. Evidence for PCL and nucleotide-derived preparations is more limited in the present evidence set and does not support a comparable category-wide conclusion [221,222]. Changes in firmness, ultrasound-measured density, or elasticity are not direct proof of new collagen formation. Clinical responses may vary with baseline skin condition, anatomical site, age, and the complete treatment protocol [223,224].

### 8.6. Combination with Lasers, RF, Microneedling, or Fillers

Combination approaches are increasingly used in skin rejuvenation because individual treatment modalities address different aspects of cutaneous aging [225,226]. Energy-based devices, including fractional lasers and radiofrequency systems, create controlled thermal or mechanical injury within the dermis and initiate subsequent wound-healing responses [227,228]. Microneedling produces microscopic channels in the skin and similarly promotes collagen remodeling [229,230]. When combined with biomodulators or skin boosters, these procedures may provide complementary effects on dermal remodeling, hydration, and extracellular matrix repair [166,167].

Combination-treatment studies require interpretation according to the comparison actually performed [231,232]. When the same procedure is administered in both groups, a comparison with and without the preparation can estimate the incremental effect of the complete added formulation. It does not isolate one constituent of a multicomponent preparation. When both groups receive the preparation but undergo different procedures, the comparison addresses delivery protocols rather than the added preparation. Uncontrolled combined treatment cannot separate component effects.

These distinctions apply to EV/secretome preparations, platelet-derived and nucleotide-derived products, HA boosters, and particulate materials [65,99,118,233]. Findings should therefore remain linked to the complete treatment protocol rather than being presented as evidence for the independent efficacy of each component.

## 9. Safety and Adverse Effects

The safety of biomodulators and skin boosters is influenced by multiple factors, including product composition, injection technique, treatment area, patient characteristics, and post-procedural care [234]. Most adverse events are mild and self-limiting; however, serious complications may occur when products are injected into an inappropriate tissue plane or used in anatomically high-risk regions [235]. Careful patient selection, detailed knowledge of regional anatomy, strict aseptic technique, and appropriate product preparation are therefore fundamental to safe treatment.

Common injection-site reactions include edema, bruising, erythema, tenderness, pain, pruritus, and transient induration [235]. These effects are generally related to needle or cannula trauma and usually resolve within several days without intervention [236,237]. Local swelling and discomfort may be more pronounced with highly hydrophilic formulations, particularly hyaluronic acid-based products, because of their capacity to attract and retain water [238].

Nodules and palpable irregularities have been reported after the use of collagen-stimulating injectables, including poly-L-lactic acid, calcium hydroxylapatite, and polycaprolactone-based products [239]. Their development may be related to overly superficial placement, high product concentration, inadequate dilution, uneven distribution, or failure to follow product-specific aftercare instructions [240,241]. Delayed inflammatory nodules and granulomatous reactions are less frequent but may be more difficult to manage. Depending on the underlying cause, treatment may include anti-inflammatory agents, intralesional corticosteroids, antibiotics, or other product-specific interventions [241].

Vascular compromise is the most serious injection-related complication [242]. Intravascular injection or external vascular compression may result in tissue ischemia, skin necrosis, visual impairment, or blindness [243]. Although these events are uncommon, early recognition and immediate management are essential [244]. Anatomically high-risk regions include the glabella, nose, forehead, nasolabial fold, temple, and periorbital area [245,246]. Preventive measures include slow injection, low injection pressure, the use of small aliquots, appropriate selection of needles or cannulas, and a thorough understanding of vascular anatomy [247].

Hypersensitivity reactions may occur with any injectable formulation and may present as localized edema, erythema, pruritus, delayed inflammation, or, rarely, a systemic allergic response [248]. The risk may be influenced by the biological origin of the product, residual impurities, crosslinking agents, carrier materials, preservatives, and individual immune susceptibility [249]. A detailed medical history should therefore be obtained before treatment, with particular attention to previous reactions to injectable products, allergies, autoimmune disorders, recent infections, and recent vaccination [250].

Infectious complications may arise from inadequate skin preparation, contaminated handling, injection through inflamed or compromised skin, or post-procedural contamination [251]. Persistent pain, erythema, warmth, swelling, discharge, and abscess formation should raise suspicion of infection [252]. Biofilm-related infection should also be considered in patients who develop recurrent or delayed inflammatory nodules [253]. Appropriate skin disinfection, sterile handling, and avoidance of treatment in areas affected by active acne, dermatitis, or infection are important preventive measures [254].

Safety considerations also differ according to product type [23]. Hyaluronic acid-based boosters may produce edema, excessive tissue hydration, or a Tyndall effect when placed too superficially [255]. Poly-L-lactic acid requires appropriate reconstitution, dilution, injection depth, and product distribution to reduce the risk of nodule formation [256]. Calcium hydroxylapatite and polycaprolactone cannot be dissolved with hyaluronidase and therefore require particularly cautious placement [257]. Polynucleotides and polydeoxyribonucleotides are generally used to support tissue repair and improve skin quality, although differences in formulation and biological source should be considered [74,75]. Greater caution is required with exosome- and secretome-based products because manufacturing methods, purity, biological potency, storage stability, and long-term safety remain insufficiently standardized [258].

Injection technique remains one of the most important determinants of both safety and treatment outcome [259]. The injector should understand regional vascular anatomy, tissue thickness, and the appropriate injection plane for each product [260]. Superficial placement of biostimulatory products may increase the likelihood of nodules and visible irregularities, whereas inappropriate or excessively deep placement of skin boosters may reduce their intended effect [23,261]. Product volume, dilution, injection spacing, and treatment interval should therefore be adapted to the anatomical site, tissue characteristics, and clinical indication [169].

Product selection should also be individualized according to age, skin thickness, hydration status, treatment area, degree of laxity, volume deficiency, and the desired outcome [262]. Patients with active inflammation, autoimmune disease, bleeding disorders, very thin skin, unrealistic expectations, or a history of adverse reactions require more careful assessment [263,264]. Multimodal treatment plans should be designed cautiously to avoid excessive inflammation, overlapping tissue injury, and poorly understood interactions between products and procedures [265].

The absence of serious adverse events in a small or short study does not establish long-term safety. Available safety information varies according to formulation, anatomical site, administration route, accompanying procedures, and surveillance period [26,28]. Delayed reactions and repeated-exposure risks remain insufficiently characterized for several emerging preparations, particularly EV/exosome-containing and secretome-derived products [27,157,266]. Larger product-specific cohorts, systematic adverse-event reporting, and extended follow-up are needed to characterize uncommon or delayed harms [267]. Safety findings from topical post-procedural use should not be extrapolated to direct injection.

## 10. Current Challenges

Despite the growing use of biomodulators and skin boosters in aesthetic and regenerative dermatology, several important challenges remain [268]. These include inconsistent terminology, substantial variation in product composition, limited clinical evidence, nonstandardized treatment protocols, heterogeneous outcome measures, and differences in regulatory oversight [269,270,271,272]. Addressing these limitations will be essential to improve the reproducibility, safety, and scientific rigor of future clinical practice and research [273].

### 10.1. Lack of Standardized Terminology

A major limitation in this field is the absence of standardized terminology. Terms such as biomodulators, biostimulators, skin boosters, regenerative injectables, biorevitalizers, and tissue stimulators are often used interchangeably, despite substantial differences in product composition and mechanism of action. Consequently, products with distinct biological effects may be placed within the same category, whereas closely related products may be described using different terms [274].

This lack of consistency complicates both clinical communication and interpretation of the scientific literature [275]. It also limits direct comparison across studies, particularly when product classification and treatment objectives are not clearly defined [58]. More precise terminology is therefore needed to distinguish products that primarily provide hydration, restore volume, stimulate collagen production, promote tissue repair, or modulate cellular signaling [276,277]. Establishing standardized definitions would improve communication among clinicians, researchers, manufacturers, and regulatory authorities and would support more consistent clinical evaluation and reporting [278].

### 10.2. Differences in Formulation and Concentration

Biomodulators and skin boosters show substantial variation in composition, concentration, particle size, degree of crosslinking, molecular weight, carrier system, and bioactive content. As a result, products within the same broad category may produce different biological and clinical effects depending on their specific formulation [68].

Hyaluronic acid-based products, for example, may differ in concentration, crosslinking density, rheological properties, and tissue integration. Polynucleotide formulations vary in molecular size, purity, concentration, and biological source [61]. Exosome- and secretome-based products may also differ according to the source cell, isolation and purification methods, storage conditions, and the composition and concentration of active molecules [30].

Such variability limits the extent to which findings from one product can be applied to another. Clinical efficacy and safety should therefore be evaluated on a product-specific basis rather than assuming equivalence among products classified within the same category [65].

### 10.3. Limited Long-Term Comparative Studies

Although many studies have reported favorable clinical outcomes, robust comparative and long-term evidence remains limited [279]. Much of the available literature is based on small study populations, relatively short follow-up periods, heterogeneous treatment protocols, and inconsistent outcome measures [280]. Consequently, the durability of treatment effects and the long-term safety of many products have not yet been clearly established [60].

Head-to-head comparisons among different biomodulators, skin boosters, and combination strategies are also scarce [281,282]. This limits the ability to determine which approach is most appropriate for specific indications, skin types, age groups, or anatomical regions [158]. Larger, well-designed randomized controlled trials with standardized protocols and extended follow-up are therefore needed to strengthen the current evidence base [18].

### 10.4. Variable Injection Protocols

Injection protocols vary considerably across clinicians and studies. Key variables include injection depth and volume, spacing between injection points, number of treatment sessions, treatment interval, dilution ratio, choice of needle or cannula, and post-procedural care [18]. Differences in these parameters may substantially affect both clinical efficacy and the risk of adverse events [60].

For collagen biostimulators, inadequate dilution, superficial placement, or uneven product distribution may increase the likelihood of nodules and contour irregularities [283]. In the case of skin boosters, excessively superficial injection may result in visible papules or a Tyndall effect, whereas overly deep placement may reduce the intended hydration-related benefits [20,284]. Standardized protocols should therefore be established according to product characteristics, anatomical site, and clinical indication [285].

### 10.5. Need for Objective Evaluation Methods

Assessment of treatment outcomes commonly relies on standardized clinical photography, physician-based evaluation, and patient-reported satisfaction [286]. Although these approaches are clinically useful, they remain partly subjective and may be affected by variations in lighting, camera settings, patient expectations, and evaluator interpretation [287].

The incorporation of objective assessment tools may improve the accuracy and reproducibility of clinical studies [288]. Commonly used methods include corneometry for skin hydration, cutometry for elasticity, high-frequency ultrasound for dermal thickness, three-dimensional imaging for changes in facial contour, and profilometry for wrinkle analysis [168,289,290,291]. Histological and molecular assessments may provide additional information on collagen production and extracellular matrix remodeling [292]. Greater standardization of outcome measures would facilitate comparisons across studies and allow more reliable evaluation of treatment efficacy [293].

### 10.6. Regulatory and Safety Concerns for Emerging Products

Regulatory and safety considerations are particularly relevant to emerging biologic preparations, including extracellular-vesicle/exosome-containing preparations, secretome-derived formulations, stem-cell-derived products, and complex bioactive preparations [97,294]. These products may contain multiple biologically active components, and their effects can vary substantially according to the source material, manufacturing process, purification method, storage conditions, and quality-control standards [295].

The absence of standardized production and characterization methods creates uncertainty regarding product purity, potency, sterility, stability, and long-term safety [296]. There is also concern that some products may enter clinical use before sufficient evidence has been established [297]. More rigorous regulatory oversight, transparent reporting of product composition and manufacturing methods, and well-designed safety studies are therefore needed before these emerging therapies can be widely incorporated into routine clinical practice [298].

### 10.7. Limitations of the Present Review

Several limitations of the present review should be acknowledged. First, the reproducible update completed on 16 October 2026 searched PubMed/MEDLINE systematically but did not search Embase, Scopus, Web of Science, trial registries, or unpublished literature comprehensively. Relevant records available only through those sources may therefore have been missed. Mechanistic and preclinical literature was selected narratively to provide biological context rather than through a separate systematic search.

Second, publication bias and selective outcome reporting cannot be excluded. Studies reporting favorable or statistically significant findings may be more likely to be published than studies reporting negative or inconclusive results. Substantial heterogeneity in interventions, comparators, and outcome measures precluded quantitative pooling and a formal publication-bias assessment.

Third, differences in product formulation, anatomical indication, administration route, treatment protocol, comparator, follow-up duration, and outcome assessment limited direct comparisons across intervention categories. One result was specified retrospectively for each comparative report, and no GRADE assessment was performed. These features restrict the certainty and generalizability of the findings. Because the single outcome and assessment time were selected retrospectively rather than prospectively prespecified, selective-result bias cannot be excluded particularly when the underlying report included multiple eligible outcomes or time points. Together with single-database retrieval and the predominance of High overall RoB 2 judgments (123 of 159 specified results), this limits confidence in apparent benefits and precludes strong comparative efficacy claims.

Finally, small sample sizes, relatively short follow-up periods, and inconsistent adverse-event reporting limited conclusions regarding treatment durability and long-term safety. These limitations are particularly important for emerging EV/exosome-containing and secretome-derived preparations, for which delayed adverse reactions, repeated-exposure risks, and long-term clinical outcomes remain insufficiently characterized. Findings from procedure-assisted administration cannot be generalized to direct injectable preparations without additional evidence.

Study selection was not independently assessed by two reviewers. The unavailable historical search was not reconstructed. The independently dated update provides a reproducible audit trail, but four reports remained access-unresolved and eight retrieved reports remained unresolved with respect to design or eligibility. These constraints, together with the use of report-level rather than independent-trial counts, limit claims of completeness and certainty. Moreover, historical records not freshly reassessed during the update retained their prior classifications unless subsequent source adjudication justified a change. Thus, the documented update improves traceability but cannot establish exhaustive, independently replicated selection across the combined historical and updated evidence inventory.

## 11. Future Perspectives

The future development of biomodulators and skin boosters is likely to move toward more personalized, regenerative, and evidence-based approaches [299]. Skin aging varies considerably according to age, genetic background, skin type, hormonal status, lifestyle, anatomical region, and environmental exposure, making a uniform treatment strategy unlikely to be suitable for all patients [300,301,302]. Personalized regenerative aesthetics will therefore require product selection and treatment planning based on individual skin quality, collagen loss, hydration status, degree of laxity, inflammatory state, and desired clinical outcome [303].

Combination treatment is also expected to play an increasingly important role [304]. Cutaneous aging involves multiple structural and functional changes, including volume loss, dermal thinning, dehydration, collagen degradation, elastin dysfunction, pigmentary alteration, and surface irregularity [305]. Because these changes arise through different biological mechanisms, a single treatment modality may not provide adequate correction [306]. Biomodulators and skin boosters may therefore be combined with fillers, lasers, radiofrequency, ultrasound, microneedling, platelet-derived preparations, or topical regenerative agents to address several components of aging simultaneously [175,307,308]. However, the optimal treatment sequence, interval, dose, and safety profile of these combinations remain to be established [309].

Advances in delivery technology may further improve the efficacy and safety of regenerative aesthetic treatments [310]. Microneedle systems, controlled-release biomaterials, nanoparticles, hydrogels, and transdermal delivery platforms may permit more precise and sustained delivery of active substances into the dermis [311,312]. Such approaches could reduce the need for repeated injections, improve patient comfort, and prolong biological activity by maintaining therapeutic concentrations within the target tissue [313].

EV/exosome-containing and secretome-derived preparations remain investigational approaches whose translation depends on reproducible product identity, composition, source material, isolation and purification methods, potency, sterility, storage, and batch-release criteria [314,315,316,317]. Manufacturing consistency must be demonstrated at the intended clinical scale. Product- and route-specific regulatory assessment, appropriately controlled human studies, and long-term surveillance are necessary before experimental findings can support broader clinical claims [318].

Biomaterial-based skin boosters may also become increasingly relevant [319]. Injectable hydrogels, biodegradable polymers, extracellular matrix-mimetic materials, and hybrid formulations can potentially provide both mechanical support and biological stimulation [150,320]. These materials may be engineered to promote fibroblast activation, collagen deposition, hydration, and tissue repair while gradually degrading within the dermal environment [321,322]. Future formulations may also combine structural support with the controlled release of regenerative molecules [323].

Artificial intelligence-assisted skin assessment may contribute to more precise treatment planning and outcome evaluation [324]. Imaging-based systems can quantify wrinkles, pigmentation, pores, texture, erythema, laxity, and other visible skin characteristics more consistently than visual inspection alone [325]. These technologies may assist clinicians in product selection, protocol customization, longitudinal monitoring, and comparison of treatment outcomes [326]. Integration with three-dimensional imaging, ultrasound, and biomechanical measurements may further improve the precision of aesthetic assessment [327,328,329].

These developments face practical barriers beyond biological plausibility. Personalized selection algorithms require validation against clinically meaningful outcomes, and AI-assisted assessments require external validation across skin types and imaging conditions. Advanced delivery systems must demonstrate reproducible dosing, tissue exposure, stability, and biocompatibility. Combination protocols require matched procedural comparators to distinguish the incremental effects of the preparation and procedure. Across these approaches, transparent reporting, manufacturing reproducibility, regulatory evaluation, and extended safety follow-up are essential for clinical translation.

## 12. Conclusions

Biomodulators and skin boosters should not be treated as interchangeable intervention classes. Their composition, tissue exposure, therapeutic objectives, and clinical evidence differ. Mechanistic and preclinical findings support biological plausibility, but hydration changes, histological responses, clinical appearance, and patient-reported benefit are distinct outcomes and cannot substitute for one another. The updated search identified 159 comparative reports, but this report count is neither an independent-trial total nor a certainty grade. Risk-of-bias concerns, heterogeneous formulations and protocols, retrospective selection of one result per report, and unresolved access or eligibility constrain confidence. Results were not uniformly positive, and active-protocol comparisons do not establish category-wide efficacy against no treatment. Procedure-assisted EV/secretome studies do not establish an isolated exosome effect or the efficacy and long-term safety of direct injectable exosome therapy. Clinical conclusions should remain tied to the product, formulation, route, indication, comparator, follow-up, and outcome investigated. Future progress requires appropriately controlled trials, standardized and clinically meaningful outcomes, reproducible manufacturing, transparent regulatory assessment, and extended product-specific safety surveillance. Interpretation is further constrained by the PubMed/MEDLINE-only systematic update, the absence of de novo reassessment for every historical record, and the predominance of High overall risk-of-bias judgments (123/159). Accordingly, apparent clinical benefits should be considered provisional rather than confirmatory until supported by prospectively specified outcomes and rigorous, independently replicated trials.

## Figures and Tables

**Table 1:** Pragmatic Functional Framework for Biomodulators, Skin Boosters, and Related Biologic Preparations.

Category	Examples	Predominant Function	Key Qualification
HA-based skin boosters	Non-crosslinked or lightly crosslinked HA	Hydration and viscoelastic improvement	Effects vary according to formulation; some products may also provide tissue-support effects
Polynucleotide preparations	PN	Biostimulation/tissue repair	Evidence interpreted according to the PN formulation investigated
Polydeoxyribonucleotide preparations	PDRN	Repair-associated and anti-inflammatory effects	Not considered interchangeable with PN
Particulate collagen biostimulators	PLLA, CaHA, PCL	Neocollagenesis and ECM remodeling	Structural/volumetric and inflammatory effects vary by material and formulation
Multicomponent skin boosters	Amino acids, peptides, vitamins, antioxidants ± HA	Hydration and dermal-matrix support	Effects of individual components are often difficult to isolate
Autologous platelet-derived preparations	PRP, PRF	Growth-factor/cytokine delivery and tissue repair	Protocol-dependent autologous preparations
EV/exosome-containing preparations	Source-specific EV/exosome preparations	Emerging intercellular signaling	Manufacturing, characterization, route, and regulatory status vary
Secretome-derived preparations	Conditioned media/secretome preparations	Emerging paracrine signaling	Biologically distinct from isolated EV/exosome preparations

**Table 2:** Selected Comparative Human Clinical Evidence Discussed in the Main Text.

Intervention Category	Reports Discussed in the Main Text	No. Summarized Here	Main Outcome Domains	Key Interpretive Limitations
HA-based/multicomponent HA	Yang et al., 2026 [162]; Kerscher et al., 2021 [163]; Jiang et al., 2026 [164]; Fanian et al., 2023 [69]; Duteil et al., 2023 [72]; Chiu et al., 2023 [165]; Benzaquen et al., 2021 [166]	7	Hydration, wrinkles, elasticity, skin quality	Formulation/protocol heterogeneity
PLLA	Wu et al., 2024 [167]; Ponzo et al., 2026 [168]; Munoz-Lora et al., 2026 [77]; Joseph et al., 2026 [169]; Bravo et al., 2026 [170]; Arruda et al., 2024 [55]	6	Wrinkles, laxity, dermal remodeling, satisfaction	Different formulations/sites/protocols
CaHA/CaHA-HA hybrid	Pavicic et al., 2024 [66]; Faria et al., 2026 [171]; Faria et al., 2024 [172]; Fabi et al., 2026 [173]	4	Wrinkles, hydration, skin quality	Site- and dilution-specific
PCL	No PCL comparative report is summarized in this table	0	Not applicable	PCL findings are not generalized across formulations.
PRP-based	Tejero García et al., 2024 [174]; Pincelli et al., 2024 [62]; Li et al., 2025 [175]; Ismail et al., 2022 [176]; Abdalla et al., 2026 [177]	5	Rejuvenation, wrinkles, acne scars	Strong protocol/procedure heterogeneity
PN preparations	Araco and Araco, 2021 [178]	1	Atrophic acne scars	Single small randomized study
EV/secretome-based	Yusharyahya et al., 2023 [118]; Park et al., 2023 [99]; Huang et al., 2026 [179]	3	Wrinkles, texture, skin quality	Procedure-assisted protocols; heterogeneous comparators; inability to isolate exosome-specific effects; limited generalizability across formulations and delivery routes

Note: Table 2 summarizes selected product-specific comparisons discussed in the main narrative and is not the denominator of the updated search. Intervention categories were assigned for organization only. Findings from combination treatments were interpreted according to the complete treatment protocol and the actual comparator. Category membership does not imply independent efficacy of each biological component or generalizability to all formulations within that category.

## Data Availability

The search records, selection decisions, risk-of-bias assessments, evidence table, bibliography, and exclusion records supporting this review are provided in the files accompanying the reviewer submission. Copyrighted article bodies are not redistributed; lawful source links and record identifiers are provided where available.

## Supplementary Materials

The following supporting information can be downloaded at: https://www.mdpi.com/article/10.3390/ijms27219647/s1.

## Abbreviations

The following abbreviations are used in this manuscript:

CaHA	Calcium hydroxylapatite
CMC	Carboxymethylcellulose
DNA	Deoxyribonucleic acid
ECM	Extracellular matrix
ERK	Extracellular signal-regulated kinase
EV	Extracellular vesicle
HA	Hyaluronic acid
PCL	Polycaprolactone
PDRN	Polydeoxyribonucleotide
PLLA	Poly-L-lactic acid
PN	Polynucleotide
PN-HPT	Polynucleotide High-Purification Technology
PRF	Platelet-rich fibrin
PRISMA	Preferred Reporting Items for Systematic Reviews and Meta-Analyses
PRP	Platelet-rich plasma
RF	Radiofrequency
RoB 2	Risk of Bias 2
