# Supplementary material for: Biomodulators and Skin Boosters in Skin Rejuvenation: Mechanisms, Applications, and Future Perspectives
Source: Int J Mol Sci. 2026 Oct 28;27(21):9647. doi: 10.3390/ijms27219647 (PMC13762510; doi:10.3390/ijms27219647)
Supplement: Supplementary file 1 [file ijms-27-09647-s001.zip › Supplementary_Table_S1_Final.pdf]

Supplementary Table S1 Search and selection methods

A reproducible PubMed/MEDLINE search was completed on 16 October 2026. Nine product-focused strategies were executed with a publication-date ceiling of 16 October 2026 and without language, human-only, or trial-design retrieval filters. Screening, source retrieval, and eligibility adjudication followed predefined revision-stage criteria. This work was conducted as a structured critical narrative review and was not prospectively registered as a systematic review.

Item	Recorded method or result
Search	PubMed via NCBI E-utilities; nine strategies, 16:58:10–16:58:21 KST. Publication dates 1800/01/01–2026/10/16; no language, human-only or trial-design retrieval filter.
Eligibility	Adults ≥18 with aging/photoaging, wrinkles, laxity, impaired skin quality or atrophic acne scars; covered preparations delivered by injection or a skin-penetrating procedure; defined randomized between-treatment outcome. Mixed-age cohorts require separable adult results.
Outside the comparative update	Nonhuman/secondary reports, nonrandomized assignment, ordinary topical/oral exposure, isolated volumization or deep-fold filling without a separate skin-quality outcome. Adjacent/extension reports are separately labelled.
Selection	Records were screened against predefined title and abstract criteria and, when potentially eligible, by complete-source or source-level adjudication. A first sensitivity audit examined 365 titles, including 238 records with abstract or methods information. An expanded audit examined 1,898 records, including 1,318 with available abstract or original-source evidence and 580 with title-only evidence. These sequential audits were consistency checks and were not independent duplicate screening. Thirty additional exclusions advanced to retrieval.
Source review	Complete sources were sought through public repositories, DOI and publisher pages, lawful local files, and Seoul National University Library-linked routes. The 195-record recheck yielded 94 eligible comparative reports, 93 source or record exclusions, four title or abstract exclusions, and four access-unresolved reports. Unresolved reporting was retained as no information or as an unresolved eligibility status, as appropriate.
Scope limits	The update was limited to one systematically searched database, used revision-stage eligibility criteria, and selected one result retrospectively per comparative report. Independent duplicate screening was not performed. Four reports remain access-unresolved, and eight retrieved reports retain design or adult-eligibility uncertainty.

Selection counts

The nine strategies returned 5,963 record occurrences. Exact PMID deduplication removed 1,015 repeat appearances and yielded 4,948 unique records. The final flow comprised 4,557 title and abstract exclusions and 391 full-text candidates. Of these candidates, 387 reports were assessed or source-adjudicated and four remained access-unresolved. Retrieval was attempted for 530 reports across all review passes; 139 of those reports were ultimately excluded at title and abstract level. The focused recheck of 195 previously unavailable candidates directly verified 145 complete texts, identified authoritative full-text links for 42, excluded four records on explicit title or abstract evidence, and left four access-unresolved.

The 387 assessed or adjudicated reports comprised 209 exclusions, 159 comparative reports, eight contextual reports, three noncomparative primary estimates, and eight retrieved reports with unresolved design or adult-eligibility questions. One comparison, outcome, and assessment time were specified retrospectively for each comparative report. Report counts are document-level counts and are not independent-trial totals or certainty grades. When source reporting did not resolve a methodological question, the uncertainty was retained explicitly rather than inferred.

Companion records provide the audit trail: S1B contains the exact searches; S1C contains all 4,948 record dispositions; S1D preserves the 530-report retrieval and eligibility history and the 195-record recheck; S2B and the signaling file support the 159 result-specific risk-of-bias assessments; S3B and S3C provide the comparative evidence table and bibliography; and S4B documents the 209 source or full-text exclusions. Access status, eligibility decision, evidence basis, and appraisal status are recorded separately.

Executable search components

Each original strategy is reproduced exactly as: (product block Pi) AND (common block C) AND (date block D). The outer parentheses are already included below. Copy the three components with the two AND operators; no retrieval filters were added.

Common block C

("Skin Aging"[Mesh] OR "Rejuvenation"[Mesh] OR "skin remodeling"[tiab] OR "skin remodelling"[tiab] OR "skin revital*"[tiab] OR "skin texture"[tiab] OR "skin smoothness"[tiab] OR "cutaneous hydration"[tiab] OR ("Cicatrix"[Mesh] AND "Acne Vulgaris"[Mesh]) OR rejuvenat*[tiab] OR photoag*[tiab] OR antiaging[tiab] OR "anti-aging"[tiab] OR wrinkle*[tiab] OR rhytid*[tiab] OR "skin quality"[tiab] OR "skin aging"[tiab] OR "skin ageing"[tiab] OR "skin laxity"[tiab] OR "skin elasticity"[tiab] OR "skin hydration"[tiab] OR "skin roughness"[tiab] OR "dermal remodeling"[tiab] OR "dermal remodelling"[tiab] OR "acne scar*"[tiab] OR "atrophic scar*"[tiab] OR "neck line*"[tiab] OR "neck wrinkle*"[tiab] OR "periorbital hollow*"[tiab])

Date block D: ("1800/01/01"[Date - Publication] : "2026/10/16"[Date - Publication])

Product block	Exact product terms	KST time	n
P1 HA	("Hyaluronic Acid"[Mesh] OR "hyaluronic acid"[tiab] OR hyaluronan[tiab] OR hyaluronate[tiab] OR "VYC-12*"[tiab] OR Profhilo[tiab] OR Restylane[tiab] OR "polydensified matrix"[tiab] OR "NCTF 135"[tiab] OR Redensity[tiab])	16:58:10	2785
P2 PLA	("Polylactic Acid-Polyglycolic Acid Copolymer"[Mesh] OR "polylactic acid"[tiab] OR "poly-l-lactic acid"[tiab] OR "poly-d,l-lactic acid"[tiab] OR "poly-D-L-lactic acid"[tiab] OR PLLA[tiab] OR PDLLA[tiab] OR Sculptra[tiab] OR Aesthefill[tiab] OR Juvelook[tiab] OR Lanluma[tiab] OR Elleva[tiab])	16:58:12	491
P3 CaHA	("calcium hydroxylapatite"[tiab] OR "calcium hydroxyapatite"[tiab] OR CaHA[tiab] OR Radiesse[tiab] OR HArmonyCa[tiab])	16:58:15	389
P4 PCL	(polycaprolactone[tiab] OR "poly-caprolactone"[tiab] OR "poly-epsilon-caprolactone"[tiab] OR Ellanse[tiab] OR Ellansé[tiab] OR (PCL[tiab] AND (filler*[tiab] OR skin[tiab] OR dermal[tiab])))	16:58:16	113
P5 PN/PDRN	(polynucleotide*[tiab] OR polydeoxyribonucleotide*[tiab] OR PDRN[tiab] OR "PN-HPT"[tiab] OR Rejuran[tiab] OR Plinest[tiab] OR Nucleofill[tiab] OR PhilArt[tiab])	16:58:17	89
P6 PRP/PRF	("Platelet-Rich Plasma"[Mesh] OR "Platelet-Rich Fibrin"[Mesh] OR "platelet rich plasma"[tiab] OR "platelet-rich plasma"[tiab] OR "platelet rich fibrin"[tiab] OR "platelet-rich fibrin"[tiab] OR PRP[tiab] OR "i-PRF"[tiab] OR "injectable PRF"[tiab])	16:58:18	627
P7 EV/exosomes	("Exosomes"[Mesh] OR "Extracellular Vesicles"[Mesh] OR exosom*[tiab] OR "extracellular vesicle*"[tiab] OR "nanovesicle*"[tiab])	16:58:19	711
P8 Secretome	("Secretome"[Mesh] OR secretom*[tiab] OR "conditioned medium"[tiab] OR "conditioned media"[tiab] OR "stem cell conditioned"[tiab])	16:58:20	316
P9 Boosters	("skin booster*"[tiab] OR skinbooster*[tiab] OR "Mesotherapy"[Mesh] OR mesotherap*[tiab] OR biorevitali*[tiab] OR bioremodelling[tiab] OR bioremodeling[tiab] OR biostimulat*[tiab] OR biomodulat*[tiab])	16:58:21	442

All nine component-based reconstructions were checked against the archived submitted strings. Full timestamps, PubMed translations and raw-search provenance remain in the companion records. Later indexing changes can alter rerun counts.
