# Supplementary material for: Biomodulators and Skin Boosters in Skin Rejuvenation: Mechanisms, Applications, and Future Perspectives
Source: Int J Mol Sci. 2026 Oct 28;27(21):9647. doi: 10.3390/ijms27219647 (PMC13762510; doi:10.3390/ijms27219647)
Supplement: Supplementary file 1 [file ijms-27-09647-s001.zip › Supplementary_Table_S2_Final.pdf]

Supplementary Table S2 Risk of bias summary for 159 specified results

Risk of bias was assessed using the Cochrane RoB 2 tool dated 22 August 2019 for the effect of assignment to intervention. The table contains 159 specified results, 795 domain judgments, and 3,498 signaling-response slots, including conditional not-applicable responses. Overall, 123 results were judged High and 36 raised Some concerns; none was Low across all five domains. The same signaling pathways were applied throughout. When the available report, protocol, registration, or analysis-plan information did not resolve a signaling question, the response was recorded as No information and mapped through the published decision pathway without override.

L = Low; S = Some concerns; H = High. D1, randomization process; D2, deviations from intended interventions; D3, missing outcome data; D4, measurement of the outcome; D5, selection of the reported result. Each judgment applies to the comparison, outcome, and assessment time specified in Supplementary Table S3. The final column summarizes the principal source-specific issues. S2B provides the five domain rationales and source locators for every result, and the signaling file preserves all signaling responses.

Result / PMID	D1	D2	D3	D4	D5	Overall	Principal source-specific issues
R01 23715889	S	S	H	L	S	H	D2: one allocation inversion; randomized status of untreated enrollees unclear. D3: selected radiance coverage unresolved. D4: 16 masked experts. D5: secondary-result plan unverified.
R02 25324650	L	L	L	L	S	S	Independent sequence custody and sequential envelopes support concealment; all 25 completed, with masked photographic grading. D5: a timely plan for the selected result was not verified.
R03 26924405	S	S	L	L	S	S	D1: allocation details absent. D2: awareness-related care unresolved. All 16 completed; two masked graders. D5: pre-unblinding analysis plan unverified.
R04 27222038	S	S	L	S	S	S	Excel sequence; concealment and awareness-related care unclear. All 13 completed. D4: VISIA acquisition/region masking unresolved. D5: endpoint/analysis plan unverified.
R05 30210162	S	S	H	H	S	H	D3: 52/55 completed; reasons and missingness robustness unresolved. D4: clinical assessor masking unreported. D1/D2/D5: allocation, nonprotocol care and analysis specification uncertain.
R06 30419125	L	L	H	L	S	H	Central sequence custody and masked grading; 27 enrolled but only 19 complete cases. D3: robustness unestablished. D5: selection among fine-line subscores/visits not prospectively verified.
R07 31420849	S	S	L	L	S	S	Random-number cards used; concealment unclear. Relevant groups retain 25 each and masked graders. D2: awareness-related care unresolved. D5: final timing and analysis plan uncertain.
R08 33911590	S	L	H	L	S	H	Identical patches mask treatment and Antera assessment. D3: two withdrawals among 52; allocation-specific final denominators unclear. D1/D5: concealment and Day 29 selection plan unverified.
R09 31769596	L	S	H	S	S	H	eCRF assignment supports concealment. D3: seven withdrawals among 100; Month 3 observed hydration counts unclear. D4: acquisition masking uncertain. D2/D5: care/analysis specification unresolved.
R10 33073298	L	L	L	L	S	S	Independent sequence and sequential sealed envelopes; matched protocols; all 25 completed with masked ECCA grading. D5: dated prospective analysis plan not verified.
R11 33755110	L	L	L	S	S	S	Protected electronic allocation and all 20 complete. D4: instrumental acquisition masking unclear. D5: unverified plan and internally inconsistent scales/captions require caution.
R12 34758056	S	S	L	L	S	S	D1/D2: sequence, concealment and awareness-related care unclear. Forty-eight of 49 completed; clinical raters masked. D5: selected-result analysis plan unverified.
R13 35501660	S	L	L	S	S	S	D1: randomization method/concealment unclear. Twenty-nine of 30 completed. D4: Antera operator masking unresolved. D5: several measures/visits without a verified selection plan.

Result / PMID	D1	D2	D3	D4	D5	Overall	Principal source-specific issues
R14 35685406	S	L	L	L	S	S	D1: computer-generated random numbers selected sides by parity; concealment unresolved. Two withdrawals had job-related reasons; 28 completed. Masked grading and matched care support other domains; prospective plan unverified.
R15 35689521	S	L	H	L	S	H	Computer sequence, concealment unclear. D3: 14/15 observed; missingness robustness unverified. Masked evaluator. D5: dated plan unverified; reported degrees of freedom conflict with pairing.
R16 36129233	S	L	L	L	S	S	D1: sequence/concealment incomplete. Participants/investigators masked to anesthetic; selected Day 30 outcomes reported for 30. D5: prospective plan for secondary aesthetics unverified.
R17 36564573	L	S	H	L	S	H	Opaque treatment-card envelope supports concealment. D3: 40 patients versus three 20-observation cells; endpoint linkage unclear. Masked grading. D2/D5: nonprotocol care and analysis specification uncertain.
R18 36520210	S	S	L	H	S	H	Computerized product assignment; concealment unclear. D3: 15/product group accounted for. D4: scar-assessor masking unestablished. Fixed-side needling is not randomized; prospective plan unverified.
R19 36798539	S	L	L	L	S	S	D1: sequence custody/concealment unclear. All 30 completed; DPAS assessors masked and care matched. D5: multiple registered outcomes without a verified dated selected-analysis plan.
R20 36909864	S	L	H	L	S	H	Blocked sequence, concealment unreported. D3: 30 randomized, 28 per protocol; selected-outcome robustness unresolved. Masked replica analysis. D5: wrinkle-depth-class selection plan unverified.
R21 37074002	L	L	H	H	S	H	Central IWRS allocation. D3: observed FACE-Q counts 122/131 versus 47/71; response-related loss probable. D4: unblinded satisfaction. D5: retrieved SAP postdates completion; predecessor unavailable.
R22 37163665	L	L	H	L	S	H	Central IWRS; masked ACSS evaluator. D3: imputed 131/71 totals do not resolve observed denominators. D5: September 2020 SAP postdates July completion; earlier SAP unavailable.
R23 37377400	S	S	L	H	S	H	Block allocation; concealment and awareness-related care unclear. All 28 completed. D4: GAIS assessor masking unestablished. D5: several visits/endpoints without a verified hierarchy.
R24 37577796	S	L	H	S	S	H	D3: four treated withdrawals; Day 75 observed denominator unclear. D4: LifeViz acquisition masking unestablished. D5: registration in 2022 followed 2019 completion; multiple one-sided tests.
R25 37631060	S	S	L	S	S	S	All 20 completed. D1/D2: sequence, concealment and awareness-related care unclear. D4: TEWL operator masking uncertain. D5: selected-analysis plan unverified.
R26 37823019	S	S	L	H	S	H	Fifteen Month 3 grades accounted for. D4: patient versus researcher GAIS attribution/masking unclear. D1/D2/D5: allocation, nonprotocol care and selected-analysis plan unresolved.
R27 38314374	S	S	L	H	S	H	All 60 final clinical distributions accounted for. D4: explicitly unblinded scar grading. D1/D2/D5: concealment, trial-related care and analysis plan insufficiently documented.
R28 38174839	L	L	L	L	S	S	Coded sealed envelopes; participant/image-evaluator masking; eight/group outcomes accounted for. Small-group baseline imbalance does not itself prove allocation failure. D5: prospective selected-analysis plan unverified.
R29 38798929	S	L	H	H	S	H	D3: observed Week 24 coverage and LOCF robustness unresolved. D4: roughness-examiner masking unclear. Shared MN/skincare standardized. D1/D5: allocation and prospective plan incomplete.
R30 38916005	L	S	H	H	S	H	Opaque envelopes; sequence custody limited. D3: final score denominators unclear. D4: assessor masking unestablished despite generic blinding claims. D2/D5: awareness-related care and plan uncertain.
R31 39122500	S	S	L	H	S	H	Sealed envelopes; opacity/custody unclear. Final paired counts support completeness. D4: clinical assessor masking unreported. D2/D5: awareness-related care and selected-analysis plan unresolved.

Result / PMID	D1	D2	D3	D4	D5	Overall	Principal source-specific issues
R32 39231078	S	L	L	L	S	S	All ten completed with blinded clinical ratings. D1: sequence/concealment unclear; unequal 7/3 groups alone do not prove failure. D5: customized measures and unverified prospective plan.
R33 39356623	S	S	H	H	S	H	D3: 40/41 per protocol; dropout relationship unresolved. D4: radiance-assessor masking unreported. D1/D2/D5: allocation, awareness-related care and endpoint hierarchy unclear.
R34 39370844	S	S	L	L	S	S	All ten paired final GAIS ratings accounted for; investigator masking probable. D1/D2/D5: allocation details, awareness-related additional care and analysis hierarchy unresolved.
R35 39503284	S	L	L	L	S	S	All 15 completed; masked Lemperle graders and planned procedures. D1: sequence/concealment unspecified. D5: multiple visits/measures without a verified prospective analysis hierarchy.
R36 39852991	S	S	H	S	S	H	D3: Month 1 roughness denominators unclear. D4: Antera region/acquisition masking uncertain. D1/D2/D5: concealment, nonprotocol care and early-endpoint selection plan unverified.
R37 40296541	S	S	L	H	S	H	All 30 completed. D4: clinical neck-grade masking unreported. D1/D2/D5: randomization method, awareness-related additional care and six-month analysis plan unclear.
R38 40304039	S	S	L	L	S	S	Twenty-nine of 30 completed; one inconvenient follow-up withdrawal unlikely materially to affect this paired endpoint. Masked pore grader. D1/D2/D5: allocation, masking roles and analysis selection uncertain.
R39 40476635	S	S	H	H	S	H	D3: complete-record eligibility obscures randomized versus observed GSS denominators. D4: independent physicians not explicitly masked. D1/D2/D5: allocation, nonprotocol care and plan unclear.
R40 40509910	S	S	H	L	S	H	D1: conflicting randomization ratios. D3: small subgroup denominators/loss unclear. Masked GAIS evaluator. D2/D5: additional-treatment care and exploratory analysis specification unresolved.
R41 40599706	L	S	L	L	S	S	Computer sequence and opaque envelopes; all 30 completed with masked photoaging grading. D2: injector awareness-related care unresolved. D5: prospective endpoint/analysis plan unverified.
R42 40665746	S	S	L	H	S	H	All ten paired outcomes accounted for. D4: scar-grader masking unreported. D1/D2/D5: sequence/concealment, awareness-related care and selected paired-analysis plan unclear.
R43 40814769	L	L	L	L	S	S	Independent allocation, opaque envelopes, simulated cannula control; all 25 completed with masked EMG acquisition. D5: prospective selected-analysis plan unverified; secondary paired-analysis concerns remain separate.
R44 40853048	S	S	H	S	S	H	D3: final denominators among eight participants insufficiently explicit. D4: VisioFace acquisition/region masking uncertain. D1/D2/D5: concealment, staff awareness-related care and endpoint hierarchy unclear.
R45 41585978	L	S	L	L	S	S	SNOSE concealment; all 15 pairs assessed by a blinded observer. D2: participant/operator awareness-related care unresolved. D5: dated prospective plan unverified; between-side testing ignores pairing.
R46 41442012	S	S	L	S	S	S	All 24 completed. D1/D2: concealment and preparing-team awareness-related deviations unclear. D4: Antera acquisition masking unresolved despite masked photographic reviewers. D5: analysis plan unverified.
R47 41669823	L	L	H	L	S	H	Opaque envelopes and coded preparation; masked HGS graders. D3: Day 90 observed denominators/robustness unresolved. D5: dated prospective plan unverified; in vitro work assessed separately.
R48 41828422	S	L	L	L	S	S	Independent SAS sequence; concealment mechanism unspecified. Masked treatment/assessment; Figure 1 confirms all 20 pairs analyzed. D5: September 2025 registration follows May completion.
R49 41873431	L	L	H	L	S	H	IVRS allocation and masked evaluators. D3: 180 analyzed versus randomized-denominator rates/BOCF; observed coverage unresolved. D5: prospective noninferiority plan not independently verified.

Result / PMID	D1	D2	D3	D4	D5	Overall	Principal source-specific issues
R50 41971651	S	S	H	L	S	H	D3: 212/224 versus 209/224 completed; roughness-outcome robustness unestablished. Masked photographic assessors. D1/D2/D5: allocation, awareness-related care and plan unclear.
R51 42063688	S	S	H	L	S	H	D3: final photographic-score denominators unclear. Masked dermatologists. D1/D2: concealment and additional-care details incomplete. D5: registration follows stated study completion.
R52 42087429	L	L	L	L	S	S	Concealed software allocation, blinded ultrasound operator and all 20 Day 120 outcomes. D5: prospective selected-analysis plan unverified. Fixed injection planes are outside randomized product assignment.
R53 42181534	S	L	H	L	S	H	D3: outcome-specific follow-up among 20 participants insufficiently explicit. Physician GAIS graders masked. D1/D5: allocation details and prospective analysis plan unverified; paired inference needs checking.
R54 42225690	L	L	L	L	S	S	Independent sequence/opaque envelopes; injector, participants and assessors masked. Twenty-seven of 28 attended six months; small paired loss judged unlikely material. D5: retrospective registration.
R55 42235050	S	L	L	H	L	H	D4: open-label treating-investigator GDS. Concealment unestablished; ITT missing-data sensitivity analyses support robustness. D5: protocol dated May 2022 prespecifies Month 9 endpoint and analysis.
R56 42367716	S	L	H	L	S	H	D3: 439/456 completed; selected response denominator/robustness unresolved. Masked photographic panel. D1/D5: allocation procedure and timely analysis plan unverified; skincare standardized.
R57 42410192	S	S	L	L	S	S	D1/D2: concealment and awareness-related care unclear. One control relocated (80/79 analyzed), unlikely materially to affect this continuous outcome. Masked scar graders; prospective plan unverified.
R58 42420660	L	S	L	L	S	S	Independent sequence/opaque envelopes and masked outcome investigators. All 24 completed; pre-intervention death precedes side randomization. D2/D5: injector-related deviations and secondary-analysis specification unresolved.
R59 42621380	L	S	L	H	S	H	Independent computer allocation and sealed envelopes; 50/group outcomes accounted for. D4: ECCA-assessor masking unreported. D2/D5: awareness-related care and prospective analysis specification unresolved.
R60 42847981	S	S	L	L	S	S	Masked live GDS evaluator; ITT/observed-case/worst-/best-case analyses support missingness robustness. D1/D2/D5: allocation procedure, awareness-related care and timely SAP unresolved; 19 safety-only participants excluded.
R61 34608092	L	S	L	L	S	S	eCRF allocation; one wrongly treated control excluded. Registry confirms LOCF; extreme-case check supports missing-data robustness. Pre-unblinding plan timing remains unverified.
R62 36892373	S	S	L	H	S	H	Concealment/blinding roles unspecified. All 34 completed. Physician masking unresolved for a subjective grade. Multiple outcomes/time points without a verified plan.
R63 37594172	S	L	H	H	S	H	Random-side method and concealment unspecified. Paired testing. Crow's-feet observed denominators and observer masking unresolved; no verified prospective hierarchy.
R64 37833618	S	S	L	L	S	S	Sequence/concealment unspecified. One implementation-error exclusion from 33; 32 per-protocol. Blinded grader. Several outcomes/time points without a verified hierarchy.
R65 42339013	L	L	L	H	S	H	Computer sequence and central allocation; all 40 assessed at 12 months. Open-label physician WSRS with probable influence on scoring. No verified dated pre-unblinding analysis plan.
R66 | 12859378	S	S	H	L	S	H	D1: sequence/concealment incompletely verified. D2/D4 reflect reported masking or protocol safeguards where available. D3 is High unless selected-outcome completion was explicit. D5: prespecified result/time plan unverified.
R67 | 18547178	S	S	H	L	S	H	D1: sequence/concealment incompletely verified. D2/D4 reflect reported masking or protocol safeguards where available. D3 is High unless selected-outcome completion was explicit. D5: prespecified result/time plan unverified.

Result / PMID	D1	D2	D3	D4	D5	Overall	Principal source-specific issues
R68 | 19735521	S	S	H	L	S	H	D1: sequence/concealment incompletely verified. D2/D4 reflect reported masking or protocol safeguards where available. D3 is High unless selected-outcome completion was explicit. D5: prespecified result/time plan unverified.
R69 | 21070457	S	S	H	S	S	H	D1: sequence/concealment incompletely verified. D2/D4 reflect reported masking or protocol safeguards where available. D3 is High unless selected-outcome completion was explicit. D5: prespecified result/time plan unverified.
R70 | 21134044	S	S	H	S	S	H	D1: sequence/concealment incompletely verified. D2/D4 reflect reported masking or protocol safeguards where available. D3 is High unless selected-outcome completion was explicit. D5: prespecified result/time plan unverified.
R71 | 21635618	S	L	L	S	S	S	D1: sequence/concealment incompletely verified. D2/D4 reflect reported masking or protocol safeguards where available. D3 is High unless selected-outcome completion was explicit. D5: prespecified result/time plan unverified.
R72 | 22288389	S	S	H	S	S	H	D1: sequence/concealment incompletely verified. D2/D4 reflect reported masking or protocol safeguards where available. D3 is High unless selected-outcome completion was explicit. D5: prespecified result/time plan unverified.
R73 | 23545926	S	L	H	L	S	H	D1: sequence/concealment incompletely verified. D2/D4 reflect reported masking or protocol safeguards where available. D3 is High unless selected-outcome completion was explicit. D5: prespecified result/time plan unverified.
R74 | 24245880	S	S	H	L	S	H	D1: sequence/concealment incompletely verified. D2/D4 reflect reported masking or protocol safeguards where available. D3 is High unless selected-outcome completion was explicit. D5: prespecified result/time plan unverified.
R75 | 24305424	S	L	H	S	S	H	D1: sequence/concealment incompletely verified. D2/D4 reflect reported masking or protocol safeguards where available. D3 is High unless selected-outcome completion was explicit. D5: prespecified result/time plan unverified.
R76 | 24354616	S	L	H	S	S	H	D1: sequence/concealment incompletely verified. D2/D4 reflect reported masking or protocol safeguards where available. D3 is High unless selected-outcome completion was explicit. D5: prespecified result/time plan unverified.
R77 | 24566300	S	S	H	S	S	H	D1: sequence/concealment incompletely verified. D2/D4 reflect reported masking or protocol safeguards where available. D3 is High unless selected-outcome completion was explicit. D5: prespecified result/time plan unverified.
R78 | 25006854	S	S	L	S	S	S	D1: sequence/concealment incompletely verified. D2/D4 reflect reported masking or protocol safeguards where available. D3 is High unless selected-outcome completion was explicit. D5: prespecified result/time plan unverified.
R79 | 25539986	S	L	L	L	S	S	D1: sequence/concealment incompletely verified. D2/D4 reflect reported masking or protocol safeguards where available. D3 is High unless selected-outcome completion was explicit. D5: prespecified result/time plan unverified.
R80 | 25828037	S	S	H	L	S	H	D1: sequence/concealment incompletely verified. D2/D4 reflect reported masking or protocol safeguards where available. D3 is High unless selected-outcome completion was explicit. D5: prespecified result/time plan unverified.
R81 | 26606435	S	S	H	S	S	H	D1: sequence/concealment incompletely verified. D2/D4 reflect reported masking or protocol safeguards where available. D3 is High unless selected-outcome completion was explicit. D5: prespecified result/time plan unverified.
R82 | 26618456	S	S	H	S	S	H	D1: sequence/concealment incompletely verified. D2/D4 reflect reported masking or protocol safeguards where available. D3 is High unless selected-outcome completion was explicit. D5: prespecified result/time plan unverified.
R83 | 26735291	S	S	H	L	S	H	D1: sequence/concealment incompletely verified. D2/D4 reflect reported masking or protocol safeguards where available. D3 is High unless selected-outcome completion was explicit. D5: prespecified result/time plan unverified.
R84 | 26910661	S	L	H	S	S	H	D1: sequence/concealment incompletely verified. D2/D4 reflect reported masking or protocol safeguards where available. D3 is High unless selected-outcome completion was explicit. D5: prespecified result/time plan unverified.
R85 | 27503619	S	S	H	S	S	H	D1: sequence/concealment incompletely verified. D2/D4 reflect reported masking or protocol safeguards where available. D3 is High unless selected-outcome completion was explicit. D5: prespecified result/time plan unverified.
R86 | 28157728	S	S	H	S	S	H	D1: sequence/concealment incompletely verified. D2/D4 reflect reported masking or protocol safeguards where available. D3 is High unless selected-outcome completion was explicit. D5: prespecified result/time plan unverified.
R87 | 28382785	S	L	H	L	S	H	D1: sequence/concealment incompletely verified. D2/D4 reflect reported masking or protocol safeguards where available. D3 is High unless selected-outcome completion was explicit. D5: prespecified result/time plan unverified.
R88 | 28504480	S	S	H	S	S	H	D1: sequence/concealment incompletely verified. D2/D4 reflect reported masking or protocol safeguards where available. D3 is High unless selected-outcome completion was explicit. D5: prespecified result/time plan unverified.

Result / PMID	D1	D2	D3	D4	D5	Overall	Principal source-specific issues
R89 | 28595250	S	L	H	L	S	H	D1: sequence/concealment incompletely verified. D2/D4 reflect reported masking or protocol safeguards where available. D3 is High unless selected-outcome completion was explicit. D5: prespecified result/time plan unverified.
R90 | 28892233	S	S	H	L	S	H	D1: sequence/concealment incompletely verified. D2/D4 reflect reported masking or protocol safeguards where available. D3 is High unless selected-outcome completion was explicit. D5: prespecified result/time plan unverified.
R91 | 28987023	S	S	H	S	S	H	D1: sequence/concealment incompletely verified. D2/D4 reflect reported masking or protocol safeguards where available. D3 is High unless selected-outcome completion was explicit. D5: prespecified result/time plan unverified.
R92 | 29019860	S	L	H	L	S	H	D1: sequence/concealment incompletely verified. D2/D4 reflect reported masking or protocol safeguards where available. D3 is High unless selected-outcome completion was explicit. D5: prespecified result/time plan unverified.
R93 | 29226630	S	S	H	L	S	H	D1: sequence/concealment incompletely verified. D2/D4 reflect reported masking or protocol safeguards where available. D3 is High unless selected-outcome completion was explicit. D5: prespecified result/time plan unverified.
R94 | 29266290	S	S	H	L	S	H	D1: sequence/concealment incompletely verified. D2/D4 reflect reported masking or protocol safeguards where available. D3 is High unless selected-outcome completion was explicit. D5: prespecified result/time plan unverified.
R95 | 29381544	S	L	L	S	S	S	D1: sequence/concealment incompletely verified. D2/D4 reflect reported masking or protocol safeguards where available. D3 is High unless selected-outcome completion was explicit. D5: prespecified result/time plan unverified.
R96 | 29762642	S	S	H	S	S	H	D1: sequence/concealment incompletely verified. D2/D4 reflect reported masking or protocol safeguards where available. D3 is High unless selected-outcome completion was explicit. D5: prespecified result/time plan unverified.
R97 | 30076447	S	L	H	L	S	H	D1: sequence/concealment incompletely verified. D2/D4 reflect reported masking or protocol safeguards where available. D3 is High unless selected-outcome completion was explicit. D5: prespecified result/time plan unverified.
R98 | 30105816	S	S	H	L	S	H	D1: sequence/concealment incompletely verified. D2/D4 reflect reported masking or protocol safeguards where available. D3 is High unless selected-outcome completion was explicit. D5: prespecified result/time plan unverified.
R99 | 30265171	S	L	H	S	S	H	D1: sequence/concealment incompletely verified. D2/D4 reflect reported masking or protocol safeguards where available. D3 is High unless selected-outcome completion was explicit. D5: prespecified result/time plan unverified.
R100 | 30375189	S	L	H	L	S	H	D1: sequence/concealment incompletely verified. D2/D4 reflect reported masking or protocol safeguards where available. D3 is High unless selected-outcome completion was explicit. D5: prespecified result/time plan unverified.
R101 | 30534955	S	L	H	L	S	H	D1: sequence/concealment incompletely verified. D2/D4 reflect reported masking or protocol safeguards where available. D3 is High unless selected-outcome completion was explicit. D5: prespecified result/time plan unverified.
R102 | 30741790	S	L	H	L	S	H	D1: sequence/concealment incompletely verified. D2/D4 reflect reported masking or protocol safeguards where available. D3 is High unless selected-outcome completion was explicit. D5: prespecified result/time plan unverified.
R103 | 30924301	S	S	H	S	S	H	D1: sequence/concealment incompletely verified. D2/D4 reflect reported masking or protocol safeguards where available. D3 is High unless selected-outcome completion was explicit. D5: prespecified result/time plan unverified.
R104 | 30964227	S	S	H	L	S	H	D1: sequence/concealment incompletely verified. D2/D4 reflect reported masking or protocol safeguards where available. D3 is High unless selected-outcome completion was explicit. D5: prespecified result/time plan unverified.
R105 | 31172821	S	L	H	L	S	H	D1: sequence/concealment incompletely verified. D2/D4 reflect reported masking or protocol safeguards where available. D3 is High unless selected-outcome completion was explicit. D5: prespecified result/time plan unverified.
R106 | 31180258	S	L	H	L	S	H	D1: sequence/concealment incompletely verified. D2/D4 reflect reported masking or protocol safeguards where available. D3 is High unless selected-outcome completion was explicit. D5: prespecified result/time plan unverified.
R107 | 31241854	S	S	H	L	S	H	D1: sequence/concealment incompletely verified. D2/D4 reflect reported masking or protocol safeguards where available. D3 is High unless selected-outcome completion was explicit. D5: prespecified result/time plan unverified.
R108 | 31328871	S	L	H	L	S	H	D1: sequence/concealment incompletely verified. D2/D4 reflect reported masking or protocol safeguards where available. D3 is High unless selected-outcome completion was explicit. D5: prespecified result/time plan unverified.
R109 | 31334927	S	S	H	S	S	H	D1: sequence/concealment incompletely verified. D2/D4 reflect reported masking or protocol safeguards where available. D3 is High unless selected-outcome completion was explicit. D5: prespecified result/time plan unverified.

Result / PMID	D1	D2	D3	D4	D5	Overall	Principal source-specific issues
R110 | 31680395	S	L	H	L	S	H	D1: sequence/concealment incompletely verified. D2/D4 reflect reported masking or protocol safeguards where available. D3 is High unless selected-outcome completion was explicit. D5: prespecified result/time plan unverified.
R111 | 32108322	S	S	H	S	S	H	D1: sequence/concealment incompletely verified. D2/D4 reflect reported masking or protocol safeguards where available. D3 is High unless selected-outcome completion was explicit. D5: prespecified result/time plan unverified.
R112 | 32248707	S	S	H	S	S	H	D1: sequence/concealment incompletely verified. D2/D4 reflect reported masking or protocol safeguards where available. D3 is High unless selected-outcome completion was explicit. D5: prespecified result/time plan unverified.
R113 | 32333512	S	S	H	S	S	H	D1: sequence/concealment incompletely verified. D2/D4 reflect reported masking or protocol safeguards where available. D3 is High unless selected-outcome completion was explicit. D5: prespecified result/time plan unverified.
R114 | 32385943	S	L	L	L	S	S	D1: sequence/concealment incompletely verified. D2/D4 reflect reported masking or protocol safeguards where available. D3 is High unless selected-outcome completion was explicit. D5: prespecified result/time plan unverified.
R115 | 32421218	S	S	H	L	S	H	D1: sequence/concealment incompletely verified. D2/D4 reflect reported masking or protocol safeguards where available. D3 is High unless selected-outcome completion was explicit. D5: prespecified result/time plan unverified.
R116 | 32485024	S	S	H	L	S	H	D1: sequence/concealment incompletely verified. D2/D4 reflect reported masking or protocol safeguards where available. D3 is High unless selected-outcome completion was explicit. D5: prespecified result/time plan unverified.
R117 | 32506124	S	L	H	L	S	H	D1: sequence/concealment incompletely verified. D2/D4 reflect reported masking or protocol safeguards where available. D3 is High unless selected-outcome completion was explicit. D5: prespecified result/time plan unverified.
R118 | 32621657	S	L	H	L	S	H	D1: sequence/concealment incompletely verified. D2/D4 reflect reported masking or protocol safeguards where available. D3 is High unless selected-outcome completion was explicit. D5: prespecified result/time plan unverified.
R119 | 32770685	S	S	H	S	S	H	D1: sequence/concealment incompletely verified. D2/D4 reflect reported masking or protocol safeguards where available. D3 is High unless selected-outcome completion was explicit. D5: prespecified result/time plan unverified.
R120 | 32816021	S	L	H	L	S	H	D1: sequence/concealment incompletely verified. D2/D4 reflect reported masking or protocol safeguards where available. D3 is High unless selected-outcome completion was explicit. D5: prespecified result/time plan unverified.
R121 | 32852426	S	L	H	L	S	H	D1: sequence/concealment incompletely verified. D2/D4 reflect reported masking or protocol safeguards where available. D3 is High unless selected-outcome completion was explicit. D5: prespecified result/time plan unverified.
R122 | 33196737	S	L	H	L	S	H	D1: sequence/concealment incompletely verified. D2/D4 reflect reported masking or protocol safeguards where available. D3 is High unless selected-outcome completion was explicit. D5: prespecified result/time plan unverified.
R123 | 33196744	S	S	H	L	S	H	D1: sequence/concealment incompletely verified. D2/D4 reflect reported masking or protocol safeguards where available. D3 is High unless selected-outcome completion was explicit. D5: prespecified result/time plan unverified.
R124 | 33534905	S	S	H	S	S	H	D1: sequence/concealment incompletely verified. D2/D4 reflect reported masking or protocol safeguards where available. D3 is High unless selected-outcome completion was explicit. D5: prespecified result/time plan unverified.
R125 | 34214220	S	S	H	L	S	H	D1: sequence/concealment incompletely verified. D2/D4 reflect reported masking or protocol safeguards where available. D3 is High unless selected-outcome completion was explicit. D5: prespecified result/time plan unverified.
R126 | 34352146	S	L	L	L	S	S	D1: sequence/concealment incompletely verified. D2/D4 reflect reported masking or protocol safeguards where available. D3 is High unless selected-outcome completion was explicit. D5: prespecified result/time plan unverified.
R127 | 34927342	S	S	H	L	S	H	D1: sequence/concealment incompletely verified. D2/D4 reflect reported masking or protocol safeguards where available. D3 is High unless selected-outcome completion was explicit. D5: prespecified result/time plan unverified.
R128 | 35333199	S	L	H	L	S	H	D1: sequence/concealment incompletely verified. D2/D4 reflect reported masking or protocol safeguards where available. D3 is High unless selected-outcome completion was explicit. D5: prespecified result/time plan unverified.
R129 | 35435087	S	S	H	L	S	H	D1: sequence/concealment incompletely verified. D2/D4 reflect reported masking or protocol safeguards where available. D3 is High unless selected-outcome completion was explicit. D5: prespecified result/time plan unverified.
R130 | 35775872	S	S	H	S	S	H	D1: sequence/concealment incompletely verified. D2/D4 reflect reported masking or protocol safeguards where available. D3 is High unless selected-outcome completion was explicit. D5: prespecified result/time plan unverified.

Result / PMID	D1	D2	D3	D4	D5	Overall	Principal source-specific issues
R131 | 36219518	S	S	H	L	S	H	D1: sequence/concealment incompletely verified. D2/D4 reflect reported masking or protocol safeguards where available. D3 is High unless selected-outcome completion was explicit. D5: prespecified result/time plan unverified.
R132 | 36374507	S	L	H	L	S	H	D1: sequence/concealment incompletely verified. D2/D4 reflect reported masking or protocol safeguards where available. D3 is High unless selected-outcome completion was explicit. D5: prespecified result/time plan unverified.
R133 | 37052609	S	L	H	L	S	H	D1: sequence/concealment incompletely verified. D2/D4 reflect reported masking or protocol safeguards where available. D3 is High unless selected-outcome completion was explicit. D5: prespecified result/time plan unverified.
R134 | 37208833	S	L	H	S	S	H	D1: sequence/concealment incompletely verified. D2/D4 reflect reported masking or protocol safeguards where available. D3 is High unless selected-outcome completion was explicit. D5: prespecified result/time plan unverified.
R135 | 37632290	S	L	H	L	S	H	D1: sequence/concealment incompletely verified. D2/D4 reflect reported masking or protocol safeguards where available. D3 is High unless selected-outcome completion was explicit. D5: prespecified result/time plan unverified.
R136 | 37903326	S	L	H	L	S	H	D1: sequence/concealment incompletely verified. D2/D4 reflect reported masking or protocol safeguards where available. D3 is High unless selected-outcome completion was explicit. D5: prespecified result/time plan unverified.
R137 | 38051121	S	L	H	L	S	H	D1: sequence/concealment incompletely verified. D2/D4 reflect reported masking or protocol safeguards where available. D3 is High unless selected-outcome completion was explicit. D5: prespecified result/time plan unverified.
R138 | 38206151	S	S	H	S	S	H	D1: sequence/concealment incompletely verified. D2/D4 reflect reported masking or protocol safeguards where available. D3 is High unless selected-outcome completion was explicit. D5: prespecified result/time plan unverified.
R139 | 38253883	S	L	H	L	S	H	D1: sequence/concealment incompletely verified. D2/D4 reflect reported masking or protocol safeguards where available. D3 is High unless selected-outcome completion was explicit. D5: prespecified result/time plan unverified.
R140 | 38429946	S	L	H	L	S	H	D1: sequence/concealment incompletely verified. D2/D4 reflect reported masking or protocol safeguards where available. D3 is High unless selected-outcome completion was explicit. D5: prespecified result/time plan unverified.
R141 | 38831064	S	L	H	L	S	H	D1: sequence/concealment incompletely verified. D2/D4 reflect reported masking or protocol safeguards where available. D3 is High unless selected-outcome completion was explicit. D5: prespecified result/time plan unverified.
R142 | 38836757	S	S	L	L	S	S	D1: sequence/concealment incompletely verified. D2/D4 reflect reported masking or protocol safeguards where available. D3 is High unless selected-outcome completion was explicit. D5: prespecified result/time plan unverified.
R143 | 39504084	S	S	H	L	S	H	D1: sequence/concealment incompletely verified. D2/D4 reflect reported masking or protocol safeguards where available. D3 is High unless selected-outcome completion was explicit. D5: prespecified result/time plan unverified.
R144 | 39511699	S	S	H	L	S	H	D1: sequence/concealment incompletely verified. D2/D4 reflect reported masking or protocol safeguards where available. D3 is High unless selected-outcome completion was explicit. D5: prespecified result/time plan unverified.
R145 | 40167803	S	S	H	S	S	H	D1: sequence/concealment incompletely verified. D2/D4 reflect reported masking or protocol safeguards where available. D3 is High unless selected-outcome completion was explicit. D5: prespecified result/time plan unverified.
R146 | 40254943	S	S	H	L	S	H	D1: sequence/concealment incompletely verified. D2/D4 reflect reported masking or protocol safeguards where available. D3 is High unless selected-outcome completion was explicit. D5: prespecified result/time plan unverified.
R147 | 40826297	S	S	H	S	S	H	D1: sequence/concealment incompletely verified. D2/D4 reflect reported masking or protocol safeguards where available. D3 is High unless selected-outcome completion was explicit. D5: prespecified result/time plan unverified.
R148 | 40899732	S	L	H	L	S	H	D1: sequence/concealment incompletely verified. D2/D4 reflect reported masking or protocol safeguards where available. D3 is High unless selected-outcome completion was explicit. D5: prespecified result/time plan unverified.
R149 | 41129279	S	L	H	L	S	H	D1: sequence/concealment incompletely verified. D2/D4 reflect reported masking or protocol safeguards where available. D3 is High unless selected-outcome completion was explicit. D5: prespecified result/time plan unverified.
R150 | 41168367	S	L	H	L	S	H	D1: sequence/concealment incompletely verified. D2/D4 reflect reported masking or protocol safeguards where available. D3 is High unless selected-outcome completion was explicit. D5: prespecified result/time plan unverified.
R151 | 41650338	S	S	H	S	S	H	D1: sequence/concealment incompletely verified. D2/D4 reflect reported masking or protocol safeguards where available. D3 is High unless selected-outcome completion was explicit. D5: prespecified result/time plan unverified.

Result / PMID	D1	D2	D3	D4	D5	Overall	Principal source-specific issues
R152 | 41731228	S	L	H	L	S	H	D1: sequence/concealment incompletely verified. D2/D4 reflect reported masking or protocol safeguards where available. D3 is High unless selected-outcome completion was explicit. D5: prespecified result/time plan unverified.
R153 | 42151612	S	S	H	L	S	H	D1: sequence/concealment incompletely verified. D2/D4 reflect reported masking or protocol safeguards where available. D3 is High unless selected-outcome completion was explicit. D5: prespecified result/time plan unverified.
R154 | 42300963	S	S	H	L	S	H	D1: sequence/concealment incompletely verified. D2/D4 reflect reported masking or protocol safeguards where available. D3 is High unless selected-outcome completion was explicit. D5: prespecified result/time plan unverified.
R155 | 42424535	S	L	H	L	S	H	D1: sequence/concealment incompletely verified. D2/D4 reflect reported masking or protocol safeguards where available. D3 is High unless selected-outcome completion was explicit. D5: prespecified result/time plan unverified.
R156 | 42635327	S	L	H	L	S	H	D1: sequence/concealment incompletely verified. D2/D4 reflect reported masking or protocol safeguards where available. D3 is High unless selected-outcome completion was explicit. D5: prespecified result/time plan unverified.
R157 | 42869969	S	S	H	S	S	H	D1: sequence/concealment incompletely verified. D2/D4 reflect reported masking or protocol safeguards where available. D3 is High unless selected-outcome completion was explicit. D5: prespecified result/time plan unverified.
R158 | 26659938	S	S	H	L	S	H	D1: sequence/concealment incompletely verified. D2/D4 reflect reported masking or protocol safeguards where available. D3 is High unless selected-outcome completion was explicit. D5: prespecified result/time plan unverified.
R159 | 33792961	S	L	H	L	S	H	D1: sequence/concealment incompletely verified. D2/D4 reflect reported masking or protocol safeguards where available. D3 is High unless selected-outcome completion was explicit. D5: prespecified result/time plan unverified.

Domain totals were: D1, 21 Low and 138 Some concerns; D2, 72 Low and 87 Some concerns; D3, 47 Low and 112 High; D4, 100 Low, 41 Some concerns, and 18 High; and D5, one Low and 158 Some concerns. Overall judgments were 123 High and 36 Some concerns. These ratings describe methodological concerns in specified results and are not efficacy estimates or certainty grades.

NI = No information; NA = Not applicable. Companion S2B and the signaling file form part of the assessment record. Incomplete reporting was not treated as evidence that a method was either adequate or defective; it was handled according to the corresponding RoB 2 signaling pathway.
