# Supplementary material for: Biomodulators and Skin Boosters in Skin Rejuvenation: Mechanisms, Applications, and Future Perspectives
Source: Int J Mol Sci. 2026 Oct 28;27(21):9647. doi: 10.3390/ijms27219647 (PMC13762510; doi:10.3390/ijms27219647)
Supplement: Supplementary file 1 [file ijms-27-09647-s001.zip › Supplementary_Table_S3_Final.pdf]

Supplementary Table S3 Clinical comparisons

This table contains 159 comparative reports, each contributing one retrospectively specified comparison, outcome, and assessment time. Ninety-four reports were incorporated after the completed 195-record access and eligibility recheck. Eight contextual reports, three noncomparative primary estimates, eight retrieved reports with unresolved design or adult-eligibility questions, and four access-unresolved reports are listed separately and are not counted among the 159 comparative results. Report counts are not assumed to represent independent trials.

Abbreviations: HA, hyaluronic acid; MN, microneedling; FCO2, fractional CO2 laser; RF/FMRF, radiofrequency/fractional microneedle RF; BoNT-A/BTA, botulinum toxin A; PRP/PRF, platelet-rich plasma/fibrin; PN, polynucleotides; EV, extracellular vesicles; EMNV, exosome-mimetic nanovesicles; CM, conditioned medium; GAIS, Global Aesthetic Improvement Scale. Scale names are retained or abbreviated consistently in the endpoint column.

Report / PMID	Assigned regimen	Selected endpoint and time	Finding and interpretation
Baspeyras 2013 23715889	Three monthly split-cheek HA–glycerol–mannitol injections vs saline	Masked expert-panel skin radiance at 3 months after the last treatment (secondary outcome)	Radiance favoured the formulation (p=0.012); the primary elasticity contrast was null.
Lee 2014 25324650	MN plus embryonic stem-cell-derived endothelial precursor conditioned medium vs matched MN plus saline	Masked photographic wrinkle improvement 2 weeks after the fifth session	Photographic scores favoured conditioned medium (1.92 vs 1.49); no isolated EV effect is established.
Faghihi 2016 26924405	FCO2 plus intradermal PRP vs the same laser plus saline, randomized facial sides	Two blinded dermatologists' quartile acne-scar improvement grade, four months after the second session	The final scar comparison was null (p=0.23); erythema/edema were worse or longer with PRP.
Hui 2017 27222038	FCO2 plus PRP injection/application vs matched laser plus saline on the contralateral facial side	VISIA wrinkle score at 3 months after treatment	Wrinkle change was null (p=0.145), despite favourable texture/elasticity and subjective measures.
Porwal 2018 30210162	Dermaroller plus PRP vs dermaroller alone, parallel participant groups	GB quantitative scar score, one month after the final session	Scar improvement favoured the combination; incomplete follow-up and unverified assessor masking limit confidence.
Alam 2018 30419125	Single split-cheek PRP injection vs equal-volume saline	Masked dermatologist fine-line photoaging score at 2 weeks	Masked photoaging ratings were null; favourable six-month patient reports are separate secondary outcomes.
El-Taieb 2019 31420849	PRP plus Er:YAG laser vs Er:YAG laser alone, two of three randomized groups	Blinded photographic quartile clinical-improvement grade at the post-treatment assessment following the 12-session course	A PRP adjunct comparison against active laser, not PRP monotherapy; final calendar timing remains unclear.
An 2019 33911590	HA/AHP-8 dissolving microneedle patch vs the same HA patch without AHP-8, randomized participant groups	Antera 3D fine-to-moderate periocular wrinkle change at Day 29	Tests added AHP-8 in an HA microneedle patch, not injectable HA; distinguish group from within-group change.
Wu 2020 31769596	Stabilized HA gel vs no treatment on the contralateral dorsal hand	MoisturemeterEpiD change in tissue water content at Month 3	Tissue-water change favoured HA; hydration does not establish collagen regeneration or durable global rejuvenation.
Kwon 2020 33073298	Three FCO2 sessions plus adipose-cell-derived exosome gel vs the same laser plus control gel	Percentage reduction in total ECCA acne-scar score at Week 12	ECCA reduction was 32.5% vs 19.9% (p<0.01); the estimate concerns the complete gel plus laser.
Araco 2021 33755110	PN-HPT 4 mL twice, three weeks apart, vs the same volume of saline; parallel groups	Antera 3D skin texture at 3 months after the second treatment	Texture favoured PN-HPT at three months (22.75 vs 35.8); inconsistent satisfaction scales and follow-up captions require caution.

Report / PMID	Assigned regimen	Selected endpoint and time	Finding and interpretation
Li 2022<br>34758056	HA compound filling plus mesotherapy vs BoNT-A, parallel groups	Blinded adapted ATNLS at 6 months after final treatment	A combined filling/mesotherapy regimen versus active toxin; neither single-ingredient benefit nor superiority over no treatment is isolated.
Yogya 2022<br>35501660	Three matched noninsulated RF sessions plus a PN–HA–amino acid/vitamin/peptide serum vs RF plus saline	Maximum periorbital wrinkle depth at 6 months after the final session	Maximum depth was null at all visits; indentation favoured serum at two months. Multiple ingredients prevent PN-only attribution.
Liang 2022<br>35685406	MN plus umbilical-cord MSC conditioned medium vs matched MN plus saline	Masked physician global assessment of skin texture 2 weeks after the fifth treatment	Short-term masked texture ratings favoured conditioned medium plus MN; neither standalone nor exosome-specific efficacy is established.
Siperstein 2022<br>35689521	Split-face VYC-17.5L HA for atrophic scars vs saline, before Day 120 crossover	Masked live-evaluator change in QGSGS 90 days after the last treatment	QGSGS reduction was 6.6 vs 1.7 (p=0.0008); reported degrees of freedom conflict with pairing. Later crossover is not independent evidence.
Kaufman-Janette 2022<br>36129233	RHA Redensity with mepivacaine vs the same HA filler with lidocaine, randomized perioral sides	Treating-investigator Perioral Rhytid Severity Rating Scale at Day 30	One-month wrinkle changes were nearly identical; the anesthetic-formulation comparison does not establish HA benefit over no treatment.
Abdelwahab 2022<br>36564573	Subcision plus HA filler vs subcision plus FCO2, randomized facial-side contrast	Two blinded investigators' quartile photographic improvement grade 6 months after treatment	HA versus laser was null (p=0.171); participant/site counts and paired analysis remain unclear.
Diab 2023<br>36520210	Fluid PRF vs PRP injection on the left facial side, parallel randomized participant groups	GB global scar score four weeks after the last session	Only participant-level PRF versus PRP assignment is randomized; fixed-side needling comparisons are separate.
Yusharyahya 2023<br>36798539	MN + ADMSC secretome vs FCO2 + the same secretome	DPAS photoaging score, Week 6	Both protocols include secretome; the contrast compares delivery procedures and cannot isolate adding secretome.
Duteil 2023<br>36909864	M-HA10 non-crosslinked HA vs saline, split face	Instrumental crow's-feet depth, Day 53, baseline-depth class 110–1000 micrometres	Instrumental depth improved, but the clinical crow's-feet contrast was null (p=0.071); follow-up ended ten days after session three.
Chiu 2023<br>37074002	VYC-12L vs delayed no-treatment control; PRO companion report	FACE-Q Satisfaction With Skin, Month 1 control-period comparison	Participant satisfaction is a companion outcome of the Alexiades 2023 report (PMID 37163665); unblinded reports require a separate measurement-risk judgment.
Alexiades 2023<br>37163665	VYC-12L intradermal cheek treatment vs delayed treatment before crossover	Masked ACSS bilateral responder rate at Month 1	Month 1 ACSS response was 57.9% vs 4.5% using imputation; later crossover removes the original untreated comparison.
Park 2023<br>37377400	Human adipose stem-cell exosome-containing composite solution plus MN vs saline plus identical MN	Two-dermatologist GAIS assessment, Week 12	Week 12 GAIS favoured the composite (p=0.005); coingredients and shared MN preclude purified-exosome attribution.
Fanian 2023<br>37577796	NCTF 135 HA composite injections plus cream vs the same cream alone	LifeViz 3D crow's-feet mean wrinkle volume, Day 75	Day 75 wrinkle volume favoured injections plus cream (p=0.0005); elasticity benefit was not consistent.
Płatkowska 2023<br>37631060	Bipolar radiofrequency plus HA/CaHA/amino-acid mesotherapy vs matched radiofrequency alone	TEWL at the 28-day visit	Descriptive TEWL changes lack sufficient variance for a precise contrast; the formulation also contains CaHA and amino acids.
Faria 2023<br>37823019	Premixed botulinum toxin plus diluted CaHA vs diluted CaHA alone, participant groups	Reported Month 3 GAIS distribution in Table 2	A small active-comparator study of a combined regimen; tabled GAIS assessor identity is unclear.

Report / PMID	Assigned regimen	Selected endpoint and time	Finding and interpretation
Bano 2023<br>38314374	MN plus 1% noncrosslinked HA vs MN alone, parallel groups	GSGS score at the final follow-up after three sessions	Some scar measures favour the combination; unblinded grading and short follow-up restrict interpretation.
Behrangi 2024<br>38174839	Cross-linked HA plus BTA in one session vs noncrosslinked HA plus BTA in two sessions	GB qualitative scar grade at 6 months from baseline	HA crosslinking and session number both differ; shared BTA and small groups prevent single-component attribution.
Pincelli 2024<br>38798929	PRP injection plus topical platelet-poor plasma and MN vs saline injection/dressing plus matched MN	Clinical skin roughness change at Week 24	Clinical roughness, laxity and rhytids did not favour the PRP-containing protocol; this is the facial trial.
V 2024<br>38916005	PRP injections plus MN vs PRP injections alone, parallel groups	GB quantitative scar score one month after the third session	Both groups receive PRP; the contrast tests adding MN. Masking claims need caution for visibly different procedures.
Mohamed 2024<br>39122500	MN plus noncrosslinked HA vs MN plus topical insulin, randomized facial sides	GB quantitative scar score 3 months after the final session	The final scar-score contrast was null (p=0.348); baseline improvement does not establish added HA benefit.
Arruda 2024<br>39231078	PLLA vs saline, parallel groups (7 vs 3)	Blinded investigator-rated skin radiance, 18 weeks after third treatment	Clinical facial ratings and postauricular biopsies concern different sites; histology does not establish patient-important rejuvenation.
Boca 2024<br>39356623	NCTF 135HA delivered by Nanosoft microneedle vs classic needle, randomized facial sides	Clinical skin-radiance grade at Day 75, thirty days after the third treatment	Both sides receive NCTF 135HA; the contrast compares delivery devices, not adding the solution.
Hong 2025<br>39370844	PN delivered by needle-free jet vs conventional intradermal needle, randomized facial sides	Two-dermatologist GAIS at 2 weeks	Selected ratings/pain favour jet delivery; both sides receive PN, without a PN-free comparator.
Li 2025<br>39503284	PRP injection vs MN + topical PRP vs RF + injected PRP	Blinded Lemperle neck-wrinkle score, Month 6	PRP is present in all groups; only delivery/procedure protocols are compared.
Vachiramon 2025<br>39852991	Onabotulinumtoxin A plus HA filler vs the same toxin alone, randomized facial sides	Antera 3D skin-roughness comparison at Month 1	The combination produced greater Month 1 roughness than toxin alone; favourable other endpoints do not erase this signal.
Ni 2025<br>40296541	Acupotomy subcision plus HA vs acupotomy subcision alone, two randomized groups	ATNLS 6 months after treatment	Tests HA added to subcision; clinical neck grades and imaging-derived collagen proxies remain distinct.
Rutnumnoi 2025<br>40304039	CPM-HA with glycerol vs CPM-HA without glycerol, randomized facial sides	Blinded dermatologist visual pore grade at Week 32	Week 32 grade distributions were null (p=1.000), although a longitudinal model favoured glycerol; both sides receive HA.
Zhou 2025<br>40476635	FCO2 plus PLLA injection vs FCO2 alone	QGSGS at 6 months after initial laser treatment	Post-treatment GSS favoured the combination (p=0.007); missingness and unverified assessor masking limit confidence.
Rosso 2025<br>40509910	Sequential RHA1 plus R1 vs RHA1 alone, exploratory smiling-line subpopulation comparison	Blinded-evaluator GAIS improvement at Month 5	The primary RHA1 response precedes randomization; only exploratory additional-R1 comparisons qualify, with small controls and unclear subgroup denominators.
Wang 2025<br>40599706	High-dose cross-linked HA (48 mg) vs low-dose HA (24 mg) on randomized facial sides	Blinded Alexiades photoaging score 6 months after the third treatment	A dose comparison between active HA exposures; it does not establish HA efficacy versus no treatment.
Ahramiyanpour 2025<br>40665746	Endo-radiofrequency subcision plus PRP vs the same subcision alone, randomized facial sides	GB quantitative scar score at 3 months	No significant between-protocol advantage was reported; within-side change does not establish added PRP benefit.

Report / PMID	Assigned regimen	Selected endpoint and time	Finding and interpretation
Neves 2025 40814769	Bilateral onabotulinumtoxinA followed after 1 month by unilateral VYC-12/Volite HA vs contralateral simulated cannula injection	Primary orbicularis oculi EMG activity across follow-up to Month 6	Primary EMG was null (p=0.86); favourable secondary wrinkle ratings used an unpaired test despite paired assignment.
Zare 2025 40853048	Cultured autologous fibroblasts plus PRP vs PRP alone, randomized facial sides	VisioFace acne-scar volume percentage change at 6 months	Scar volume favoured adding cultured cells (p=0.043); the contrast does not isolate PRP efficacy.
Seshagiri 2025 41585978	Dermaroller plus intradermal PRP with vs without topical vitamin C, randomized facial sides	Observer Global Improvement Score on postoperative Day 20	Day 20 clinical ratings favoured added vitamin C; unpaired testing of paired sides limits inference and durability is unknown.
Kupwiwat 2026 41442012	FMRF followed by topical PLLA suspension vs the same procedure followed by sterile water, randomized facial halves	Antera 3D skin texture change at 6 months after the second session	Texture favoured topical PLLA after FMRF (MD −1.18; 95% CI −2.33 to −0.03); no injectable-PLLA monotherapy effect is established.
Faria 2026 41669823	CaHA + polymicronutrient diluent vs CaHA + saline, split hand	Three blinded investigators' Hand Grading Scale, Day 90	Hydration was null (p=0.294); in vitro gene expression cannot substitute for the selected clinical HGS outcome.
Lee 2026 41828422	Intradermal particulated human acellular dermal matrix plus HA blend vs HA alone, randomized facial sides	Investigator ACSS at Week 20	Cheek smoothness favoured the blend; test/control HA doses differ (7.5 vs 20 mg), preventing isolated matrix attribution.
Jiang 2026 41873431	NCTF 135 HA vs HEARTY (non-inferiority)	Independent-evaluator ATNLS responder rate, Week 4 after final injection	Noninferiority to an active composite does not establish benefit over no treatment; later response decline limits durability claims.
Cheng 2026 41971651	Three intradermal sessions of noncrosslinked HA vs routine skincare without injection, parallel participant groups	Photographic ASRS distribution at Day 84	Approximately 87% of treated participants reached ASRS grades 0–2 at Day 84; baseline and randomized totals contain inconsistencies.
Saffarian 2026 42063688	Botulinum toxin A followed by two Profhilo HA sessions vs BoNT-A alone, parallel groups	Blinded dermatologist frontal Merz score at rest 3 months after the last treatment	Resting Merz contrasts were null at three months (raters p=0.775/0.549); within-arm change does not establish added HA benefit.
Munoz-Lora 2026 42087429	Sculptra vs Elleva; both facial sides treated in each participant	Ultrasound dermal-thickness comparison between formulations, Day 120	Only product assignment is randomized; fixed injection planes switched at Day 60 and cannot estimate randomized plane effects.
Bravo 2026 42181534	Rennova Elleva vs Sculptra	Physician GAIS at Day 120, between-product split-arm contrast	Unpaired tests were used for paired arms; nonsignificance does not establish product equivalence.
Wanitphakdeedecha 2026 42225690	Intradermal photothermal biomodulated PRP vs standard PRP, randomized facial sides	Antera periorbital wrinkle maximum-depth treatment contrast across follow-up through six months	Periorbital wrinkle-depth treatment effect: p=0.038; several roughness, pore, pigment and biomechanical contrasts were null.
Joseph 2026 42235050	PLLA-SCA reconstituted in 8 vs 17 mL sterile water (+ lidocaine)	Treating-investigator GDS responder rate, Month 9	Both groups receive PLLA; within-group response is not placebo-controlled efficacy. One treatment-related nodule resolved after ten months.
Yang 2026 42367716	Three monthly HA composite injections vs no treatment	Blinded GAIS responder rate, 1 month after final treatment (V4)	A complete HA/amino-acid formulation versus no treatment; ingredient-specific benefit is not isolated.
Chen 2026 42410192	FCO2 followed by CaHA powder compounded with noncrosslinked HA vs laser alone, parallel groups	Blinded quantitative global acne-scarring score change at 6 months after laser	Scar-score improvement was 10.61 vs 5.74 (p<0.001); compounded CaHA–HA plus laser does not isolate pure CaHA efficacy.

Report / PMID	Assigned regimen	Selected endpoint and time	Finding and interpretation
Shishido 2026 42420660	AbobotulinumtoxinA plus Restylane Skinbooster Vital vs the same toxin with simulated cannula treatment, randomized facial sides	Stereophotogrammetric periorbital skin-texture score at 6 months after toxin	Texture favoured added HA at six months (p=0.002), while primary EMG was null; distinct from the onabotulinumtoxinA trial by Neves 2025 (PMID 40814769).
Helmi 2026 42621380	FCO2 plus topical PN and exosome-mimetic nanovesicles vs laser alone, parallel groups	ECCA acne-scar score change 3 months after the last treatment	ECCA reduction was 47.3% vs 35.9% (p<0.001); the combined topical PN/EMNV adjunct does not establish isolated or injectable EV efficacy.
Moradi 2026 42847981	Three SB-NASHA décolletage injections vs no treatment, randomized 3:1	Blinded-evaluator GDS responder rate at Week 12	GDS response was 73.6% vs 18.8%; only 192 participants were randomized. Nineteen additional participants contributed nonrandomized safety observations.
Sundaram 2022 34608092	RHA Redensity vs no treatment; parallel groups (150 vs 52 randomized)	Masked perioral-rhytid response, Week 8 before crossover	Response was 80.7% vs 7.8% (p<0.0001); mITT denominators 150/51 with LOCF. Later crossover removes the untreated comparison.
Song 2023 36892373	HA mesotherapy vs dextran/dextrose preparation; 17/group	Physician global photoaging improvement, Day 84	Improvement was 53% vs 76%, without a significant difference (P>0.05). Before–after device changes do not establish superiority; naming/statistical inconsistencies require caution.
Murillo Martin 2023 37594172	Deep vs superficial HA injection; paired adults	Independent-observer crow's-feet score, Week 7	Scores were 1.83 vs 1.87 (p=0.317). Both sides receive HA; this compares injection protocols. Primary hierarchy and observed denominators remain unverified.
Wong 2023 37833618	MN plus multiple-active formulation/aftercare vs MN plus HA serum/conventional aftercare	Masked tactile texture, Day 28	Change was −32.4% vs −25.7% (p=0.0025); 32/33 entered per-protocol analysis. Different aftercare is part of the assigned package, preventing ingredient attribution.
Gentile 2026 42339013	Fibroblast-containing micrografts vs NCTF-135HA; 20/group	Physician WSRS improvement, Month 12 (reported primary)	Improvement was 1.4 vs 0.3 points (p<0.01). Two active regimens; no untreated arm or reported contrast CI. Unblinded subjective scoring limits confidence.
Carruthers et al., 2003 | 12859378	This was a prospective randomized study of 38 subjects with moderate to severe glabellar rhytides. Half of the subjects were treated with BTX-A and NASHA and the other half with NASHA alone.	By comparison with the NASHA-alone group, the BTX-A plus NASHA group showed a better response both at rest and on maximum frown, and this response was maintained for longer.	By comparison with the NASHA-alone group, the BTX-A plus NASHA group showed a better response both at rest and on maximum frown, and this response was maintained for longer. The median time for return to preinjection furrow status occurred at 18 weeks in the NASHA-alone group compared with 32 weeks for the BTX-A plus NASHA group. This result applies to the complete assigned regimen and does not establish a class-wide effect.
Kono et al., 2008 | 18547178	Randomized, evaluator-blind, split-face comparison study of single cross-linked versus double cross-linked hyaluronic acid in the treatment of glabellar lines.	Selected clinical or instrumental skin outcome at the final common assessment reported in the accessible source.	The accessible report identified a randomized comparison but did not provide sufficiently detailed result text in the indexed record; interpretation is therefore conservative. This result applies to the complete assigned regimen and does not establish a class-wide effect.
Williams et al., 2009 | 19735521	The intra-individual comparison in 15 volunteers involved injection sessions at week 0, 4, and 8 with random assignment of left and right hand to either S-HA or NS-HA.	Intradermal injection of S-HA generated significant improvement in skin elasticity and surface roughness at week 12 compared to baseline.	Intradermal injection of S-HA generated significant improvement in skin elasticity and surface roughness at week 12 compared to baseline. On the hands treated with NS-HA, there was a trend for improvement (not significant). This result applies to the complete assigned regimen and does not establish a class-wide effect.
Carruthers et al., 2010 | 21070457	Ninety female participants aged 35 to 55 were randomized to one of three groups: 24-mg/mL cohesive gel alone (n=30), onabotulinumtoxinA alone (n=30), or the combination (n=30).	All treatments resulted in significant improvements from baseline at all end points and on all PRO measures.	All treatments resulted in significant improvements from baseline at all end points and on all PRO measures. For all measures and most time points, the 24-mg/mL cohesive gel treatment groups experienced greater improvements than onabotulinumtoxinA alone. This result applies to the complete assigned regimen and does not establish a class-wide effect.
Carruthers et al., 2010 | 21134044	Ninety female participants aged 35 to 55 were randomized to one of three groups: 24-mg/mL cohesive gel alone (n=30), onabotulinumtoxinA alone (n=30), or the combination (n=30).	For all end points and most time points, subjects treated with onabotulinumtoxinA plus the 24-mg/mL cohesive gel had greater improvement from baseline than subjects treated with onabotulinumtoxinA or the 24-mg/mL cohesive gel filler alone.	For all end points and most time points, subjects treated with onabotulinumtoxinA plus the 24-mg/mL cohesive gel had greater improvement from baseline than subjects treated with onabotulinumtoxinA or the 24-mg/mL cohesive gel filler alone. This result applies to the complete assigned regimen and does not establish a class-wide effect.

Report / PMID	Assigned regimen	Selected endpoint and time	Finding and interpretation
Lee et al., 2011 | 21635618	All participants received one session of ablative CO(2) fractional resurfacing. Immediately after resurfacing, facial halves were randomly assigned to receive treatment with autologous PRP injections on one side (experimental side) and normal saline injections on the other side (control side).	All participants completed the study.	All participants completed the study. Erythema on the experimental side improved faster than on the control side and was significantly less at day 4 (p=.01). This result applies to the complete assigned regimen and does not establish a class-wide effect.
Shin et al., 2012 | 22288389	Twenty-two Korean women underwent three sessions of fractional laser; 11 were treated with topical application of PRP combined with fractional laser.	PRP combined with fractional laser increased subject satisfaction and skin elasticity and decreased the erythema index.	PRP combined with fractional laser increased subject satisfaction and skin elasticity and decreased the erythema index. PRP increased the length of the dermoepidermal junction, the amount of collagen, and the number of fibroblasts. This result applies to the complete assigned regimen and does not establish a class-wide effect.
Royo de la Torre et al., 2013 | 23545926	We performed a comparative, parallel-group, double-blind trial with an external observer (blinded to the type of treatment administered).	Pain: The severity of pain was decreased in patients treated with HA+L on application (P <.001) and 10 minutes later (P=.008).	Pain: The severity of pain was decreased in patients treated with HA+L on application (P <.001) and 10 minutes later (P=.008). Efficacy and satisfaction: No significant differences existed between the 2 groups at months 3, 9, and 12. This result applies to the complete assigned regimen and does not establish a class-wide effect.
Lim et al., 2014 | 24245880	Subjects were randomized to be injected a stabilized hyaluronic acid-based gel of nonanimal origin (NAHSA injector, Restylane Vital®, Q-med) on one side of nasojugal groove, with the other side paired as control.	All patients reported a high degree of satisfaction.	All patients reported a high degree of satisfaction. Duration of overall effect varied among the patients. This result applies to the complete assigned regimen and does not establish a class-wide effect.
Dubina et al., 2013 | 24305424	Split-face, randomized control trial. Subjects were randomized and injected with botulinum toxin alone on one side of the forehead/glabella and hyaluronic acid filler followed by botulinum toxin on the other side.	Ratings of both treatments showed comparable efficacy, with 100% of subjects experiencing improvement from baseline regardless of treatment type at weeks 2, 6, and 12.	Ratings of both treatments showed comparable efficacy, with 100% of subjects experiencing improvement from baseline regardless of treatment type at weeks 2, 6, and 12. Combination treatment produced longer-lasting results in dynamic forehead wrinkles, and greater static and dynamic wrinkle reduction in the glabella at week 24. This result applies to the complete assigned regimen and does not establish a class-wide effect.
Gawdat et al., 2014 | 24354616	Thirty patients were randomly divided into two groups. Group 1 was administered FCL followed by ID PRP on one side and FCL followed by ID saline on the other.	Combined PRP- and FCL-treated areas had a significantly better response (p = .03), fewer side effects, and shorter downtime (p = .02) than FCL-treated areas, but there were no significant differences in ID- and topical PRP-treated areas in degree of response…	Combined PRP- and FCL-treated areas had a significantly better response (p = .03), fewer side effects, and shorter downtime (p = .02) than FCL-treated areas, but there were no significant differences in ID- and topical PRP-treated areas in degree of response and downtime (p = .10); topically treated areas had significantly lower pain scores. This result applies to the complete assigned regimen and does not establish a class-wide effect.
Kang et al., 2014 | 24566300	Three split-face PRP treatment sessions over 12 weeks versus the matched control side.	Infraorbital wrinkle and skin-tone assessment after the treatment course.	The report favored PRP for selected wrinkle and skin-tone outcomes; the short preliminary report and limited methodological detail restrict inference. This result applies to the complete assigned regimen and does not establish a class-wide effect.
Nofal et al., 2014 | 25006854	Forty-five patients with atrophic acne scars were randomly assigned to 3 equal groups; Group A received intradermal injection of PRP, Group B received chemical reconstruction of skin scars technique with TCA 100%, and Group C was treated by combined skin needling and PRP.	All the patients completed the study.	All the patients completed the study. The 3 groups showed statistically highly significant improvement in the degree of acne scars after treatment (p < .001). This result applies to the complete assigned regimen and does not establish a class-wide effect.
Tedeschi et al., 2015 | 25539986	Mesotherapy with an Intradermal Hyaluronic Acid Formulation for Skin Rejuvenation: An Intrapatient, Placebo-Controlled, Long-Term Trial Using High-Frequency Ultrasound.	Selected clinical or instrumental skin outcome at the final common assessment reported in the accessible source.	The accessible report identified a randomized comparison but did not provide sufficiently detailed result text in the indexed record; interpretation is therefore conservative. This result applies to the complete assigned regimen and does not establish a class-wide effect.
Beer et al., 2015 | 25828037	A randomized, evaluator-blinded, controlled study of effectiveness and safety of small particle hyaluronic acid plus lidocaine for lip augmentation and perioral rhytides.	Statistically significantly more patients were treatment successes with SPHAL (upper lip [80.2% vs 11.9%], lower lip [84.2% vs 18.4%], and upper and lower lips combined [76.1% vs 11.6%]), compared with no treatment (p <.001, all outcomes).	Statistically significantly more patients were treatment successes with SPHAL (upper lip [80.2% vs 11.9%], lower lip [84.2% vs 18.4%], and upper and lower lips combined [76.1% vs 11.6%]), compared with no treatment (p <.001, all outcomes). Patients treated for both lip augmentation and perioral rhytides were rated as having an aesthetically meaningful improvement in perioral rhytides (p <.001). This result applies to the complete assigned regimen and does not establish a class-wide effect.
Butterwick et al., 2015 | 26606435	Men and women aged 35 years or older with moderate-to-severe perioral lines were recruited for this randomized controlled, rater-blinded, 2-arm trial.	A total of 136 subjects received treatment and 132 completed the trial (mean age: 58 ± 8 years).	A total of 136 subjects received treatment and 132 completed the trial (mean age: 58 ± 8 years). Total volume injected was 1.18 mL (HYC-24L) and 1.32 mL (CPM-22.5). This result applies to the complete assigned regimen and does not establish a class-wide effect.

Report / PMID	Assigned regimen	Selected endpoint and time	Finding and interpretation
Dayan et al., 2015 | 26618456	This ongoing, multicenter, single-blind study randomized 213 subjects to the treatment group (n = 157) or concurrent no-treatment control group that received delayed treatment (n = 56). To meet this endpoint, the treatment group had to have a responder rate ≥60% and significantly greater than the treatment control group at Month 3.	The primary endpoint was met, with a 79.1% responder rate for the treatment group and 26.1% for the treatment control group ($p < 0.0001$).	The primary endpoint was met, with a 79.1% responder rate for the treatment group and 26.1% for the treatment control group ($p < 0.0001$). More than half of subjects (56.4%) maintained treatment response for 12 months. This result applies to the complete assigned regimen and does not establish a class-wide effect.
Zhou et al., 2016 | 26735291	Twenty-two subjects were enrolled in the study and divided into two groups. Nine subjects were included in skin rejuvenation group and thirteen subjects were included in acne scar group, and all subjects underwent three sessions of FxCR.	ADSC-CM combined with FxCR increased subject satisfaction, elasticity, skin hydration, and skin elasticity and decreased TEWL, roughness, and the melanin index in both acne scars and skin rejuvenation groups.	ADSC-CM combined with FxCR increased subject satisfaction, elasticity, skin hydration, and skin elasticity and decreased TEWL, roughness, and the melanin index in both acne scars and skin rejuvenation groups. Histologic analysis showed that ADSC-CM increased dermal collagen density, elastin density, and arranged them in order. This result applies to the complete assigned regimen and does not establish a class-wide effect.
Roh et al., 2016 | 26910661	Each participant submitted to a single treatment with a nonanimal stabilized hyaluronic acid injection to one side of the lower cheek.	Stratum corneum hydration was significantly improved after injection.	Stratum corneum hydration was significantly improved after injection. Although no significant improvement was observed at 1 week after treatment, the Corneometer readings for the treated side were significantly higher than those for the untreated the side after the 2-, 4-, 8-, and 12-week treatment visits. This result applies to the complete assigned regimen and does not establish a class-wide effect.
Choi et al., 2017 | 27503619	HA or placebo delivered by needleless microjet or multineedle injection in four randomized groups.	Wrinkle Severity Rating Scale and dermal-thickness assessments after treatment.	Within-group HA changes were reported, while the available report did not establish a clear between-treatment superiority; delivery method and active preparation must be interpreted separately. This result applies to the complete assigned regimen and does not establish a class-wide effect.
Geronemus et al., 2017 | 28157728	This prospective multicenter study randomized 225 adults with minimal, mild, or moderate lip fullness on the validated 5-point Allergan Lip Fullness Scale (LFS) to treatment with VYC-15L or NASHA; optional touch-up was administered 30 days later. The primary effectiveness end point was noninferiority of VYC-15L versus NASHA by mean change in LFS score from…	The primary end point was met.	The primary end point was met. At Month 3, LFS responder rates were 80.3% for VYC-15L versus 70.8% for NASHA. This result applies to the complete assigned regimen and does not establish a class-wide effect.
Gawdat et al., 2017 | 28382785	They underwent a split face therapy where each side was randomly assigned to treatment by either readymade growth factors (area A) or autologous PRP (area B). All patients received six sessions at 2-weeks interval.	Both procedures yielded significant improvement regarding both GAIS (skin turgor and overall vitality) and OCT (epidermal and dermal thickness) assessment.	Both procedures yielded significant improvement regarding both GAIS (skin turgor and overall vitality) and OCT (epidermal and dermal thickness) assessment. Significant negative correlation was detected between patients' age, sun exposure, and GAIS. This result applies to the complete assigned regimen and does not establish a class-wide effect.
Ibrahim et al., 2017 | 28504480	This study included 90 patients with atrophic scars and were classified randomly into three groups: I: 28 patients treated with microneedling, one session every 4 weeks; II: 34 patients treated with intradermal injection of platelet-rich plasma, one session every 2 weeks; and III: 28 patients treated with alternative sessions of each microneedling and…	There was a statistically significant improvement in the appearance of atrophic scars, with reduction in the scores associated with the clinical evaluation scale for atrophic scarring in all groups, but the improvement was more obvious in group III.	There was a statistically significant improvement in the appearance of atrophic scars, with reduction in the scores associated with the clinical evaluation scale for atrophic scarring in all groups, but the improvement was more obvious in group III. This result applies to the complete assigned regimen and does not establish a class-wide effect.
Trevidic et al., 2017 | 28595250	Prospective, randomized, rater- and patient-blind, split-face comparison of AFU with JUVEDERM Ultra 3 (JUV) and AFFL with FIRST LINES PureSense (FLPS).	At D30 and D180 respectively, 61 and 57 patients were assessed.	At D30 and D180 respectively, 61 and 57 patients were assessed. Scores for nasolabial folds and crow's feet showed statistically significant improvements at D30, D90, and D180. This result applies to the complete assigned regimen and does not establish a class-wide effect.
Choi et al., 2017 | 28892233	Hyaluronic acid microneedle patch for the improvement of crow's feet wrinkles.	Selected clinical or instrumental skin outcome at the final common assessment reported in the accessible source.	The accessible report identified a randomized comparison but did not provide sufficiently detailed result text in the indexed record; interpretation is therefore conservative. This result applies to the complete assigned regimen and does not establish a class-wide effect.
Hong et al., 2018 | 28987023	The subjects were divided into 3 groups: (1) soluble hyaluronic acid (HA) microneedle patch alone, (2) soluble HA microneedle patch plus adenosine wrinkle cream, and (3) adenosine wrinkle cream alone.	Combination treatment with wrinkle cream and microneedle patch significantly improved Merz scale for crow's feet and nasolabial folds, compared to the sole application of wrinkle cream or patch.	Combination treatment with wrinkle cream and microneedle patch significantly improved Merz scale for crow's feet and nasolabial folds, compared to the sole application of wrinkle cream or patch. Measurement on the crow's feet showed an overall improvement in all 3 groups, yielding no significant differences among the groups. This result applies to the complete assigned regimen and does not establish a class-wide effect.
Willemsen et al., 2018 | 29019860	The study conducted was a single-center, double-blind, placebo-controlled, randomized trial (2012 to 2015).	Platelet-rich plasma did not improve the outcome of facial lipofilling when looking at skin elasticity improvement, graft volume maintenance in the nasolabial fold.	Platelet-rich plasma did not improve the outcome of facial lipofilling when looking at skin elasticity improvement, graft volume maintenance in the nasolabial fold. Reversal of the correlation between age and elasticity, however, might suggest a small effect size, and thus might not be significant with our small study population. This result applies to the complete assigned regimen and does not establish a class-wide effect.

Report / PMID	Assigned regimen	Selected endpoint and time	Finding and interpretation
El-Domyati et al., 2018 | 29226630	Twenty-four volunteers with postacne atrophic scars were randomly divided into 3 equal groups according to performed procedure on each side of the face (microneedling by dermaroller alone or combined with platelet-rich plasma [PRP] or trichloroacetic acid [TCA] 15% peeling) and received 6 bi-weekly sessions of treatment.	Combined treatment of dermaroller and PRP or dermaroller and TCA 15% showed significant improvement when compared with dermaroller alone (P = .015 and .011 respectively).	Combined treatment of dermaroller and PRP or dermaroller and TCA 15% showed significant improvement when compared with dermaroller alone (P = .015 and .011 respectively). Epidermal thickness showed statistically significant increase in studied groups, mainly after dermaroller and TCA 15%. This result applies to the complete assigned regimen and does not establish a class-wide effect.
Min et al., 2018 | 29266290	Subjects with mild to moderate acne scars were treated with two sessions of fractional CO 2 laser therapy given with and without co-administration of PRP.	The PRP treatment increased clinical efficacy with decreased severity of adverse effects such as erythema, swelling and oozing.	The PRP treatment increased clinical efficacy with decreased severity of adverse effects such as erythema, swelling and oozing. Productions of TGFβ1 and TGFβ3 proteins were more highly elevated on the PRP-treated side of the face compared to the control side at day 28. This result applies to the complete assigned regimen and does not establish a class-wide effect.
Jones et al., 2018 | 29381544	The right or left cheek was randomized to receive 1 mL of SP-HAL using a microdroplet technique, with the contralateral cheek treated with sham injection of sterile normal saline.	Fourteen patients completed the study.	Fourteen patients completed the study. There were no statistically significant improvements in wrinkling or elastosis of the SP-HAL-treated cheek or control cheek at any time point. This result applies to the complete assigned regimen and does not establish a class-wide effect.
Cohen et al., 2019 | 29762642	152 subjects (25-65 years) were randomized (1:1) to a treatment-sequence of onabotulinumtoxinA/hyaluronic acid or hyaluronic acid/onabotulinumtoxinA, with initial treatment administered on day 1 and 6 additional visits: week 2 (touch-up); week 4 (crossover); week 6 (touch-up); and weeks 8, 12, and 24 (follow-up).	Between 92% and 100% of subjects in each treatment-sequence group exhibited at least some improvement from baseline at each study visit in the Physician Aesthetic Improvement Scale and the Objective Observer and Patient Global Assessments of Improvement,…	Between 92% and 100% of subjects in each treatment-sequence group exhibited at least some improvement from baseline at each study visit in the Physician Aesthetic Improvement Scale and the Objective Observer and Patient Global Assessments of Improvement, with no significant between-sequence differences. Subjects reported looking 3 to 6 years younger at each visit, with significant improvements in glabellar, lateral canthal, and… This result applies to the complete assigned regimen and does not establish a…
Freire Dos Santos et al., 2018 | 30076447	Fifteen women (mean age 55) willing to participate in a randomized, double-blind, face-split trial with HA and placebo formulations in different sides of the face, were subject to five treatment sessions with high-frequency high-intensity ultrasound. Photographs taken before the procedure and after the last procedure were evaluated by a panel of five…	The expert panel identified a noticeable facial rejuvenation in the HA side relative to the placebo with a very statistically significant difference between the two sides ($p < 0.0001$).	The expert panel identified a noticeable facial rejuvenation in the HA side relative to the placebo with a very statistically significant difference between the two sides ($p < 0.0001$). This result applies to the complete assigned regimen and does not establish a class-wide effect.
El-Domyati et al., 2018 | 30105816	Twenty-four volunteers with photoaging were randomly divided into three equal groups according to performed procedure on each side of the face (microneedling by dermaroller alone or combined with PRP or TCA 15% peeling). They had received one session every 2 weeks for six sessions of treatment.	Combined treatment of dermaroller and PRP or dermaroller and TCA showed significant improvement when compared with dermaroller alone.	Combined treatment of dermaroller and PRP or dermaroller and TCA showed significant improvement when compared with dermaroller alone. Significant increase in epidermal thickness was apparent in studied groups, especially after combined treatment with TCA. This result applies to the complete assigned regimen and does not establish a class-wide effect.
Prakoeswa et al., 2019 | 30265171	This was an analytic experimental research conducted on a total of 48 women who were randomized into two groups: 24 women received amniotic membrane stem cell-conditioned medium (AMSC-CM) and the other 24 women received normal saline (NS). We evaluated the progression of photoaging on Weeks 0, 4, and 8, as well as the side effects.	The improvement in photoaging after treatment showed significant better effects with the AMSC-CM than with NS (p	The improvement in photoaging after treatment showed significant better effects with the AMSC-CM than with NS (p This result applies to the complete assigned regimen and does not establish a class-wide effect.
Kang et al., 2019 | 30375189	The skin irritation test was assessed by expert interviewers to elucidate undesirable side effects.	The combinatorial therapies showed statistically significant efficacy for the improvement of average depth of wrinkles, dermal density, elasticity, and hydration after an 8-week application ($P < 0.001$).	The combinatorial therapies showed statistically significant efficacy for the improvement of average depth of wrinkles, dermal density, elasticity, and hydration after an 8-week application ($P < 0.001$). Adverse effects on the skin were not observed in any subject during the test period. This result applies to the complete assigned regimen and does not establish a class-wide effect.
Sasaki et al., 2019 | 30534955	Each of 10 patients received equal volumes of syringe-harvested, centrifuged fat to opposing midfaces with a lateral submuscular aponeurotic system-plication or no face lift and hands that were combined with equal volumes of either concentrated PRP or normal saline.	The average percent change in mean volume assessments at the fat/PRP sites from baseline values, as profiled by 3D Vectra Analysis, demonstrated a higher, but statistically nonsignificant value over 1 year than the percent value changes at the fat/normal…	The average percent change in mean volume assessments at the fat/PRP sites from baseline values, as profiled by 3D Vectra Analysis, demonstrated a higher, but statistically nonsignificant value over 1 year than the percent value changes at the fat/normal saline sites in the opposing face or hand. Three independent evaluators were able to assess volume restorations to the malar fat pad, naso-jugal groove, and nasolabial fold as well as… This result applies to the complete assigned regimen and does not establish a…
Bohnert et al., 2019 | 30741790	Forty healthy women were enrolled in this randomized, controlled, double-blind, multicenter study. Eligible subjects received 3 treatments every 4 weeks with either PLLA (treatment group) or saline (control group) injections, into both sides of the face.	At the 12-month follow-up, there was a statistically significant increase of skin elasticity and hydration in PLLA-treated subjects and a decrease in transepidermal water loss in both groups.	At the 12-month follow-up, there was a statistically significant increase of skin elasticity and hydration in PLLA-treated subjects and a decrease in transepidermal water loss in both groups. Pigmentation, erythema, and pore size were significantly decreased, whereas radiance and smoothness were significantly increased at 12 months per blinded investigator rating in this group. This result applies to the complete assigned regimen and does not establish a class-wide effect.

Report / PMID	Assigned regimen	Selected endpoint and time	Finding and interpretation
Bhargava et al., 2019 | 30924301	A total of 30 patients with grade 4 acne scars were randomly divided into two groups, 15 patients each: Group A underwent three sequential treatments of subcision and needling while Group B, three sequential treatments of subcision, needling, and topical application of PRP that were performed at 3-week intervals.	Scar improvement ≥50% was reported significantly more often by Group B than Group A patients (P = 0.025).	Scar improvement ≥50% was reported significantly more often by Group B than Group A patients (P = 0.025). Regarding physician-based assessment of scar grading post-therapy (number of patients with two grades improvement vs one grade or no improvement), there was a trend toward more improvement in Group B (P = 0.195). This result applies to the complete assigned regimen and does not establish a class-wide effect.
Galal et al., 2019 | 30964227	Patients were randomized to receive fractional CO2 laser therapy to one side of the face while the other side of the face was treated with fractional CO2 laser followed by intradermal PRP injection.	A dramatic improvement was observed in the scar depth on both sides of the face.	A dramatic improvement was observed in the scar depth on both sides of the face. However, the combined fractional CO2 laser and PRP showed more significant improvement. This result applies to the complete assigned regimen and does not establish a class-wide effect.
Park et al., 2021 | 31172821	Both cheeks of 15 subjects were treated with a fractional CO 2 laser. HSCM was randomly applied to one of the resurfacing sites (T) and normal saline was applied to the other site as a control (C).	After two months, the scar volume was reduced by 23.5% (T) versus 15.0% (C) (p = .143) and the volume of the skin pores was reduced by 37.6% (T) versus 15.9% (C) (p = .006), while the erythema was increased by 2.8% (T) versus 3.1% (C) (p = .934).	After two months, the scar volume was reduced by 23.5% (T) versus 15.0% (C) (p = .143) and the volume of the skin pores was reduced by 37.6% (T) versus 15.9% (C) (p = .006), while the erythema was increased by 2.8% (T) versus 3.1% (C) (p = .934). Atrophic scar and the skin pores in the HSCM-applied area improved by at least 15.0% after a single treatment session, suggesting better results compared with the control side. This result applies to the complete assigned regimen and does not establish a class-wide…
Abdel-Maguid et al., 2021 | 31180258	Thirty-three patients were randomly divided into two split-face groups. Group I (n = 17) received FCL plus topical SC-CM on one side or FCL plus saline on the other.	No significant difference in clinical improvement of acne scars was observed between the FCL/SC-CM and FCL only sides (p = .63), while better and faster improvement was detected on FCL/PRP side compared to FCL/SC-CM side (p = .006).	No significant difference in clinical improvement of acne scars was observed between the FCL/SC-CM and FCL only sides (p = .63), while better and faster improvement was detected on FCL/PRP side compared to FCL/SC-CM side (p = .006). There was no significant difference in downtime or adverse effects between the treated sides in either group. This result applies to the complete assigned regimen and does not establish a class-wide effect.
Hassan et al., 2020 | 31241854	Each patient received three monthly sessions. Each side of the face was randomly treated either with intradermal PRP alone or with combined treatment with subcision followed by PRP injection.	Platelet-rich plasma alone showed a better response, fewer side effects, and shorter downtime compared to combined subcision and PRP.	Platelet-rich plasma alone showed a better response, fewer side effects, and shorter downtime compared to combined subcision and PRP. This result applies to the complete assigned regimen and does not establish a class-wide effect.
Kim et al., 2020 | 31328871	Twenty-three patients who underwent AFL on both cheeks applied a human umbilical cord blood-derived mesenchymal stem cell (hUCBMSC)-conditioned media containing cream with or without stem cell containing serum application.	The area of total microcrusts was reduced in the study group which applied both serum and cream.	The area of total microcrusts was reduced in the study group which applied both serum and cream. The global improvement score of the post-treatment erythema was significantly reduced. This result applies to the complete assigned regimen and does not establish a class-wide effect.
Hedén et al., 2019 | 31334927	Effective and Safe Repeated Full-Face Treatments With AbobotulinumtoxinA, Hyaluronic Acid Filler, and Skin Boosting Hyaluronic Acid.	Repeated full-face treatment with ABO, HA filler, and RSB was associated with better aesthetic outcome and higher levels of satisfaction than treatment with ABO or HA filler alone.	Repeated full-face treatment with ABO, HA filler, and RSB was associated with better aesthetic outcome and higher levels of satisfaction than treatment with ABO or HA filler alone. However, even modest volumes of HA filler achieved good aesthetic outcomes and high satisfaction. This result applies to the complete assigned regimen and does not establish a class-wide effect.
Jeong et al., 2020 | 31680395	A total of 30 subjects with symmetric crow's feet of 2-4 points on the Crow's Feet Grading Scale (CFGS) were enrolled in this randomized, patient/evaluator-blinded, split-face study. Each subject was randomized to receive injections of DLMR01 or RJR in their right or left crow's feet.	No significant difference in CFGS, GAIS, and R a value was detected between DLMR01 side and RJR at 12 weeks (improvement rate in CFGS from baseline at week 12-DLMR01: 48.28% [14/29], RJR: 41.38% [12/29]).	No significant difference in CFGS, GAIS, and R a value was detected between DLMR01 side and RJR at 12 weeks (improvement rate in CFGS from baseline at week 12-DLMR01: 48.28% [14/29], RJR: 41.38% [12/29]). This result applies to the complete assigned regimen and does not establish a class-wide effect.
Elfar et al., 2020 | 32108322	Sixty patients with atrophic postacne scars were enrolled in this single blinded randomized controlled study. The patients were divided into three groups with 20 patients being treated with intradermal injection of plasma gel, 20 patients treated with dermaroller, and 20 patients subjected to combined plasma gel and dermaroller.	There was statistically significant improvement in postacne scars after treatment in all studied groups with variable degrees; the combined technique showed the best clinical improvement in postacne scars.	There was statistically significant improvement in postacne scars after treatment in all studied groups with variable degrees; the combined technique showed the best clinical improvement in postacne scars. There was an increase in newly formed collagen and elastic fibers with more organized and condensed bundles after the end of treatment. This result applies to the complete assigned regimen and does not establish a class-wide effect.
Lee et al., 2022 | 32248707	A total of 27 subjects were enrolled in this randomized, pair-matched, and active-controlled study. Each subject received filler injections thrice with two-week intervals, with a PN filler injection on one side and a non-crosslinked hyaluronic acid (HA) filler injection on the contralateral side of the periorbital area.	Improvements in the visual analog scale and global esthetic improvement scale scores were not significantly different between the PN and HA groups.	Improvements in the visual analog scale and global esthetic improvement scale scores were not significantly different between the PN and HA groups. The improvement rates of skin elasticity and hydration decreased over time in both groups, with the PN group showing a higher improvement rate. This result applies to the complete assigned regimen and does not establish a class-wide effect.
Mahamoud et al., 2020 | 32333512	All the patients received 3 treatments of fractional CO 2 laser combined with intradermal injection of PRP at one side of the face compared with intradermal injection of noncross-linked hyaluronic acid at the other side of the face.	The study included 30 patients ages ranging from 25 to 44 years old.	The study included 30 patients ages ranging from 25 to 44 years old. There was a statistically significant reduction in the severity of acne scars on both sides after treatment using qualitative and quantitative Goodman and Baron. This result applies to the complete assigned regimen and does not establish a class-wide effect.

Report / PMID	Assigned regimen	Selected endpoint and time	Finding and interpretation
Antonino et al., 2021 | 32385943	Women with moderate-to-severe atrophic scars of the face were enrolled on the study.	From October to December 2019, twenty women which fitted the inclusion criteria signed a consent form and received 3.0 mL of calcium hydroxylapatite and after 4 weeks, 400 lines of HIFU.	From October to December 2019, twenty women which fitted the inclusion criteria signed a consent form and received 3.0 mL of calcium hydroxylapatite and after 4 weeks, 400 lines of HIFU. No major side effects were reported during the study, and all patients completed the follow-up after 6 months. This result applies to the complete assigned regimen and does not establish a class-wide effect.
Jang et al., 2020 | 32421218	Clinical efficacy and safety tests were performed for 12 weeks on twenty three female subjects with wrinkles around their eyes.	Both Ad-HMN and Ad-LMN groups showed statistically significant efficacy for almost all parameters.	Both Ad-HMN and Ad-LMN groups showed statistically significant efficacy for almost all parameters. The Ad-HMN patch had better effect on the mean depth of biggest wrinkles, maximum depth of biggest wrinkles, dermal density, and skin elasticity than the Ad-LMN patch. This result applies to the complete assigned regimen and does not establish a class-wide effect.
Zvezdin et al., 2020 | 32485024	The effectiveness of microneedle applicators was confirmed by a randomized split-phase study involving 82 subjects.	The results of the profilometric evaluation of skin smoothness demonstrated a significant reduction in the average roughness index by 65.32 ± 2.99% and in the mean skin waviness by 66.84 ± 1.6% compared with these indicators in the control group ($P < 0.05$).	The results of the profilometric evaluation of skin smoothness demonstrated a significant reduction in the average roughness index by 65.32 ± 2.99% and in the mean skin waviness by 66.84 ± 1.6% compared with these indicators in the control group ($P < 0.05$). The therapeutic effect of the procedure was confirmed by an ultrasound examination that registered a 72.2 ± 5.4% and 25 ± 1.4% increase in echo-density of the epidermis and dermis,… This result applies to the complete assigned regimen and does not establish a…
Polacco et al., 2021 | 32506124	Patients were randomized to receive either CPM-HA in the left perioral region and SPHAL in the right or vice versa.	Both SP-HAL and CPM-HA achieved a reduction in rhytid severity, and neither treatment group returned to baseline after 180 days.	Both SP-HAL and CPM-HA achieved a reduction in rhytid severity, and neither treatment group returned to baseline after 180 days. Investigator-reported scores for rhytid severity were significantly better for SP-HAL than CPM-HA and remained so at 180 days ($P < .05$). This result applies to the complete assigned regimen and does not establish a class-wide effect.
Choi et al., 2020 | 32621657	Effects of hyaluronic acid injected using the mesogun injector with stamp-type microneedle on skin hydration.	Selected clinical or instrumental skin outcome at the final common assessment reported in the accessible source.	The accessible report identified a randomized comparison but did not provide sufficiently detailed result text in the indexed record; interpretation is therefore conservative. This result applies to the complete assigned regimen and does not establish a class-wide effect.
El-Hawary et al., 2021 | 32770685	Sixty patients were randomly divided into three equal groups. Group 1 received intradermal PRP injection sessions.	Combined PRP and FCO 2 laser-treated patients had a better clinical response, fewer side effects, and shorter downtime than FCO 2 laser alone.	Combined PRP and FCO 2 laser-treated patients had a better clinical response, fewer side effects, and shorter downtime than FCO 2 laser alone. PRP-treated patients had some improvement but significantly lower than the other two groups. This result applies to the complete assigned regimen and does not establish a class-wide effect.
Hu et al., 2021 | 32816021	This was a 12-week randomized, placebo-controlled trial conducted at a single center comparing 2 groups. Thirty patients received a PRFM injection in the mid-cheek region and nasolabial fold on one side of the face and saline on the contralateral side.	There were 30 participants, with a mean [standard deviation] age of 49.9 [13.9] years.	There were 30 participants, with a mean [standard deviation] age of 49.9 [13.9] years. At 6 weeks, the median change in total VISIA score (interquartile range) was -1.77 (2.36) in the PRFM group and -0.73 (2.09) in the saline group ($P = 0.003$). This result applies to the complete assigned regimen and does not establish a class-wide effect.
Kollipara et al., 2020 | 32852426	The patients were randomized to receive 3 treatments of PLLA in 1 knee, whereas the other knee received 3 treatments of bacteriostatic water.	Statistically significant improvement as rated on the physician global aesthetic improvement scale was seen at Day 56 after final treatment in the active knee when compared with the placebo knee.	Statistically significant improvement as rated on the physician global aesthetic improvement scale was seen at Day 56 after final treatment in the active knee when compared with the placebo knee. This improvement was sustained at Day 84 and Day 168 after final treatment visits. This result applies to the complete assigned regimen and does not establish a class-wide effect.
Hoss et al., 2020 | 33196737	Red Deer Umbilical Cord-Derived Stem Cell Conditioned Media Combined With Ablative Resurfacing of the Face.	Both the active and vehicle pre-procedure cream were soothing, calming and easy to use by all patients.	Both the active and vehicle pre-procedure cream were soothing, calming and easy to use by all patients. There was a trend towards decreased facial erythema and crusting in the active versus vehicle group. This result applies to the complete assigned regimen and does not establish a class-wide effect.
Bartsch et al., 2020 | 33196744	61 females (body mass index: 22.6 ± 2.4 kg/m², age: 37.2 ± 6.8 years) were enrolled in this randomized interventional prospective study.	No adverse events were observed during the study that required intervention outside the standard of care treatment protocol.	No adverse events were observed during the study that required intervention outside the standard of care treatment protocol. The combination of three treatment modalities was shown to provide greater improvement in skin laxity 1.88 (95% CI, 0.66–5.37) and skin dimpling 1.31 (95% CI, 0.61–2.81) scores as compared to any combination of two modalities. This result applies to the complete assigned regimen and does not establish a class-wide effect.
Hersant et al., 2021 | 33534905	For this randomized controlled prospective study, 93 eligible patients were enrolled and randomized into 3 intervention groups to undergo a series of 3 treatment sessions with either a-PRP, HA, or a mixture of a-PRP and HA (Cellular Matrix; Regen Lab) injected into facial cheeks.	A total of 93 patients were included.	A total of 93 patients were included. Treatment with Cellular Matrix led to a very significant improvement in the overall facial appearance compared with treatment with a-PRP or HA alone ($P < 0.0001$). This result applies to the complete assigned regimen and does not establish a class-wide effect.
Gawdat et al., 2022 | 34214220	20 patients with mild to moderate neck laxity were randomized to receive 3 sessions of either fractional radiofrequency	Both Groups showed a statistically significant improvement in all parameters.	Both Groups showed a statistically significant improvement in all parameters. Comparing the two groups, the mean dermal thickness after treatment was higher in group A compared with B but was found

Report / PMID	Assigned regimen	Selected endpoint and time	Finding and interpretation
	microneedling +PRP (group A) or fractional radiofrequency microneedling monotherapy (group B).		statistically insignificant. This result applies to the complete assigned regimen and does not establish a class-wide effect.
Vachiramon et al., 2021 | 34352146	We conducted a randomized, single-blinded, split-face study on participants with enlarged facial pores. Each side of the face was randomly assigned to treatment with one session of single technique or combined technique.	Forty-six participants completed the study.	Forty-six participants completed the study. The mean pore volume of both sides declined with statistical significance at every visit compared to baseline, with the lowest mean at 4 months post-treatment. This result applies to the complete assigned regimen and does not establish a class-wide effect.
Ebrahim et al., 2022 | 34927342	After the last session of subcision, the patients were reclassified randomly into 2 equal groups each received either HA or threads in one side of the face leaving the other side that underwent subcision before as a control.	A significant clinical improvement was achieved in 67.3% of patients after subcision alone compared to 94.1%, 82.4% of subcision combined with HA or threads, respectively.	A significant clinical improvement was achieved in 67.3% of patients after subcision alone compared to 94.1%, 82.4% of subcision combined with HA or threads, respectively. Patients' satisfaction was higher in the combined groups compared to subcision alone ($p < 0.0001$). This result applies to the complete assigned regimen and does not establish a class-wide effect.
Siperstein et al., 2022 | 35333199	Twenty-six subjects were randomized to receive up to 1 mL of the HA filler Restylane Refyne (HARR, n = 20) or saline (n = 6) using a cannula on one side and a needle on the other with optional repeat treatment.	A significant improvement 30 days after the last treatment was achieved on the Transverse Neck Line Scale when comparing HARR with saline according to the blind evaluator, subjects, and investigator without any significant side effects.	A significant improvement 30 days after the last treatment was achieved on the Transverse Neck Line Scale when comparing HARR with saline according to the blind evaluator, subjects, and investigator without any significant side effects. In addition, the blind evaluator, subjects, and investigator rated the side of the neck injected with a needle as having significantly greater improvement than the cannula. This result applies to the complete assigned regimen and does not establish a class-wide effect.
Gawdat et al., 2022 | 35435087	Treatment with fractional CO 2 laser plus plasma fluid/gel was randomly assigned to the right/left sides of the face.	There was a significant improvement in clinical assessment scores at third-month follow-up on the plasma gel- and plasma fluid-treated sides compared to those at the first-month follow-up (p 001).	There was a significant improvement in clinical assessment scores at third-month follow-up on the plasma gel- and plasma fluid-treated sides compared to those at the first-month follow-up (p 001). Scar depth decreased significantly at third-month follow-up when compared to baseline on both plasma gel- and plasma fluid-treated sides (p 001). This result applies to the complete assigned regimen and does not establish a class-wide effect.
Noormohammadpour et al., 2022 | 35775872	Botulinum toxin injection as a single or combined treatment with non-cross-linked high molecular weight and low molecular weight hyaluronic acid gel for neck rejuvenation: A randomized clinical trial.	Selected clinical or instrumental skin outcome at the final common assessment reported in the accessible source.	The accessible report identified a randomized comparison but did not provide sufficiently detailed result text in the indexed record; interpretation is therefore conservative. This result applies to the complete assigned regimen and does not establish a class-wide effect.
Ismail et al., 2022 | 36219518	Which is more effective in atrophic acne scars treatment microneedling alone or platelet rich plasma alone or combined both therapeutic modalities?	Selected clinical or instrumental skin outcome at the final common assessment reported in the accessible source.	The accessible report identified a randomized comparison but did not provide sufficiently detailed result text in the indexed record; interpretation is therefore conservative. This result applies to the complete assigned regimen and does not establish a class-wide effect.
Seoudy et al., 2023 | 36374507	Twenty-six female patients were selected and each face was split into 2 halves a control side (Laser + saline) and an experimental side (Laser + platelet rich plasma).	The combined treatment was as effective in improving wrinkles as fractional CO 2 laser alone.	The combined treatment was as effective in improving wrinkles as fractional CO 2 laser alone. Antera camera scores showed statistical significance in overall wrinkles measurements on each side equally. This result applies to the complete assigned regimen and does not establish a class-wide effect.
Mehrabi et al., 2023 | 37052609	Thirty patients with moderate-to-severe atrophic acne scarring were included in this prospective, split-face, double-blinded, randomized controlled study. Each underwent 3 monthly injections of a novel formula of combined high and low molecular weight HA (P) to the base of acne scars on 1 side of the face and traditional cross-linking HA (JV) filler on the…	For JV, statistically significant reductions were observed in the acne scar volume but nearly no change in elasticity and stretch during early treatments.	For JV, statistically significant reductions were observed in the acne scar volume but nearly no change in elasticity and stretch during early treatments. For P, no significant differences were observed in early treatments; however, statistically significant improvements were observed in later visits. This result applies to the complete assigned regimen and does not establish a class-wide effect.
Kaminaka et al., 2023 | 37208833	Clinical evaluation of needle-free electronic pneumatic hyaluronic acid injection treatment for facial atrophic acne scars: A prospective, randomized comparative trial.	Selected clinical or instrumental skin outcome at the final common assessment reported in the accessible source.	The accessible report identified a randomized comparison but did not provide sufficiently detailed result text in the indexed record; interpretation is therefore conservative. This result applies to the complete assigned regimen and does not establish a class-wide effect.
Han et al., 2023 | 37632290	A 24-week prospective, single-center, assessor-blinded, randomized, split-face study was conducted.	A single treatment of filler injection with a laser-induced microjet injector resulted in similar improvements in skin hydration and elasticity as a single treatment of filler injection by using manual needle injection, with reduced pain, side effects, and…	A single treatment of filler injection with a laser-induced microjet injector resulted in similar improvements in skin hydration and elasticity as a single treatment of filler injection by using manual needle injection, with reduced pain, side effects, and decreased treatment time. This result applies to the complete assigned regimen and does not establish a class-wide effect.
Pincelli et al., 2024 | 37903326	In this prospective, randomized clinical trial, 18 women with hand aging received PRP injections every 4 weeks into the unilateral dorsal hand for 12 weeks total; with saline injections into the contralateral hand in a randomized, controlled, single-blind fashion.	The majority of patients reported pain and discomfort along with a burning/stinging sensation in both PRP- and saline-treated hands, with no significant differences noted in any	The majority of patients reported pain and discomfort along with a burning/stinging sensation in both PRP- and saline-treated hands, with no significant differences noted in any patient outcome measures between the 2 treatments (all $P \geq 0.25$). No differences were reliably detected between the treatment hands by a blinded investigator comparing before-and-after clinical photographs of the hands. This result applies to the complete assigned regimen and does not establish a class-wide effect.

Report / PMID	Assigned regimen	Selected endpoint and time	Finding and interpretation
		patient outcome measures between the 2 treatments (all P ≥ 0.25).	
Wu et al., 2024 | 38051121	This prospective, randomized study included C57BL/6 mice treated with MFR, MFR plus PLLA, and carbon dioxide laser plus PLLA and 32 patients who underwent split-face treatments with MFR or MFR plus PLLA twice every 2 months. The Global Aesthetic Improvement Scale, Facial Laxity Rating scale of the whole face, the Echelle d'Evaluation Clinique des…	PLLA was delivered by channels produced by MFR but not carbon dioxide laser in the mouse model.	PLLA was delivered by channels produced by MFR but not carbon dioxide laser in the mouse model. Thirty patients were treated with split-face MFR plus PLLA or MFR, revealing an improvement in VISIA wrinkle percentile (0.020) compared with the age-matched controls (0.000). This result applies to the complete assigned regimen and does not establish a class-wide effect.
Fabi et al., 2024 | 38206151	Male/female immune-competent adults (aged >21 years) with moderate/severe cheek wrinkles, graded using the Galderma Cheek Wrinkle Scale (GCWS) at rest, were randomized 2:1 to receive PLLA-SCA injections (150 mg; 8 mL reconstitution in sterile water for injection) + 1 mL lidocaine hydrochloride (2%), administered immediately after reconstitution, or no…	GCWS at rest responder rate was significantly higher with PLLA-SCA treatment versus the no-treatment control at months 7 (66.2% versus 38.6%; P=0.0043), 9 (70.6% versus 31.1%; P 95%), tighter appearance (>88%), and jawline contour (>85%).	GCWS at rest responder rate was significantly higher with PLLA-SCA treatment versus the no-treatment control at months 7 (66.2% versus 38.6%; P=0.0043), 9 (70.6% versus 31.1%; P 95%), tighter appearance (>88%), and jawline contour (>85%). PLLA-SCA recipients reported high satisfaction levels regarding improvements in skin radiance (90% or greater), sagging (84% or greater), and firmness (91% or greater) as well as natural-looking… This result applies to the complete assigned regimen and does not establish a…
Tsai et al., 2024 | 38253883	This single-center, double-blind, randomized controlled trial was conducted from January 1, 2022, to July 31, 2022, and included ten participants who completed the follow-up. The participants were randomly assigned to receive 2.5-mL injections of PRP and PPP on different sides of the face in three sessions with 1-month intervals.	Both PRP and PPP treatments resulted in significant improvement in the Global Aesthetic Improvement Scales and Modified Fitzpatrick Wrinkle Scale for periocular Powered by Editorial Manager® and ProduXion Manager® from Aries Systems Corporation wrinkles,…	Both PRP and PPP treatments resulted in significant improvement in the Global Aesthetic Improvement Scales and Modified Fitzpatrick Wrinkle Scale for periocular Powered by Editorial Manager® and ProduXion Manager® from Aries Systems Corporation wrinkles, with no significant difference between the two groups. However, no improvement was observed in the Wrinkle Severity Rating Scales for nasolabial folds in either the PRP- or… This result applies to the complete assigned regimen and does not establish a class-wide…
Dastgheib et al., 2024 | 38429946	Twelve patients aged 18-45 years with atrophic acne scars on both sides of the face participated in this single-blinded, split-face, randomized controlled trial. Each side of the face was randomly assigned to one of the treatment methods, including subcision alone and subcision + Profhilo.	The VAS score of patient satisfaction was statistically significant in the Profhilo arm, with a mean improvement of 528.08 and 219.06 in the subcision arm (p = 0.02).	The VAS score of patient satisfaction was statistically significant in the Profhilo arm, with a mean improvement of 528.08 and 219.06 in the subcision arm (p = 0.02). No significant difference was seen in total acne scar reduction comparing the two methods (29.74 in the Profhilo arm and 22.27 in the subcision arm, p = 0.56). This result applies to the complete assigned regimen and does not establish a class-wide effect.
Faria et al., 2024 | 38831064	The control arm (CA) received conventional subdermal treatment with CaHA at a 1:1 dilution.	Both the CA and the IA exhibited high rates of patient satisfaction and satisfaction as assessed by blinded evaluators.	Both the CA and the IA exhibited high rates of patient satisfaction and satisfaction as assessed by blinded evaluators. Although numerical superiority was observed in the IA, no statistical difference was found between the two groups. This result applies to the complete assigned regimen and does not establish a class-wide effect.
Tejero García et al., 2024 | 38836757	We conducted a prospective, single-center, randomized pilot study on 10 healthy female volunteers from 30 to 65 years with hand aging signs. Patients received two sessions of MCT Plasma on the treated hand and two sessions of standard platelet-rich plasma (PRP) on the control hand.	Ten women with a mean age of 57.5 years (standard deviation 10.5, range 31 - 67) were included, and seven (70%) completed the study.	Ten women with a mean age of 57.5 years (standard deviation 10.5, range 31 - 67) were included, and seven (70%) completed the study. The treated hands' skin subepidermal low-echogenic band (SLEB) decreased from 20% to 60%, and 57.1% (n = 4) had better results than control. This result applies to the complete assigned regimen and does not establish a class-wide effect.
Camargo et al., 2024 | 39504084	Clinical evaluation of combined autologous Platelet-Rich Plasma and Volume-Controlled Ozone Therapy in Facial Rejuvenation: A randomized controlled Pilot Study.	Selected clinical or instrumental skin outcome at the final common assessment reported in the accessible source.	The accessible report identified a randomized comparison but did not provide sufficiently detailed result text in the indexed record; interpretation is therefore conservative. This result applies to the complete assigned regimen and does not establish a class-wide effect.
Doyle et al., 2025 | 39511699	Twelve healthy women aged 35 to 65 years were randomized into 2 groups. Group A received MFU-V followed by hyperdilute CaHA-CMC 6 weeks later, while Group B received the treatments in the opposite order.	Group A showed greater improvements in subjective Global Aesthetic Improvement Scale, investigator Global Aesthetic Improvement Scale, and FACE-Q Patient-Perceived Age visual analog scale scores.	Group A showed greater improvements in subjective Global Aesthetic Improvement Scale, investigator Global Aesthetic Improvement Scale, and FACE-Q Patient-Perceived Age visual analog scale scores. Both groups exhibited similar improvements in FACE-Q Satisfaction with Lower Face and Jawline scores. This result applies to the complete assigned regimen and does not establish a class-wide effect.
Rageh et al., 2025 | 40167803	Fractional CO2 laser with topical insulin versus PRP for atrophic acne scars: a randomized split-face study.	Selected clinical or instrumental skin outcome at the final common assessment reported in the accessible source.	The accessible report identified a randomized comparison but did not provide sufficiently detailed result text in the indexed record; interpretation is therefore conservative. This result applies to the complete assigned regimen and does not establish a class-wide effect.
Qi et al., 2025 | 40254943	Eighty patients with periorbital wrinkles and eyelid laxity were randomly assigned to a laser group (n = 40) receiving LEUCO 2 FL or a combination group (n = 40) receiving additional autologous PRP. Both groups received treatment once per month for a total of three sessions.	The combination group exhibited higher overall effective rate of GAIS, greater improvements in wrinkles, texture, skin elasticity, and moisture at all time points, and lower upper eyelid depression and infraorbital hollowing scores than the laser group.	The combination group exhibited higher overall effective rate of GAIS, greater improvements in wrinkles, texture, skin elasticity, and moisture at all time points, and lower upper eyelid depression and infraorbital hollowing scores than the laser group. While both treatments were well tolerated, patient satisfaction was higher in the combined group than the laser group. This result applies to the complete assigned regimen and does not establish a class-wide effect.

Report / PMID	Assigned regimen	Selected endpoint and time	Finding and interpretation
Park et al., 2025 | 40826297	A prospective, open-label, randomized study involved 17 participants with Grade 2-5 acne scars. They were divided into 3 groups: Group 1 received air-botulinum toxin, Group 2 received PDRN, and Group 3 received both.	Group 3, which received the combination treatment, showed the greatest improvement, with a 12.0-point reduction in POSAS scores.	Group 3, which received the combination treatment, showed the greatest improvement, with a 12.0-point reduction in POSAS scores. Group 1 saw a 9.0-point reduction, and Group 2 had a 6.1-point reduction. This result applies to the complete assigned regimen and does not establish a class-wide effect.
Lee et al., 2026 | 40899732	A 4-week prospective, single-center, evaluator-blinded, randomized, split-face study was conducted.	Microsubcision with HA improved scarring depth and postacne erythema more than CO 2 pinhole method, although the differences were not statistically significant.	Microsubcision with HA improved scarring depth and postacne erythema more than CO 2 pinhole method, although the differences were not statistically significant. Histopathologic analysis revealed that microsubcision with HA induced diffuse soft tissue remodeling and evenly distributed fine collagen fibers. This result applies to the complete assigned regimen and does not establish a class-wide effect.
Alzayadneh et al., 2026 | 41129279	In this prospective, randomized, double-blind, split-face trial, 52 participants (73.1% female; mean age 44.5 ± 4.7 years) received BoNT-A + NCTF 135HA on one facial side and BoNT-A alone contralaterally.	Mean WSRS reduction was greater with the combination (Δ = 2.33 ± 0.70) vs BoNT-A alone (Δ = 1.34 ± 0.59; d = 1.35, P < .001).	Mean WSRS reduction was greater with the combination (Δ = 2.33 ± 0.70) vs BoNT-A alone (Δ = 1.34 ± 0.59; d = 1.35, P < .001). Hydration increased significantly only on the combination side (Δ = 3.71 ± 1.48; d = 2.35, P < .001), with larger gains also in firmness (d = 2.11), tone homogeneity (d = 1.51), and radiance (d = 1.16; all P < .001). This result applies to the complete assigned regimen and does not establish a class-wide effect.
Roohaninasab et al., 2026 | 41168367	Between 2023 and 2024, a double-blind, randomized clinical trial was conducted involving 15 patients aged 18 to 65 with atrophic acne scars at a dermatology clinic. Participants were divided into three groups: one group received a combination of platelet-rich plasma (PRP) and non-cross-linked hyaluronic acid on one side of the face, while the opposite side…	Results indicated that while no significant differences were found in colorimetry, tewametry, and corneometry across groups, the PRP group had higher erythema and melanin indices in the third evaluation (three months after the first treatment session)…	Results indicated that while no significant differences were found in colorimetry, tewametry, and corneometry across groups, the PRP group had higher erythema and melanin indices in the third evaluation (three months after the first treatment session) compared to the PRP+non-cross-linked hyaluronic acid (HA) group. Conversely, the non-cross-linked HA group demonstrated superior scores in tewametry and melanin index compared to the… This result applies to the complete assigned regimen and does not establish a…
Liao et al., 2026 | 41650338	In this multicenter, randomized, controlled trial, 420 subjects were enrolled (2:1, treatment: control). The treatment group received three full-face intradermal injections of the HA gel at 3-week intervals, whereas the control group received no intervention.	The treatment group showed significantly higher Global Aesthetic Improvement Scale improvement (90.63% vs 3.65%) and composite improvement rates (83.20% vs 3.65%) than control group (p < .0001), with effects sustained for 6 months.	The treatment group showed significantly higher Global Aesthetic Improvement Scale improvement (90.63% vs 3.65%) and composite improvement rates (83.20% vs 3.65%) than control group (p < .0001), with effects sustained for 6 months. Skin hydration and elasticity significantly increased from baseline (p < .05). This result applies to the complete assigned regimen and does not establish a class-wide effect.
Zanella et al., 2026 | 41731228	After selection, a blinded operator randomly allocated the right and left sides to receive either treatment (experimental group-Profhilo®) or placebo (control group-saline solution). For the first session, 3 points were marked on the cutaneous portion of the upper lip and 2 points on the lower lip, at 5 mm from the beginning of the mucosa, for each side of…	Stereophotogrammetry revealed visual improvements in wrinkles and pores after the application of Profhilo®.	Stereophotogrammetry revealed visual improvements in wrinkles and pores after the application of Profhilo®. Ultrasonography indicated an increase in dermal thickness for both Profhilo® (p = 0.016) and placebo (p < 0.001). This result applies to the complete assigned regimen and does not establish a class-wide effect.
Abdalla et al., 2026 | 42151612	This single-center, pilot, randomized, assessor-blinded controlled trial included 60 patients with severe atrophic acne scars. Patients were randomized into four groups: subcision plus SVF (Group 1), subcision plus combined SVF and PRP (Group 2), subcision plus PRP (Group 3), and subcision alone (Group 4, control).	Group 2 (subcision + SVF + PRP) demonstrated superior clinical efficacy, with 53.3% of patients achieving "Grade 3" (marked) physician satisfaction compared to 0% in the control group (p ≤ 0.001).	Group 2 (subcision + SVF + PRP) demonstrated superior clinical efficacy, with 53.3% of patients achieving "Grade 3" (marked) physician satisfaction compared to 0% in the control group (p ≤ 0.001). This group also showed significantly greater improvement in scar size and color (p ≤ 0.001). This result applies to the complete assigned regimen and does not establish a class-wide effect.
Han et al., 2026 | 42300963	Wearable near-infrared LED photothermal microneedle patch reduces periorbital wrinkles in a randomized pilot clinical trial.	Selected clinical or instrumental skin outcome at the final common assessment reported in the accessible source.	The accessible report identified a randomized comparison but did not provide sufficiently detailed result text in the indexed record; interpretation is therefore conservative. This result applies to the complete assigned regimen and does not establish a class-wide effect.
Fabi et al., 2026 | 42424535	Eligible females were randomized to immediate or delayed treatment. Patients assigned to immediate treatment received up to three diluted Radiesse sessions, with optional retreatment.	The Week 24 estimated responder rate (≥1-point improvement on the MAS Décolleté Wrinkles - At Rest) was 71.2% [95% CI: 61.4%, 79.4%] among treated patients compared with 6.3% [95% CI: 1.5%, 22.9%] among untreated controls, demonstrating a statistically…	The Week 24 estimated responder rate (≥1-point improvement on the MAS Décolleté Wrinkles - At Rest) was 71.2% [95% CI: 61.4%, 79.4%] among treated patients compared with 6.3% [95% CI: 1.5%, 22.9%] among untreated controls, demonstrating a statistically significant between-group difference. Treatment was well tolerated, with predominantly transient, mild-to-moderate injection-site reactions. This result applies to the complete assigned regimen and does not establish a class-wide effect.
Yen et al., 2026 | 42635327	Sixty adults underwent paired facial treatment from January 16, 2023, to March 15, 2026; 1 side received RFMN followed by topical EV preparation, and the contralateral side received RFMN alone. The primary endpoint was the paired between-side difference in change in wrinkle severity score at week 12.	The mean age was 43.6 (7.9) years, and 50 participants (83.3%) were women.	The mean age was 43.6 (7.9) years, and 50 participants (83.3%) were women. At week 12, wrinkle improvement was 12.59 (2.58) points on the RFMN+EV side and 9.28 (2.35) points on the RFMN side, giving a paired mean difference of 3.32 points (95% CI: 2.55-4.08). This result applies to the complete assigned regimen and does not establish a class-wide effect.

Report / PMID	Assigned regimen	Selected endpoint and time	Finding and interpretation
Qian et al., 2026 | 42869969	This was a prospective, multicenter, randomized, blank-controlled, superiority clinical trial involving 561 subjects, who were randomly assigned to either the treatment group or the control group.	The GAIS improvement rate was markedly higher in the treatment group compared with the control group (90.00% vs.	The GAIS improvement rate was markedly higher in the treatment group compared with the control group (90.00% vs. 11.90%, P < 0.0001), and this superiority was consistently observed across different analysis populations. This result applies to the complete assigned regimen and does not establish a class-wide effect.
Raspaldo et al., 2015 | 26659938	This study randomized 280 subjects desiring lip enhancement to Juvéderm Volbella with Lidocaine or Restylane-L.	Juvéderm Volbella with Lidocaine was noninferior to Restylane-L at 3 months.	Juvéderm Volbella with Lidocaine was noninferior to Restylane-L at 3 months. Investigator assessments showed significant improvements in lip fullness (P ≤ .03), perioral lines (P ≤ .04), and oral commissures (P ≤ .03) with Juvéderm Volbella with Lidocaine versus Restylane-L at months 6 to 12. This result applies to the complete assigned regimen and does not establish a class-wide effect.
Benzaquen et al., 2021 | 33792961	We conducted a prospective, double-blinded, randomized split-face study on 20 patients from 30 to 70 years old, with a skin phototype from I to IV. Each patient received full-face treatment with AFXL, immediately followed by the application in droplets into the fractional ablative tunnels of 1 ml of HA gel on one hemiface and 1 ml of physiological saline…	We showed equivalence in postlaser aftermath between HA-treated and nontreated hemiface.	We showed equivalence in postlaser aftermath between HA-treated and nontreated hemiface. Trend data analyses at the 3rd month suggested that immediate application of HA after AFXL resulted in a greater improvement in facial skin remodeling, especially for skin texture (mean score of 2.60 vs. This result applies to the complete assigned regimen and does not establish a class-wide effect.

Electronic file S3B provides full report titles, DOI or PMID identifiers, source links, outcome-denominator notes, interpretation limits, and source locators for all 159 specified results. S3C provides the corresponding 159-report bibliography and identifies whether a report is cited in the unchanged 329-item main reference list or is supplied as supplementary-only evidence.

Status	PMIDs	Interpretation
Contextual (8)	31592825; 37738217; 38968085; 39453400; 41715896; 41752855; 42403177; 42808113	Sequential/extension, adjacent indication/route or fixed-side observations; not added to the comparative count.
Noncomparative (3)	35070607; 38954627; 36909867	Randomized schedules were pooled or only within-population changes were analyzed. PMID 36909867 did not supply a verified randomized between-knee effect and its uncertainty.
**Unresolved (12)**	32655245; 41361144; 41800727; 42872269; 36606396; 20076916; 33437408; 26644738; 18833043; 21086592; 22256620; 32129049	Eight previously retrieved reports retain design/adult-eligibility uncertainty. Four additional reports remain access-unresolved after the SNU-linked recheck; no RoB 2 score was assigned to those four.

Confirmed links: 37074002/37163665 (NCT03728309); 35689521/38968085 (NCT04519515); 38206151/39453400 (NCT04124692). The 37738217/35333199 neck-report link remains qualified. Reports 40814769 and 42420660 use different toxins/ethics identifiers and remain separate. Other cohort overlaps are unresolved.

ACSS/ASRS/ATNLS = Allergan Cheek Smoothness / Skin Roughness / Transverse Neck Lines Scale; GDS = Galderma Décolletage Scale; GB/GSGS/QGSGS = Goodman–Baron/global scar grading systems; ECCA = Echelle d'évaluation clinique des cicatrices d'acné; EMG = electromyography; TEWL = transepidermal water loss. Clinical appearance, patient reports, devices, histology and safety remain distinct outcomes.
