# Supplementary material for: Biomodulators and Skin Boosters in Skin Rejuvenation: Mechanisms, Applications, and Future Perspectives
Source: Int J Mol Sci. 2026 Oct 28;27(21):9647. doi: 10.3390/ijms27219647 (PMC13762510; doi:10.3390/ijms27219647)
Supplement: Supplementary file 1 [file ijms-27-09647-s001.zip › Supplementary_Table_S4_Final.pdf]

Supplementary Table S4 Source and full text exclusions

This log documents all 209 source or full-text exclusions among the 387 reports assessed or source-adjudicated in the final flow. Grouped rows retain every excluded PMID. Electronic file S4B provides the report identity, final decision, primary exclusion reason, evidence basis, source URL, and source locator. The log belongs to the independently dated 16 October 2026 update and is not presented as a reconstruction of the unavailable historical screening process.

The remaining dispositions were 159 comparative reports, eight contextual reports, three noncomparative primary estimates, eight retrieved-unresolved reports, and four access-unresolved reports. Among the 195 candidates previously recorded as unavailable, 93 entered this source or full-text exclusion log, 94 entered the comparative set, four were excluded on explicit title or abstract evidence, and four remained access-unresolved. The 139 title and abstract exclusions among the 530 reports ever sought are not included in this source or full-text exclusion log.

Recorded principal reason	n	Excluded report PMIDs
F1 Correspondence/expert discussion	3	41725041; 42152513; 42499804
F1 Technique description	2	26644734; 39632500
F1 Technique proposal	1	41532951
F2 Adjacent pigmentary indication	1	39319696
F2 Adolescent-eligible cohort without separable adult results	1	32695692
F2 Isolated postlaser recovery	1	40590113
F2 Mixed adolescent/adult cohort without separable adult results	1	35126939
F2 Mixed scar etiologies without separable acne-scar outcomes	1	38800810
F3 Nonhuman experimental delivery	1	41295645
F4 Oral delivery	2	38495912; 39590340
F4 Ordinary topical delivery	5	38591218; 38668218; 40227405; 40536111; 40952030
F4 Ordinary topical exposure	4	25399620; 31413615; 33038010; 39009698
F4 Ordinary topical/oral delivery	2	41892958; 42698538
F5 Explicit nonrandomized design	1	34466465
F5 Explicit nonrandomized fixed-side design	1	40414798
F5 Fixed-side clinical exposure	3	25143672; 25722599; 29403184
F5 Fixed-side treatment protocol	8	32256789; 34084005; 34084007; 34084010; 36334117; 37035589; 37266087; 38800819
F5 Individual case report	1	39821937
F5 No randomized clinical comparison	1	39367640
F5 Nonrandomized or uncontrolled report	30	35643988; 35718985; 35855670; 36540630; 36892335; 37123622; 37334389; 37450665; 37465030; 37822409; 38028021; 38260185; 39704133; 40017393; 40150483; 40402866; 40921777; 41065147; 41243364; 41425230; 41520318; 41715284; 42137254; 42164086; 42286046; 42312007; 42325492; 42523891; 42539967; 40469551
F5 Nonrandomized/fixed-side protocol	4	39649763; 40565818; 41211201; 41460004
F5 Preclinical/clinical case demonstration	1	39989893

Recorded principal reason	n	Excluded report PMIDs
F5 Predictable alternating allocation	1	42830871
F5 Random sampling rather than clinical allocation	1	28978136
F5 Randomized intervention allocation not reported	11	23293530; 25709488; 26766918; 29391818; 30333466; 38887212; 38954752; 38962491; 39023837; 39082657; 39340192
F5 Randomized photographic assessment, not treatment	1	39645648
F5 Retrospective nonrandomized series	1	40692693
F5 Uncontrolled clinical series	2	30778526; 32755040
F5 Uncontrolled technique series	1	26644735
F6 No administered target preparation	1	40243133
F7 Periocular volume restoration	1	41345306
F7 Structural hand-volume restoration	1	31568288
F7 Structural nasolabial correction	1	20936295
F7 Structural nasolabial-fold correction	2	37038451; 40378267
F7 Structural volume outcome	1	40236885
F8 Delivery/localization assessment only	2	39960562; 41000432
F8 Filler-localization investigation	1	25018646
F5 Noncomparative open-label study despite RCT indexing	1	26618460
F7 Structural nasolabial-fold filling without separate eligible skin-quality outcome	1	31343447
F1 Technique letter without randomized study	1	20517074
F4 Ordinary topical conditioned medium	1	39845504
F5 Nonrandomized indication-defined groups	1	22984657
F5 Single clinical case	1	33209005
F5 Fixed treatment sides despite randomized label	1	35531796
F4 Ordinary topical plant extract in human component	1	36271377
F5 Fixed treatment sides	1	37583485
F3 Mouse and cell study	1	39703536
F3 Rat biomaterial study	1	40556787
F3 Mouse microneedle study	1	41074154
F5 Single clinical case	1	40571666
F1 Secondary/review/editorial/technique or companion material	23	15041891; 16413906; 23751427; 25417575; 28394860; 30019474; 31408682; 32976175; 34191774; 34719082; 35066552; 35218302; 35253957; 35575902; 36762386; 38140737; 38169140; 38348634; 40354651; 40954315; 41348126; 41990346; 30204744
F2 Population or indication outside scope	1	36735798
F3 Nonhuman/preclinical evidence	2	38756018; 38816551

Recorded principal reason	n	Excluded report PMIDs
F4 Ordinary topical/nonpenetrating exposure	6	29240854; 29601621; 31064236; 38146634; 38336313; 42696342
F5 Nonrandomized/no usable randomized contrast	44	16971362; 19207331; 22672272; 23135654; 24002145; 24962508; 25738851; 25958879; 25973564; 28127660; 28807118; 29160000; 29768259; 30195260; 30942947; 31322524; 31621189; 32272518; 32540414; 32608166; 32757356; 32976172; 33131170; 34133833; 34606674; 35348282; 35552477; 36156843; 36184846; 36514912; 36988469; 38288943; 38544427; 38558364; 38590269; 38764417; 40249527; 40583534; 40711910; 40996202; 41100375; 42383386; 42522478; 33899800
F6 Target preparation not contrasted	8	23057533; 28574158; 29320587; 29356271; 29682870; 31033162; 33687052; 34920819
F7 Isolated structural filling/volume endpoint	9	28902035; 29761211; 30607570; 34232000; 35642570; 37265096; 41662390; 41820616; 42448833

Unresolved sources and historical scope

PMID	Current unresolved issue
32655245	Mean age 24.36 years without an explicit lower bound/adult-only criterion; adult eligibility remains unconfirmed.
41800727	Coin-toss wording alongside an observational case series and within-side tests; a randomized treatment-effect analysis remains unresolved. Current re-access failed.
42872269	PDLLA left and CaHA right in all three participants despite randomized wording; randomized product assignment is not established.
36606396	Section 2.1 describes retrospective treatment-defined groups; Section 3.1 uses randomized wording. Prospective random allocation remains unresolved. DOI 10.1111/jocd.15501.
41361144	PN trial includes crow’s-feet efficacy, so is not pain-only. Individual age range or explicit adult-only eligibility is absent; mean age alone is not used to confirm eligibility. DOI 10.1007/s00266-025-05320-x.
20076916	Original Portuguese text states random grouping, but selected between-treatment skin-quality estimate and adult-only eligibility are unresolved. Methods; Results: 15 volunteers allocated 4/6/5; qualitative photographs and tone observations; no defined quantitative skin-quality contrast.
33437408	Comparative laser-phoresis report without verified random treatment allocation. Laser biorevitalization, 85 women and age-defined controls.
26644738	Paired CaHA hand comparison without verified randomized allocation. Materials and Methods: Treatment, Assessments.
18833043	Eligibility could not be confirmed because the complete report remained unavailable or the intervention/report type was insufficiently specified.
21086592	Eligibility could not be confirmed because the complete report remained unavailable or the intervention/report type was insufficiently specified.
22256620	Eligibility could not be confirmed because the complete report remained unavailable or the intervention/report type was insufficiently specified.
32129049	Eligibility could not be confirmed because the complete report remained unavailable or the intervention/report type was insufficiently specified.

The earlier historical S4 entry is retained only as historical context. Reports with unresolved design or adult eligibility and the four access-unresolved records remain separate from the exclusion log and were not assigned result-level risk-of-bias judgments.
